# The Potential Dual Role of H2.0-like Homeobox in the Tumorgenesis and Development of Colorectal Cancer and Its Prognostic Value

**DOI:** 10.1155/2023/5521544

**Published:** 2023-09-09

**Authors:** Shuo Chen, Lin Zhang, Kai Wang, Jizhen Huo, Siqi Zhang, Xipeng Zhang

**Affiliations:** ^1^Department of Colorectal Surgery, Tianjin Union Medical Center, Hongqiao District, Tianjin 300121, China; ^2^Department of Cardiothoracic Surgery, Army Hospital of the 80th Group, Weicheng District, Weifang 261021, Shandong, China; ^3^Nankai University, No. 94 Weijin Road, Nankai District, Tianjin 300074, China

## Abstract

**Background:**

*H2.0-like homeobox* (*HLX*) is highly expressed in several hematopoietic malignancies. However, the role of *HLX* in the carcinogenesis and progression of colorectal cancer (CRC) patients has rarely been reported.

**Methods:**

In this study, the data were collected from The Cancer Genome Atlas and Gene Expression Omnibus databases. The diagnostic value of *HLX* was analyzed by the R package “pROC.” The overall survival was estimated using the “survival” and “survminer” packages. A nomogram was established to predict 1-, 3-, and 5-year overall survival of CRC patients. The CIBERSORT software was employed to calculate the relative proportions of 22 immune cells.

**Results:**

*HLX* expression was downregulated in CRC patients. Remarkably, *HLX* expression was increased with stage (stage I–stage III) of CRC, and the CRC patients with high *HLX* expression exhibited a poor prognosis. The promoter methylation level of *HLX* was prominently increased in CRC samples compared to paracancerous samples. We also found that the six miRNAs target *HLX* genes, leading to its downregulation, and *HLX* expression had a negative correlation with its downstream target gene *BRI3BP* in both CRC and normal samples. Finally, we found that the 12 immune infiltrating cells were observably different between high and low *HLX* expression groups. The *HLX* had a significant positive correlation with 8 immune checkpoints (PD-1 (PDCD1), CTLA4, PDL-1 (CD274), PDL-2 (PDCD1LG2), CD80, CD86, LAG3, and TIGIT) expressions.

**Conclusion:**

*HLX* probably played a carcinostasis role in the early stages of CRC but exhibited a cancer-promoting effect in the advanced stages. Meanwhile, *HLX* could serve as a reliable prognostic indicator for CRC.

## 1. Introduction

Colorectal cancer (CRC) is one of the leading causes of tumor-related death in the world, and its mortality accounts for 9.4% of cancer deaths worldwide [1, 2]. In 2020, approximately 1.9 million new cases of CRC were diagnosed, and about 900,000 deaths occurred in the world [2]. The morbidity of CRC has been continually rising in many medium or high human development index countries, such as South Eastern Europe, South America, and Eastern Europe[3]. In addition, about 25% of CRC patients have developed metastatic disease at initial diagnosis, and almost 30% of CRC patients with early-stage disease eventually develop metastatic disease [1, 4, 5]. The most common metastatic site of CRC was the liver, followed by the lung, distant lymph nodes, and peritoneum [6]. It has been documented that aberrant expression of genes is involved in the development and progress of CRC. Wang et al. have revealed that *TUG1* knockdown could inhibit the proliferation, invasion, and migration of CRC cells *in vitro* [7]. The mutations of *KRAS*, *p53*, *SMAD4*, and *BRAF* increase the risk of distant metastasis of CRC [8]. Accordingly, further exploration of the molecular mechanisms of CRC may contribute to diagnosis and treatment of CRC at an early stage.

*H2.0-like homeobox* (*HLX*) belongs to the family of NKL homeobox genes. NKL homeobox genes are key regulators of essential processes, such as differentiation, proliferation, and apoptosis, and their expression is cell type-specific [9]. *HLX* is highly expressed in hematopoietic progenitors and lowly expressed in activated lymphocytes [10], and it is a downstream mediator of hepatocyte growth factor (HGF)/c-met induction of cell survival, cell proliferation, and trophoblast migration [11]. Murthi et al. have found that the low *HLX1* expression is associated with abnormal placental development in idiopathic fetal growth restriction [12]. Previous studies have shown that *HLX* is correlated with the initiation and progression of tumor. In gastric cancer, the low *HLX* expression is tightly associated with the expression of *T-bet* and *RUNX3* [13]. Liu et al. have indicated that the level of *HLX1* mRNA is remarkably decreased in hepatocellular carcinoma tissues compared to adjacent nontumorous tissues and downregulation of *HLX1* could promote the invasion, migration, and proliferation of HCC cells [14]. In addition, *HLX* is highly expressed in acute myeloid leukemia (AML) [15, 16]. In zebrafish and human hematopoietic stem progenitor cells (HSPCs), *HLX* overexpression could block myeloid differentiation by regulating metabolic pathways in hematopoietic cells [17]. However, to our knowledge, the potential role of *HLX* in CRC has rarely been reported.

Thus, in this study, we explored the role of *HLX* expression in carcinogenesis and the progression of CRC through the methods of bioinformatics research. Our study could provide valuable information for researchers regarding diagnosis and treatment of CRC at an early stage.

## 2. Methods

### 2.1. Data Collection

The mRNA expression profiling data of 623 CRC patients (colon adenocarcinoma (COAD) and rectum adenocarcinoma (READ)) along with the corresponding clinical information were collected from The Cancer Genome Atlas (TCGA, https://tcga-data.nci.nih.gov/tcga/) database. These 632 patients included a total of 638 CRC samples and 51 paracancerous samples, and 590 patients contained complete survival information. The methylation 450K chip data of 408 CRC samples (COAD + READ) were downloaded.

The GSE41258 and GSE17538 datasets were downloaded from the Gene Expression Omnibus (GEO, https://www.ncbi.nlm.nih.gov/geo/) database. GSE41258 included 186 CRC and 54 normal samples, while GSE17538 contained 244 CRC samples (232 with complete survival information).

### 2.2. Analysis of the Diagnostic Value of HLX

The TCGA cohort was employed to make a receiver operating characteristic (ROC) curve through the R language (Version 4.2.1, the same below) function package pROC to analyze the diagnostic value of the *HLX *gene.

### 2.3. Survival Analysis

The R language survival package and survminer package were used to estimate the overall survival of different groups based on the Kaplan–Meier method, and the log-rank test was used to test the significance of differences in survival between different groups. The multivariate Cox regression model was used to analyze whether the target genes could predict the survival of CRC patients.

### 2.4. Gene Set Enrichment Analysis (GSEA)

In the TCGA cohort, the samples were split into *HLX* high and low expression groups according to the median expression of *HLX* using the R language limma function package. Then, the differentially expressed genes (DEGs) between the two groups were screened based on the |log2FC| > 0.5 and *p*. adjust <0.05. The DEGs were subjected to GSEA using the R language function package ReactomePA and ClusterProfiler [18, 19].

### 2.5. Establishment of the Nomogram Prediction Model

To predict 1-, 3-, and 5-year overall survival of CRC patients, the R language rms (https://CRAN.R-project.org/package=rms) package was used to establish the nomogram for all independent prognostic factors identified by multivariate Cox regression analysis. The calibration curve of the nomogram was plotted, and the relationship between the nomogram predicted probability and actual incidence was observed. For each patient, three lines were drawn upward to determine the points obtained from each factor in the nomogram. The sums of these points were located on the “Total Points” axis, and a line was then drawn from the total points axis to determine the probability of a 1-, 3-, and 5-year survival rate for CRC patients.

### 2.6. Immune Cell Infiltration

The software CIBERSORT [20] was employed to calculate the relative proportions of 22 immune cells in the samples. CIBERSORT software characterizes the composition of immune infiltrating cells according to gene expression matrices using a deconvolution algorithm based on a preset set of 547 barcode genes. The immune scores of the samples were calculated using the “estimate” function package.

### 2.7. Statistical Analysis

The Wilcoxon rank sum test was used to compare gene expression differences and infiltration differences of immune cells among different groups using the UALCAN online database [21]. *p*  <  0.05 was considered statistically significant. All the above statistical analyses were performed using the R software.

## 3. Results

### 3.1. HLX Expression Was Closely Correlated with Carcinogenesis and Progression of CRC

**Figure 1:**
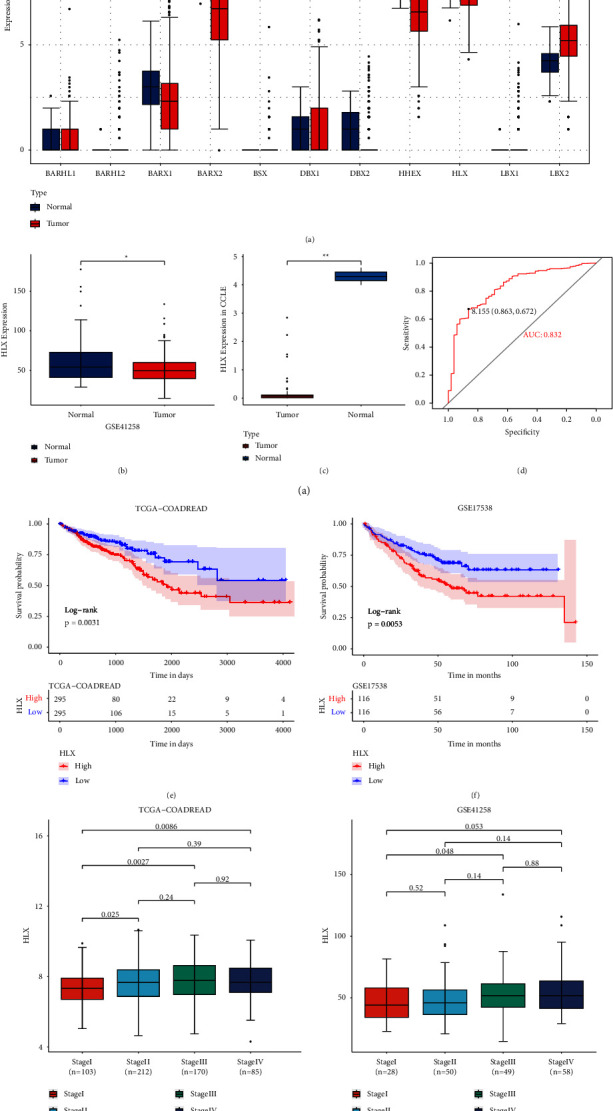
*HLX* expression was closely correlated with the prognosis of CRC patients. (a) The 11 genes (*DBX1*, *DBX2*, *BSX*, *BARX1*, *BARX2*, *BARHL1*, *BARHL2*, *LBX1*, *LBX2*, *HLX*, and *HHEX*) expression in CRC samples. (b) *HLX* expression in CRC samples in the GSE41258 dataset. (c) The expression of *HLX* in CRC and normal samples in the CELL cell line database. (d) The curve for the diagnostic value of *HLX*. (e, f) The KM survival curves of *HLX* high and low expression groups in the TCGA and GSE17538 cohorts. (g, h) *HLX* expression in a different stage of CRC patients in TCGA and GSE41258 cohorts.

First, in the TCGA cohort, we analyzed the expression of 11 genes (*DBX1*, *DBX2*, *BSX*, *BARX1*, *BARX2*, *BARHL1*, *BARHL2*, *LBX1*, *LBX2*, *HLX*, and *HHEX*) which belong to the subfamily in which *HLX* is located within the NKL family. As shown in Figure 1(a), the *HLX* expression was prominently downregulated in CRC samples. In the GSE41258 dataset, *HLX* expression was also decreased in CRC samples (Figure 1(b)). In addition, we analyzed the expression of *HLX* in CRC and normal samples in the CELL cell line database. The results unequivocally demonstrated a remarkable and substantial downregulation of *HLX* expression in CRC cell lines, which was visually represented in Figure 1(c).

ROC curves showed that *HLX* might serve as a potential diagnostic marker for CRC in the TCGA cohort (AUC = 0.832, Figure 1(d)). The CRC patients with high *HLX* expression exhibited poor prognosis in both the TCGA and GSE17538 cohorts (Figures 1(e) and 1(f)). Moreover, we found that the *HLX* expression increased as the stage increased from stage I to stage III (Figures 1(g) and 1(h)). These results indicated that the *HLX* expression was closely correlated with carcinogenesis and progression of CRC.

### 3.2. Nomogram Model Could Effectively Predict the Prognosis of CRC Patients

**Figure 2:**
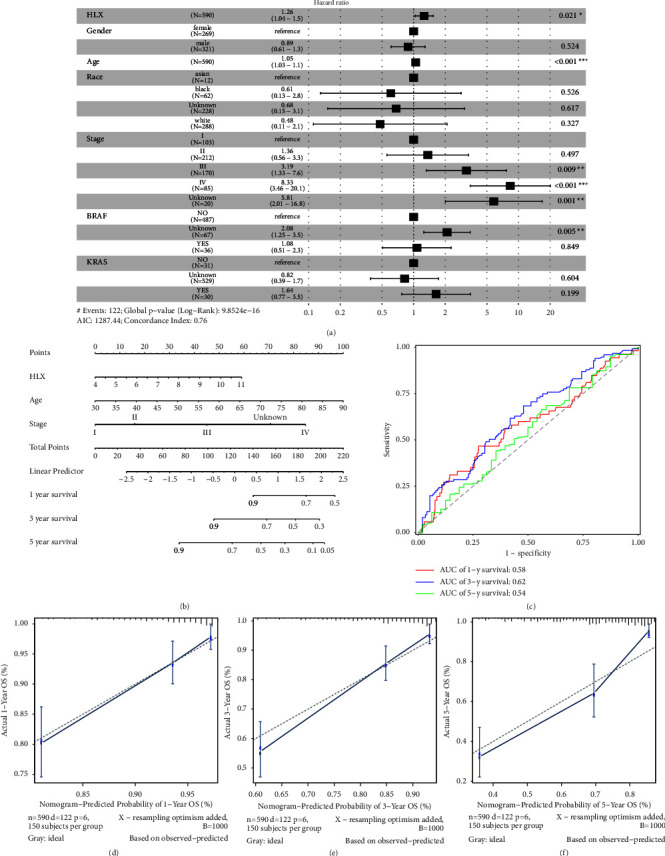
Nomogram model could effectively predict the prognosis of CRC patients. (a) Forest plot of multivariate Cox regression analysis. (b) Nomogram to predict the probability of 1-, 3-, and 5-year overall survival in CRC patients. (c) Curve of the ROC. (d–f) Calibration curves of the nomogram to predict the probability of 1-, 3-, and 5-year overall survival in CRC patients.

Multivariate Cox regression analysis including gender, age, stage, race, BRAF mutation, and KRAS mutation showed that *HLX* genes could be used as prognostic factors in CRC (Figure 2(a)). In addition, three independent prognostic factors, *HLX*, age, and TNMstage, were used to construct the nomogram model (Figure 2(b)). In the TCGA cohort, the AUC of 1-, 3-, and 5-year overall survival was 0.58, 0.62, and 0.54, respectively (Figure 2(c)). The results suggested that the nomogram model could effectively predict the prognosis of CRC patients. Meanwhile, the corrected curve in the calibration plot was relatively close to the ideal curve (a 45-degree line passing through the origin of the coordinate axis and with a slope of 1), indicating that the prediction agreed well with the real outcome (Figures 2(d)–2(f)).

### 3.3. Increased Promoter Methylation Repressed the Expression of HLX in CRC Patients

**Figure 3:**
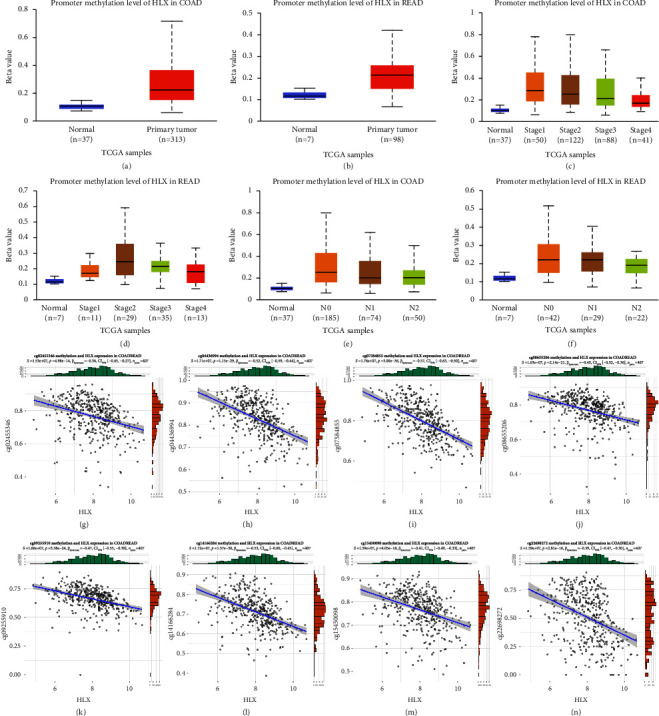
The CRC patients with a high promoter methylation level of *HLX*. (a, b) The promoter methylation level of *HLX* in COAD and READ. (c–f) The promoter methylation level of *HLX* in different cancer development stages and different lymph node metastases of COAD and READ. (g–n) The correlation of eight methylation sites with *HLX* mRNA.

Furthermore, we analyzed the promoter methylation level of *HLX* in TCGA-COAD and TCGA-READ using the online analysis tool UALCAN (http://ualcan.path.uab.edu/) [21]. As shown in Figures 3(a) and 3(b), the promoter methylation level of *HLX* was prominently increased in cancer tissues compared to paracancerous tissues in COAD and READ patients. Remarkably, compared to paracancerous tissues, the promoter methylation level of *HLX* was also prominently increased in different cancer development stages and different lymph node metastases of COAD and READ (Figures 3(c)–3(f)).

The TCGA CRC methylation data were used to explore which sites in the promoter region of *HLX* that increased methylation level would affect the silencing of the *HLX* gene. The 18 cg sites with high methylation levels were screened by methylation level mean >0.5 and median >0.5. Further screening was performed according to the different sites methylation levels and *HLX* expression correlation absolute values >0.3 and *p*  <  0.05. The results showed that eight methylation sites were associated with the downregulation of *HLX* expression (Figures 3(g)–3(n), Table 1).

### 3.4. Prediction of the Upstream miRNA and Downstream Target Gene of HLX

**Figure 4:**
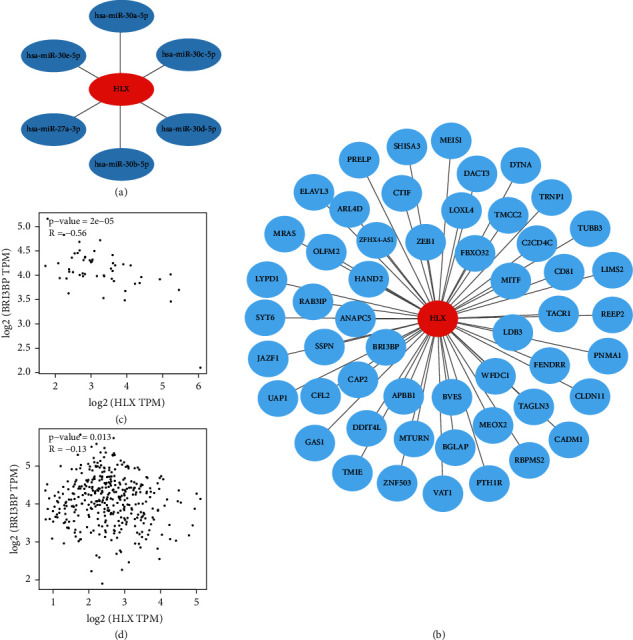
Regulation of *HLX* translation by miRNAs. (a, b) Regulatory relationships visualized by the Cytoscape. (c, d) The correlation of *HLX* with *BRI3BP*.

The TargetScan (https://www.targetscan.org/vert_80/) database was used to predict the miRNAs targeting *HLX*, and 11 miRNAs were found to specifically bind to the 3′UTR end of *HLX*. The mirDIP (https://ophid.utoronto.ca/mirDIP/) database was employed to screen miRNAs potentially interacting with *HLX*, and 23 miRNAs were found according to the top 5% screening. A total of six miRNAs (hsa-mir-30b-5p, hsa-mir-30d-5p, hsa-mir-30c-5p, hsa-mir-30a-5p, hsa-mir-30e-5p, and hsa-mir-27a-3p) were found to potentially target *HLX*, leading to *HLX* downregulation (Figure 4(a), Table 2).

Subsequently, the GRNdb (https://www.grndb.com/) database was used to inquire *HLX*'s target genes, and results were visualized by Cytoscape (Figure 4(b)). We found a possible binding site for the transcription factor HLX around 20 bp upstream of the BRI3BP promoter (Table S1) via FIMO (https://meme-suite.org/meme/tools/fimo). We also found that the *HLX* expression had a negative correlation with *BRI3BP* in both CRC samples and normal samples (Figures 4(c) and 4(d)). The results suggested that the *HLX* negatively regulated the *BRI3BP* in CRC.

### 3.5. The Result of GSEA

**Figure 5:**
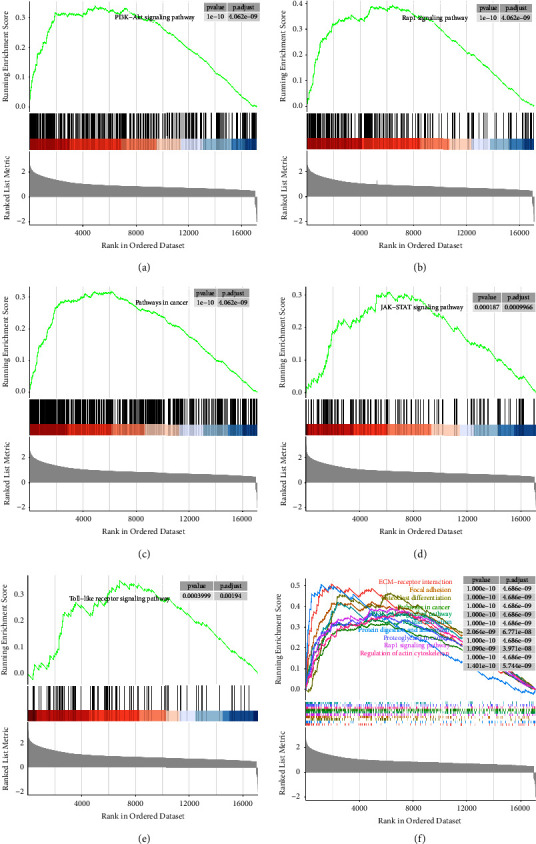
The result of GSEA. (a–e) The PI3K-Akt signaling pathway, Rap1 signaling pathway, pathways in cancer, JAK-STAT signaling pathway, and toll-like receptor signaling pathway were significantly activated in the *HLX* high expression group. (f) The top 10 significantly enriched pathways.

The GSEA showed that 138 pathways were prominently activated, such as the PI3K-Akt signaling pathway, Rap1 signaling pathway, pathways in cancer, JAK-STAT signaling pathway, and toll-like receptor signaling pathway (Figures 5(a)–5(e)), and 8 pathways were inhibited in the *HLX* high expression group compared to the *HLX* low expression group (Table S2). The top 10 significantly enriched pathways are displayed in Figure 5(f).

### 3.6. HLX Involved in the Immune Cell Infiltration in CRC Patients

**Figure 6:**
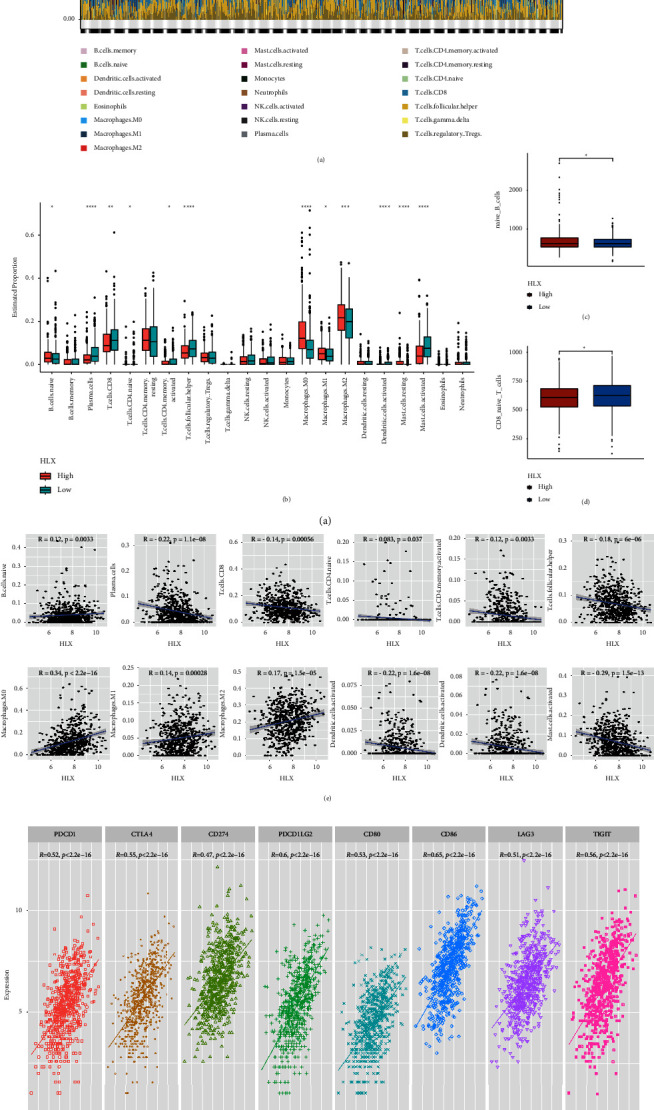
*HLX* could be involved in the immune cell infiltration in CRC patients. (a) The relative proportions of 22 immune infiltrating cells in CRC samples in the TCGA cohort. (b) The content of 22 immune infiltrating cells in high and low *HLX* expression groups using the CIBERSORT algorithm. The content of B.cells.naive (c) and T.cells.CD8 (d) in high and low *HLX* expression groups using the Xcell method. (e) The correlation of HLX with 12 immune infiltrating cells. (f) The correlation of HLX with 8 immune checkpoints (PD-1 (PDCD1), CTLA4, PDL-1 (CD274), PDL-2 (PDCD1LG2), CD80, CD86, LAG3, and TIGIT).

The relative proportions of 22 immune infiltrating cells in CRC samples in the TCGA cohort were calculated by the CIBERSORT algorithm (Figure 6(a), Table S3). The 12 immune infiltrating cells (B.cells.naive, Plasma.cells, T.cells.CD8, T.cells.CD4.naive, T.cells.CD4.memory.activated, T.cells.follicular.helper, Macrophages.M0, Macrophages.M1, Macrophages.M2, Dendritic.cells.activated, Mast.cells.resting, and Mast.cells.activated) were significantly differential between *HLX* high and low expression groups (Figure 6(b)). To avoid possible errors, we additionally recalculated the content of immune infiltrating cells in CRC samples from the TCGA cohort using the Xcell method. The results showed that the contents of B.cells.naive (Figure 6(c)) and T.cells.CD8 (Figure 6(d)) were significantly different between the high and low *HLX* expression groups, and the trend was consistent with that found using the CIBERSORT algorithm.

Next, we used the results of CIBERSORT to further analyze the Spearman correlation between the *HLX* and the 12 significantly different immune infiltrating cells. The results showed that *HLX* expression exhibited a negative correlation with Plasma.cells, T.cells.CD8, T.cells.CD4.naive, T.cells.CD4.memory.activated, T.cells.follicular.helper, Dendritic.cells.activated, Mast.cells.resting, and Mast.cells.activated and had a positive association with B.cells.naive, Macrophages.M0, Macrophages.M1, and Macrophages.M2 (Figure 6(e)). Finally, we analyzed the correlation between HLX expression and immune checkpoints. The *HLX* expression had a significant positive correlation with the expression of 8 immune checkpoints (PD-1 (PDCD1), CTLA4, PDL-1 (CD274), PDL-2 (PDCD1LG2), CD80, CD86, LAG3, and TIGIT) (Figure 6(f)).

## 4. Discussion

In this study, we explore the association between *HLX* and carcinogenesis and progression of CRC by systematically collecting and analyzing the clinical and genomic data of CRC patients. We found that *HLX* was downregulated in CRC samples compared to paracancerous samples, and *HLX* expression increased as stage increased from stage I totage III. The CRC patients with high *HLX* expression exhibited a worse prognosis. We also found that the downregulation of *HLX* was regulated by six miRNAs, and the *HLX* negatively regulated its downstream target gene *BRI3BP* in CRC.

Previous studies have demonstrated that *HLX* is abnormally expressed in multiple cancers. For example, in anaplastic large cell lymphoma and diffuse large B-cell lymphoma, the *HLX* was overexpressed in cancer tissues [22, 23]. In this study, we found that *HLX* expression was prominently downregulated in CRC samples. Kawahara et al. have reported that the *HLX* was highly expressed in 87% of acute myeloid leukemia patients, and patients with high *HLX* expression had an inferior prognosis [24]. In addition, the inhibition of *HLX* expression could reduce the proliferation and clonogenicity of leukemia cells and prolong the survival rate [24]. Zhu et al. have indicated that low *HLX* expression could reduce the proliferation of acute myelogenous leukemia cells by regulating the JAK/STAT signaling pathway [25]. This evidence suggested that *HLX* downregulation might be involved in the progression of some tumors. In this study, we also found that the *HLX* expression was increased with tumor stage (stage I–stage III) in CRC, and the high expression of *HLX* was correlated with poor prognosis of the CRC patients. These results showed that *HLX* might play different roles, as a cancer-suppressor gene or a cancer-promoting gene, depending on the stage of CRC, and clinicians might potentially use *HLX* expression as a biomarker to predict patient outcomes and tailor treatment plans accordingly.

Promoter DNA methylation generally represses the transcription expression by regulating the binding of transcription factors [26], and DNA methylation is a critical epigenetic process that contributes to the progression of CRC [27]. Since *HLX* was downregulated in CRC samples, we analyzed whether the promoter methylation level of *HLX* was responsible for *HLX* gene silencing. We found that the promoter methylation level of *HLX* was prominently increased in cancer tissues compared to paracancerous tissues in CRC patients. These results suggested that the downregulation of *HLX* in CRC was associated with its promoter methylation level. Moreover, miRNAs could lead to degradation or translational repression of the mRNA by targeting the form of complementary mRNAs [28]. In diffuse large B-cell lymphoma, the expression of *HLX* was regulated by EBV-mediated *STAT3* activation [29], and *STAT3* was also confirmed to regulate the expression of *HLX* in Hodgkin lymphoma [30]. In this study, we found that the downregulation of *HLX* was regulated by six miRNAs, and the *HLX* negatively regulated its downstream target gene *BRI3BP* in CRC. *BRI3BP* was mapped to chromosome 12q24.2-qter in humans, and it was highly expressed in the brain, liver, and kidney, and *BRI3BP* might be involved in apoptosis [31]. *BRI3BP* overexpression promoted apoptosis in 293T cells (human embryonic kidney) challenged with etoposide (an anticancer agent). In addition, it has been reported that *BRI3BP* expression is decreased in cancer samples [32]. Thus, we infer that *HLX* might regulate the expression of *BRI3BP* to be involved in the carcinogenesis and progression of CRC.

GSEA showed immune-related signaling pathways were significantly activated in the *HLX* high expression group compared to the *HLX* low expression group, such as the PI3K-Akt signaling pathway and the Rap1 signaling pathway. These findings might inform the development of targeted therapies that could improve CRC patient outcomes. Subsequently, we investigated the effect of *HLX* on immune cell infiltration in CRC. We found that the *HLX* expression exhibited a negative correlation with Plasma.cells, T.cells.CD8, T.cells.CD4.naive, T.cells.CD4.memory.activated, and T.cells.follicular.helper and had a positive association with B.cells.naive. It has been reported that the *HLX* expression is involved in the activation and growth of T cells [33]. The *HLX* overexpression could disturb the development of B cells and T cells in murine lymphocyte [34] and inhibit CD4+T cells development and disrupt thymic involution in transgenic mice [35]. The above commendations indicated that the *HLX* probably regulates the infiltration of B cells and T cells, thereby influencing the prognosis of CRC patients.

Finally, we discovered that the *HLX* expression had a significant positive correlation with the expression of PD-1, CTLA4, PDL-1, PDL-2, and LAG3. The interaction between PD-1 and its ligand PD-L1 suppresses T cell proliferation and cytokine release. As a result, PD-1 modulates immunological responses in reverse, allowing tumor cells to evade immune surveillance [36]. Previous studies showed that PD-L1 expression was higher in metastatic CRC than in primary tumors [37]. Wang et al. have demonstrated that the combination of fruquintinib and antiPD-1 could synergistically inhibit the progression of CRC and alter the tumor microenvironment in favor of anti-tumor immune responses [38]. Regorafenib combined with antiPD-1 could enhance M1 macrophage differentiation and activation and continuously inhibit Treg cell infiltration to improve antitumor activity [39]. CTLA-4 was an inhibitory immune checkpoint that was overexpressed in the CRC tissues and the CRC cell line SW480, and capecitabine treatment resulted in a significant downregulation of CTLA-4 expression in SW480 cells [40]. LAG3 was also found to be overexpressed in microsatellite instability (MSI) tumors compared to microsatellite stability (MSS), making it an excellent target for immunotherapy in MSI CRC [41, 42]. It has been reported that the favezelimab (LAG-3 antibody) in combination with pembrolizumab has promising antitumor activity in CRC patients [43]. Considering the positive correlation between *HLX* expression and PD-1, CTLA4, PDL-1, and LAG3 expressions in CRC patients, *HLX* might play a role in regulating immune checkpoints and influencing the response to immunotherapy. Therefore, combining chemotherapy with immunotherapy approaches, such as PD-1 and CTLA-4 inhibitors, might be a promising strategy for improving the treatment of CRC.

Despite the fact that we researched and merged data from several sources, the current study has numerous limitations. Firstly, while bioinformatics analysis provided us with some valuable insights regarding *HLX* in CRC, all the samples enrolled in this research were retrospective, the study did not include experimental validation of the findings, and a prospective study should be applied to validate the results. In addition, the study focused primarily on mRNA expression profiling and methylation data for *HLX* and did not consider other genetic or epigenetic factors that might play a role in CRC development and progression. This might limit the ability to fully understand the molecular mechanisms of *HLX* in CRC and develop effective treatment strategies for CRC. Thus, studies with large clinical sample size, such as electronic medical records or hospital databases, are warranted in the near future.

## 5. Conclusion

Our study demonstrated that *HLX* probably exhibits dual roles at different stages of CRC; HLX might play a carcinostasis role in the early stage of CRC, but exhibit cancer-promoting effects in the advanced-stage. Moreover, the downregulation of *HLX* was regulated by six miRNAs and the *HLX* negatively regulated its downstream target gene *BRI3BP* in CRC. Our findings provide valuable insights into the molecular mechanisms underlying CRC development and progression and could potentially inform the development of more effective treatment strategies for CRC patients.

## Figures and Tables

**Table 1:** Correlation of *HLX* and methylation in the TCGA cohort.

	Rho	Spearman *p* value
cg07584855	−0.5467062	4.26*E* − 33
cg04436994	−0.470507377	8.23*E* − 24
cg14166284	−0.467110527	1.89*E* − 23
cg09255910	−0.393466138	1.60*E* − 16
cg22698272	−0.377589256	3.07*E* − 15
cg15450098	−0.36694579	2.03*E* − 14
cg08655206	−0.34967184	3.78*E* − 13
cg02455346	−0.309801879	1.67*E* − 10

**Table 2:** miRNA in TargetScan and mirDIP database.

TargetScan	mirDIP
hsa-miR-27b-3p	hsa-miR-30b-5p
hsa-miR-27a-3p	hsa-miR-30d-5p
hsa-miR-128-3p	hsa-miR-30c-5p
hsa-miR-3681-3p	hsa-miR-30a-5p
hsa-miR-216a-3p	hsa-miR-30e-5p
hsa-miR-30c-5p	hsa-miR-27a-3p
hsa-miR-30b-5p	hsa-miR-515-3p
hsa-miR-30d-5p	hsa-miR-519e-3p
hsa-miR-30a-5p	hsa-miR-92a-1-5p
hsa-miR-30e-5p	hsa-miR-127-3p
hsa-miR-760	hsa-miR-1537-3p
	hsa-miR-1538
	hsa-miR-676-3p
	hsa-miR-3157-3p
	hsa-miR-33b-3p
	hsa-miR-1470
	hsa-miR-4649-5p
	hsa-miR-937-3p
	hsa-miR-6729-5p
	hsa-miR-371a-3p
	hsa-miR-8055
	hsa-miR-6853-5p
	hsa-miR-4315

## Data Availability

The data that support the findings of this study are available in TCGA (https://tcga-data.nci.nih.gov/tcga/) database, Gene Expression Omnibus (GEO, https://www.ncbi.nlm.nih.gov/geo/) database, UALCAN online database (https://ualcan.path.uab.edu/), the TargetScan (https://www.targetscan.org/vert_80/) database, the mirDIP (https://ophid.utoronto.ca/mirDIP/) database and the GRNdb (https://www.grndb.com/) database.

## Additional Points

(1) *HLX* might play a carcinostasis role in the early stage of CRC, but exhibit a cancer-promoting effect in advanced stage. (2) Promoter methylation of *HLX* represses the expression of *HLX* in CRC patients. (3) The downregulation of *HLX* was regulated by six miRNAs and the *HLX* negatively regulated its downstream target gene *BRI3BP* in CRC.

## Supplementary Materials

Supplementary Materials Table S1: The result of FIMO prediction. Table S2: Detailed results of significantly enriched pathways in *HLX* high expression group. Table S3: The relative proportions of 22 immune infiltrating cells of CRC samples in TCGA cohort.
