# Supplementary material for: The Potential Dual Role of H2.0-like Homeobox in the Tumorgenesis and Development of Colorectal Cancer and Its Prognostic Value
Source: Can J Gastroenterol Hepatol. 2023 Sep 9;2023:5521544. doi: 10.1155/2023/5521544 (PMC10505080; doi:10.1155/2023/5521544)
Supplement: Supplementary Materials — Table S1: The result of FIMO prediction. Table S2: Detailed results of significantly enriched pathways in HLX high expression group. Table S3: The relative proportions of 22 immune infiltrating cells of CRC samples in TCGA cohort. [file 5521544.f1.zip › Table S1.docx]

**Table S1** The result of FIMO prediction

| motif_id | motif_alt_id | sequence_name | start | stop | strand | score | p-value | q-value | matched_sequence |
| --- | --- | --- | --- | --- | --- | --- | --- | --- | --- |
| 1 | NYTWCCCAYAATGCAHYRCDN | hg38_knownGene_ENST00000341446.9 | 2716 | 2736 | - | 7.22424 | 0.000136 | 0.669 | ATCTCCCTGAATGTATTATAA |
| 1 | NYTWCCCAYAATGCAHYRCDN | hg38_knownGene_ENST00000341446.9 | 2343 | 2363 | + | 5.92121 | 0.000239 | 0.669 | cttggcaagaatccatcgcag |
| 1 | NYTWCCCAYAATGCAHYRCDN | hg38_knownGene_ENST00000341446.9 | 2640 | 2660 | - | 5.10303 | 0.000337 | 0.669 | AATAACAAGAAAGCATCAGGC |
| 1 | NYTWCCCAYAATGCAHYRCDN | hg38_knownGene_ENST00000341446.9 | 729 | 749 | - | 3.52121 | 0.000637 | 0.686 | GTTGCCCAGGCTGGAGTGCAG |
| 1 | NYTWCCCAYAATGCAHYRCDN | hg38_knownGene_ENST00000341446.9 | 1243 | 1263 | + | 3.52121 | 0.000637 | 0.686 | gttgcccaggctggagtgcag |
| 1 | NYTWCCCAYAATGCAHYRCDN | hg38_knownGene_ENST00000341446.9 | 911 | 931 | - | 3.0303 | 0.000771 | 0.686 | TTCCCCCACGAAGCCTCCCTT |
| 1 | NYTWCCCAYAATGCAHYRCDN | hg38_knownGene_ENST00000341446.9 | 2212 | 2232 | + | 2.91515 | 0.000806 | 0.686 | gtttggtagaatgccccacat |
| 1 | NYTWCCCAYAATGCAHYRCDN | hg38_knownGene_ENST00000341446.9 | 2145 | 2165 | + | 2.48485 | 0.00095 | 0.707 | aaaaccaattatcctcctctt |
