# Supplementary material for: The Potential Dual Role of H2.0-like Homeobox in the Tumorgenesis and Development of Colorectal Cancer and Its Prognostic Value
Source: Can J Gastroenterol Hepatol. 2023 Sep 9;2023:5521544. doi: 10.1155/2023/5521544 (PMC10505080; doi:10.1155/2023/5521544)
Supplement: Supplementary Materials — Table S1: The result of FIMO prediction. Table S2: Detailed results of significantly enriched pathways in HLX high expression group. Table S3: The relative proportions of 22 immune infiltrating cells of CRC samples in TCGA cohort. [file 5521544.f1.zip › Table S2.docx]

**Table S2** Detailed results of significantly enriched pathways in HLX high expression group

| ID | Description | setSize | enrichmentScore | NES | pvalue | p.adjust | qvalue | rank | leading_edge | core_enrichment |
| --- | --- | --- | --- | --- | --- | --- | --- | --- | --- | --- |
| hsa04512 | ECM-receptor interaction | 82 | 0.5090895 | 2.84592606 | 1.00E-10 | 4.69E-09 | 2.39E-09 | 1906 | tags=41%, list=11%, signal=37% | 1292/1284/1291/1278/1277/3678/1293/22801/3910/7450/1282/3913/9900/7058/7148/3371/3339/2335/3915/3693/3679/7059/7057/3690/63923/9899/3908/1311/3912/3672/7060/3688/8515/5649 |
| hsa04974 | Protein digestion and absorption | 78 | 0.5060227 | 2.80613838 | 1.00E-10 | 4.69E-09 | 2.39E-09 | 1157 | tags=33%, list=7%, signal=31% | 1292/1284/1291/80781/1289/1278/1277/1296/1293/1281/1306/1290/1295/1282/50509/7373/169044/1303/1307/1305/2006/1301/482/1300/1294/255631 |
| hsa05150 | Staphylococcus aureus infection | 46 | 0.5542496 | 2.800248079 | 6.12E-09 | 1.82E-07 | 9.30E-08 | 2396 | tags=50%, list=14%, signal=43% | 715/716/3075/718/721/3684/3689/2209/3383/728/712/6404/719/714/720/2212/2214/2359/713/3683/2213/5724/6403 |
| hsa00532 | Glycosaminoglycan biosynthesis - chondroitin sulfate / dermatan sulfate | 20 | 0.6926049 | 2.776632363 | 4.96E-08 | 1.16E-06 | 5.93E-07 | 4398 | tags=90%, list=26%, signal=67% | 9469/50515/337876/113189/51363/29940/55454/54480/22856/55501/55790/56548/79586/64131/64132/26229/10090/126792 |
| hsa04380 | Osteoclast differentiation | 118 | 0.4654526 | 2.757970251 | 1.00E-10 | 4.69E-09 | 2.39E-09 | 6296 | tags=74%, list=37%, signal=47% | 1435/1513/10000/6688/5600/3554/1436/7040/4286/4791/9021/4772/2209/126014/3690/5293/54/10288/10859/140885/5971/6773/11025/7305/54209/11006/7042/79168/5602/9020/8517/5609/5970/2212/2214/2534/695/2885/207/7132/7297/7186/11024/5336/9846/10454/3937/2213/208/4792/4688/3455/11027/653361/4790/2355/1540/8878/353514/7046/3716/7048/3460/6300/5604/8503/5879/3454/6772/5533/4773/6850/3726/10326/10379/3551/23547/3725/10990/1385/23118/7189/7124/5532/5290/5594/814 |
| hsa05144 | Malaria | 46 | 0.541545 | 2.736060474 | 2.47E-08 | 6.23E-07 | 3.18E-07 | 3212 | tags=54%, list=19%, signal=44% | 2995/5175/6383/7058/7043/7040/7412/3689/6347/7059/7057/3383/1311/4035/7042/6401/7060/975/3082/3683/4615/6403/2532/7097/958 |
| hsa04510 | Focal adhesion | 192 | 0.4247041 | 2.649085871 | 1.00E-10 | 4.69E-09 | 2.39E-09 | 4911 | tags=56%, list=29%, signal=40% | 1292/5159/1284/1291/1278/1277/2324/10398/3678/1293/22801/7424/5155/3910/2316/7450/1282/3913/7058/7094/4638/7148/3371/10000/2335/87/3915/3791/3693/56034/23396/857/3611/6464/3679/64098/7059/7057/2889/399694/3690/63923/2321/5293/3908/7791/60/1311/3912/5228/3672/54776/5602/5156/7423/2318/7414/55742/7060/3688/2534/5063/3082/8515/5154/5649/2885/207/3479/858/208/4660/81/2002/9564/2909/1793/3676/3914/3480/7409/3685/5880/5829/1399/7143/1729/595/3918/5170/7410/896/3381/3680/5579/7408/10319/6714/387/2317/1398/5894/5604/8503/6696/5879/3911 |
| hsa04340 | Hedgehog signaling pathway | 52 | 0.4968152 | 2.563551473 | 4.74E-07 | 6.48E-06 | 3.31E-06 | 4133 | tags=60%, list=24%, signal=45% | 2121/132884/2735/91653/2736/2737/23432/2619/8643/51684/409/1954/50846/6608/5566/57758/374654/23295/1453/84976/1454/57154/408/8945/23288/50937/1455/23291/156/8405/595 |
| hsa04611 | Platelet activation | 115 | 0.420604 | 2.479841187 | 2.06E-09 | 6.77E-08 | 3.46E-08 | 2384 | tags=39%, list=14%, signal=34% | 1278/1277/6915/5739/1281/7450/2771/2983/2982/7094/9002/4638/10000/2149/5600/108/10235/146850/3690/5293/196883/115/3708/60/8605/5742/23533/2207/5566/2212/3688/2534/4846/5330/695/5592/207/6786/111/5336/83706/3937/208/9138/54518 |
| hsa04610 | Complement and coagulation cascades | 64 | 0.4616727 | 2.478349066 | 5.83E-07 | 7.65E-06 | 3.90E-06 | 2281 | tags=39%, list=13%, signal=34% | 715/710/716/2/7450/7056/9002/2149/3075/5327/718/721/3684/5328/3689/728/5054/712/3687/719/714/720/713/11326/624 |
| hsa04015 | Rap1 signaling pathway | 189 | 0.3928412 | 2.444802046 | 1.00E-10 | 4.69E-09 | 2.39E-09 | 6415 | tags=64%, list=38%, signal=40% | 5159/2260/2324/22808/7424/5155/1435/2771/7010/7094/9002/5587/10000/2149/5600/51466/2775/3791/1436/2246/56034/108/10235/3684/3689/7057/2889/2255/25780/2252/3690/84552/2321/5293/196883/115/6494/60/4803/5228/5156/284/7423/25865/2846/4804/3688/5330/3082/5900/5154/2247/207/3479/111/3683/3937/208/9732/54518/285/9564/808/10411/801/83593/2778/7074/3480/7409/113/5880/9863/26037/23094/2250/1399/135/6237/5335/56288/7410/9223/3643/2533/5579/7408/6714/5216/387/6300/5899/1398/107/1902/109/5894/5604/8503/5879/1500/11069/5606/80310/9693/1499/51378/5898/5906/55740/9170/112/57568/5290/1956/5594/10636/2770/2357/64411/9771 |
| hsa05133 | Pertussis | 69 | 0.4462327 | 2.435442685 | 1.08E-06 | 1.14E-05 | 5.84E-06 | 2815 | tags=43%, list=16%, signal=36% | 715/710/716/3678/2771/5600/718/721/3684/3689/929/712/714/5602/720/10392/5970/3688/114548/713/1073/23643/4615/4790/353376/148022/114609/808/3661/801 |
| hsa04330 | Notch signaling pathway | 56 | 0.4509397 | 2.363333157 | 1.94E-06 | 1.80E-05 | 9.20E-06 | 6974 | tags=79%, list=41%, signal=47% | 4854/4855/26508/9253/23493/196403/23462/4242/7090/1857/9612/1387/4853/54567/1856/1487/9794/2033/6310/342371/7091/1840/51107/4851/28514/23385/8650/1488/5664/6868/55534/9541/23220/84441/22938/3280/171558/5663/182/151636/55851/3516/5986/83464 |
| hsa04810 | Regulation of actin cytoskeleton | 204 | 0.3741189 | 2.346720249 | 1.40E-10 | 5.74E-09 | 2.93E-09 | 6823 | tags=66%, list=40%, signal=40% | 5159/2260/10398/3678/22801/22808/5155/4478/4638/10000/2149/2335/2768/87/2246/3693/56034/23396/6387/3684/4628/3679/3689/4629/2255/4627/2252/3690/3984/5293/9459/54434/3687/60/5305/3672/54776/5156/2934/3071/7414/8396/2846/3688/5063/8515/5154/2247/207/3683/10095/624/1073/208/10163/4660/5962/9138/81/10109/28984/9564/2909/1793/3676/23191/7074/7409/7852/3685/3985/2245/5880/5829/1072/623/89846/10093/2250/1399/6237/79837/10297/1729/369/7410/3680/730/7430/6714/5216/387/10096/1398/1902/5894/5604/6548/8503/5879/1132/85366/28964/8874/54961/80310/8936/6093/10152/10458/6654/23365/85464/55740/10552/9170/5290/5747/1956/5594/8976/57180/55845/8394/8826/10092/5062/5605/10298/10097/3674/79784/1129/5295 |
| hsa04145 | Phagosome | 119 | 0.3944846 | 2.340635536 | 1.11E-07 | 2.02E-06 | 1.03E-06 | 7993 | tags=78%, list=47%, signal=42% | 715/9902/84617/3678/7058/7846/718/3693/338382/3684/1514/3689/7059/7057/929/81035/2209/3690/60/1311/11151/2212/7060/3688/2214/537/5878/29927/1778/4973/2213/4688/8685/653361/534/203068/811/527/3916/4074/7097/1781/1536/3685/4481/7280/10333/535/7879/8992/948/64581/529/9146/1783/51606/51143/5879/79659/4360/5869/9114/4842/245972/347733/10381/9554/528/526/3920/10376/6891/51382/10952/1780/10312/23480/23673/9296/84790/53407/9550/5289/71/78989/821/10382/155066/6441/30835/8417/200576/90423 |
| hsa04270 | Vascular smooth muscle contraction | 113 | 0.3947423 | 2.322561253 | 2.39E-07 | 3.57E-06 | 1.82E-06 | 4789 | tags=51%, list=28%, signal=37% | 59/10398/5739/4881/10335/10268/800/2983/2982/10266/5322/4638/3779/2768/4882/108/4628/4629/4627/196883/115/10203/3708/8605/1909/54776/72/5566/5583/5330/5592/185/111/552/94274/4660/9138/808/140465/801/2778/9826/2977/113/4637/3778/135/775/5580/369/2767/5579/387/107/109/146/5894/5604 |
| hsa04514 | Cell adhesion molecules | 126 | 0.3874825 | 2.315650314 | 5.73E-08 | 1.25E-06 | 6.39E-07 | 3369 | tags=41%, list=20%, signal=33% | 83700/947/1003/57555/8506/5175/90952/84628/6383/5797/1462/83692/58494/1000/3684/80381/7412/3689/57502/57689/64115/4685/3383/920/1272/6404/5010/6614/9672/257194/4684/6401/3688/3384/5802/23705/54413/942/57863/7122/3683/6693/6403/2734/4267/3676/5819/3685/958/80380/923/94030 |
| hsa04072 | Phospholipase D signaling pathway | 135 | 0.3822171 | 2.303156628 | 7.61E-08 | 1.47E-06 | 7.49E-07 | 6456 | tags=63%, list=38%, signal=40% | 5159/22808/5155/10000/2149/2768/56034/23396/108/9265/8877/146850/8613/6464/399694/8611/5293/196883/115/27128/9162/8605/23533/10554/5156/2207/2846/5338/2534/2912/5330/5900/5154/2885/207/554/5737/185/111/5336/1759/9846/552/9266/9267/56895/208/10411/2778/56894/113/7249/6237/2475/5335/8525/3643/2185/387/5899/107/1902/109/5894/5604/8503/375/11069/80310/7248/6850/5898/1785/6654/382/8527/9170/6009/112/5290/1956/5594/56848/5294/8394 |
| hsa05205 | Proteoglycans in cancer | 195 | 0.3652041 | 2.280454712 | 1.09E-09 | 3.97E-08 | 2.03E-08 | 4896 | tags=49%, list=29%, signal=36% | 2260/1278/4313/1277/3678/3316/22808/2316/4478/6383/8321/7291/8325/117581/3339/10000/2335/1634/5600/4060/4318/3791/7040/8322/3693/857/7472/1514/5328/3059/7057/287/7483/3690/5293/2817/7078/3708/60/6608/7042/54776/2318/5566/3688/2535/3082/5777/2247/2885/207/3479/858/5336/208/4660/5962/9138/2002/6774/5329/7074/7097/3480/7409/7484/3685/967/5829/2099/7474/818/7482/867/2017/6237/2475/5335/369/6385/595/5170/7410/286/5579/7430/29102/6714/387/2317/6300/5894/5604/6548/8503/5879 |
| hsa05414 | Dilated cardiomyopathy | 85 | 0.4067484 | 2.279257824 | 3.65E-06 | 3.15E-05 | 1.61E-05 | 3313 | tags=44%, list=19%, signal=35% | 3678/22801/7169/7043/6444/7040/3693/108/781/3679/3690/196883/115/3908/5350/60/7042/3672/6442/5566/6546/3688/8515/3479/70/111/7171/4000/7168/3676/2778/55799/784/1605/488/3685/113 |
| hsa04670 | Leukocyte transendothelial migration | 102 | 0.3922955 | 2.275149295 | 8.32E-07 | 9.09E-06 | 4.64E-06 | 5558 | tags=57%, list=33%, signal=39% | 7070/83700/4313/10398/1003/5175/90952/4478/2771/83692/5600/87/4318/58494/6387/3684/7412/3689/3383/5293/6494/60/5010/7414/3688/5336/7122/3683/4688/653361/81/9564/2909/10411/4267/83593/3676/7409/1536/7852/5880/5829/1495/5335/7410/5579/7430/7408/2185/387/6300/8503/5879/1500/11069/1499/6093/50848 |
| hsa04666 | Fc gamma R-mediated phagocytosis | 95 | 0.3957698 | 2.265913633 | 1.47E-06 | 1.46E-05 | 7.45E-06 | 6823 | tags=69%, list=40%, signal=42% | 10000/23396/7454/8877/8613/2209/273/8611/3984/5293/3055/8605/2934/2212/2214/5338/207/3636/5336/9846/10095/2213/1073/208/10163/653361/10109/1793/7409/3985/5880/1072/50807/4082/55616/10093/1399/10810/5335/5580/7410/4651/5579/7408/8853/10096/1398/5894/5604/8503/5879/8936/6850/1785/382/10552/5788/5290/5594/57180/56848/65108/8394/10092/10097/5295 |
| hsa04933 | AGE-RAGE signaling pathway in diabetic complications | 98 | 0.3920174 | 2.262275433 | 1.91E-06 | 1.80E-05 | 9.20E-06 | 2515 | tags=37%, list=15%, signal=32% | 1284/1278/4313/1277/1281/7424/7056/1282/7043/10000/2335/5600/50507/7040/7412/6347/4772/3383/5293/5054/7042/5602/7423/6777/6401/5970/5292/4846/5330/207/185/5336/6776/208/84812/4790 |
| hsa05135 | Yersinia infection | 133 | 0.3724218 | 2.24570855 | 2.14E-07 | 3.34E-06 | 1.71E-06 | 7328 | tags=71%, list=43%, signal=41% | 3678/10000/2335/5600/23396/7454/6347/7456/4772/6196/3984/5293/920/60/5602/391/9844/8517/5609/5970/2212/3688/114548/58484/9815/207/7186/63916/10095/10454/3937/208/10163/4792/9138/4615/10109/4790/5585/148022/9564/3661/1793/3676/7409/5880/5829/3654/10093/1399/5335/7410/2533/2185/147179/6714/387/10096/6300/1398/5604/8503/5879/5606/8874/6093/4773/10458/23365/3551/382/3725/10552/23118/7189/7124/5290/5747/5594/8976/57180/8394/6885/10092/5605/10097/5295/5601/3586/644150/2776/1432/2932/71 |
| hsa04926 | Relaxin signaling pathway | 121 | 0.3732448 | 2.220955579 | 3.92E-07 | 5.59E-06 | 2.85E-06 | 2907 | tags=37%, list=17%, signal=31% | 1284/59/1278/4313/1277/1281/7424/1282/2771/10000/5600/4318/2775/7040/108/2791/6464/399694/409/5293/59345/196883/115/2793/10681/5602/54331/7423/5566/5609/5970/10488/4846/2788/5330/2885/207/111/2782/208/4792/4790/2790/408/2778 |
| hsa05100 | Bacterial invasion of epithelial cells | 75 | 0.400731 | 2.203649815 | 1.84E-05 | 0.0001287 | 6.57E-05 | 7030 | tags=73%, list=41%, signal=43% | 3678/2335/857/3611/6464/3059/399694/5293/60/391/9844/7414/23176/3688/23157/63916/858/1759/10095/10163/10109/9564/10801/1793/79658/5829/867/10093/1495/1399/2017/8218/1731/6714/387/10096/1398/8503/5879/55752/8936/1499/4735/1785/10552/5290/5747/10459/8976/57180/10092/10097/5295/55964/1213 |
| hsa04151 | PI3K-Akt signaling pathway | 303 | 0.339982 | 2.202855394 | 1.00E-10 | 4.69E-09 | 2.39E-09 | 4911 | tags=47%, list=29%, signal=34% | 1292/5159/1284/1291/2260/1278/1277/2324/3678/1293/22801/7424/5155/3910/7450/1435/1282/3913/7058/7010/9180/7148/3371/10000/2149/2335/3915/3791/1436/2323/2246/3693/56034/3563/2791/146850/3679/7059/7057/2255/2252/3690/63923/2321/5293/59345/3908/2793/1311/3912/3566/4803/5521/23533/3718/5228/10681/3667/3672/54331/5156/284/7423/8517/200186/2846/5970/4804/7060/10488/3688/4846/2788/4914/3082/8515/5154/2247/5649/2885/207/3479/7533/2782/208/3455/5526/285/4790/5585/57521/2790/7532/3676/6256/5528/3914/7097/3480/10018/3685/3560/9863/2057/7249/1388/842/94235/4908/3326/2250/3716/11140/2475/2997/598/7143/595/3918/5518/5170/627/9223/896/3381/4170/3643/3680/468/10319/55844/3559/1902/5894/5604/64764/8503/6696/5879/9470/3911 |
| hsa05418 | Fluid shear stress and atherosclerosis | 126 | 0.3683711 | 2.201437752 | 7.38E-07 | 8.35E-06 | 4.26E-06 | 3409 | tags=40%, list=20%, signal=33% | 4313/1003/5155/5175/7056/6383/10000/5327/5600/3554/4318/3791/59341/857/2949/1514/7412/6347/3383/3690/5293/4208/2817/60/90/5602/8517/5609/6401/5970/5598/4846/5154/207/7132/9181/858/3162/208/4688/653361/4790/1843/808/51588/801/4205/10365/3685/5880/8878 |
| hsa05146 | Amoebiasis | 87 | 0.3856399 | 2.175953737 | 1.35E-05 | 0.0001006 | 5.13E-05 | 1830 | tags=31%, list=11%, signal=28% | 1284/1278/1277/1281/3910/1282/3913/7043/2335/3554/87/3915/7040/338382/3684/3689/929/5293/3908/3912/7042/7414/5566/5970/5878/2774/5330 |
| hsa04142 | Lysosome | 126 | 0.3585557 | 2.142779457 | 2.50E-06 | 2.21E-05 | 1.13E-05 | 6550 | tags=64%, list=38%, signal=40% | 1513/1514/1522/4125/5660/8722/8907/57192/7805/23659/54/53/1509/1508/1200/1497/6556/1777/968/537/2629/3423/4864/4668/2760/3073/6609/10577/2588/162/8943/411/23062/5641/1519/527/3482/3916/4074/138050/8692/9516/9741/84572/967/9179/6448/535/2799/26088/23335/23431/5476/8218/23163/2548/51172/3988/2990/51606/5538/9374/27074/1201/9114/1075/245972/8120/4669/8905/410/950/3920/10239/4126/6272/22901/10947/427/10312/2717 |
| hsa04371 | Apelin signaling pathway | 128 | 0.3572263 | 2.140811004 | 1.98E-06 | 1.80E-05 | 9.20E-06 | 7301 | tags=67%, list=43%, signal=39% | 4854/59/22808/187/2771/1490/4638/10000/5327/108/2791/8877/146850/4209/59345/196883/115/5054/4208/2793/3708/23533/10681/54331/5566/6546/4899/4846/2788/5330/207/10014/185/111/2782/208/2790/808/801/4205/6261/10365/113/9759/7046/94235/6237/8678/2475/595/30849/6262/107/109/5894/5604/6548/4088/6696/643246/85366/1958/5571/4842/6547/112/182/5594/2770/814/100506013/56848/5294/81631/5565/440738/4635/3991/5605/8862/6263/2776/2783/3265/5581/5289 |
| hsa05200 | Pathways in cancer | 475 | 0.3182956 | 2.131609571 | 1.00E-10 | 4.69E-09 | 2.39E-09 | 6114 | tags=53%, list=36%, signal=35% | 5159/1284/4854/2260/4313/2324/4855/2735/7424/2736/5155/3910/2737/26508/1282/3913/83439/2771/8321/7043/9002/8325/10000/2149/862/2335/6688/2768/4318/3915/1436/2323/2246/7040/23493/8322/4616/3563/6387/108/2791/10235/2949/7472/4286/8643/5733/4791/51684/2255/23462/25780/7483/2252/5293/59345/196883/115/3908/2113/2793/6773/6608/25/3912/3566/1613/5915/861/7185/5914/3718/5228/1909/10681/7042/1857/5602/54331/5156/7423/8517/5566/1387/6777/2846/5970/1439/4853/3688/54567/367/5371/7709/5338/2034/5292/374654/1856/2788/1030/2535/5330/4914/3082/5900/5154/2247/51176/2885/207/115727/7187/7186/3479/185/111/5336/112398/2782/3162/6776/1487/624/208/4792/3455/9138/2033/5467/2002/4790/405/4149/9915/2790/808/8648/6774/801/6257/83593/6256/2778/3914/3480/9826/7852/7484/10018/3685/113/3560/5880/2099/7474/818/2057/6778/7046/842/623/613/94235/4851/3572/3601/3326/28514/7704/7482/867/2250/9817/1495/1399/3716/2475/598/10297/5335/10928/10587/369/595/1488/2767/3918/896/57449/5579/7048/10319/3460/387/1438/8454/3559/5899/2308/1398/107/1902/109/10023/5894/5604/11186/8503/4088/5879/3911/11040/1910/1647/8202/1441/3454/6772/3570/815/3728/3594/5727/1499/6093/3280/6667/8900/7175/5898/6654/23365/1027/3551/8312/3725/4041/3597/7170/7189/1612/9170/596/112/182/5290/1871/6789/5747 |
| hsa05145 | Toxoplasmosis | 96 | 0.3687965 | 2.121248696 | 2.81E-05 | 0.0001924 | 9.82E-05 | 7140 | tags=69%, list=42%, signal=40% | 3910/3913/2771/7043/10000/5600/3915/2775/7040/146850/3908/3912/23533/7042/3587/5602/8517/5970/3688/207/7132/7297/1234/10454/208/4792/23643/4615/4790/6774/3914/7097/958/3588/3654/3305/4793/842/3716/598/3918/5170/10319/3460/6300/3911/3306/5606/6772/3551/23118/7189/7124/596/5594/2770/5294/6885/5601/3586/3303/22798/331/3310/3949/1432 |
| hsa04014 | Ras signaling pathway | 207 | 0.3359168 | 2.112060222 | 1.65E-07 | 2.86E-06 | 1.46E-06 | 3098 | tags=36%, list=18%, signal=30% | 5159/2260/2324/22808/7424/5155/1435/7010/5322/10000/3791/1436/2323/2246/56034/2791/10235/6464/2255/23179/25780/2252/399694/2321/5293/59345/2113/2793/8605/25/4803/5228/8831/10681/64926/5602/54331/5156/284/7423/8517/5566/5970/4804/5338/5878/5063/2788/4914/3082/5900/5154/22821/2247/2885/207/115727/3479/5336/9846/2782/208/285/2002/4790/2790/808/801/83593/4303/7074/5924/27/3480 |
| hsa04662 | B cell receptor signaling pathway | 78 | 0.3783452 | 2.098105376 | 5.96E-05 | 0.0003432 | 0.0001751 | 6238 | tags=64%, list=36%, signal=41% | 10000/4772/25780/5293/10288/10859/11025/11006/4794/79168/971/8517/5970/975/695/5777/2885/207/3636/11024/5336/2213/208/4792/11027/4790/7409/5880/4793/353514/974/7410/5579/5894/5604/8503/5879/5533/4773/6850/6654/3551/23547/3725/10990/933/5532/5290/5594/118788 |
| hsa04064 | NF-kappa B signaling pathway | 98 | 0.3635685 | 2.098101204 | 3.62E-05 | 0.0002377 | 0.0001213 | 3216 | tags=42%, list=19%, signal=34% | 3554/4616/6387/5328/4791/7412/929/3383/5971/7185/4050/9020/8517/5970/23085/695/7132/7187/7186/8740/6366/5336/10454/4792/23643/7128/4615/128178/4790/10673/353376/148022/8837/23586/114609/4049/51588/1540/8737/958/142 |
| hsa04540 | Gap junction | 78 | 0.377056 | 2.09095621 | 6.30E-05 | 0.0003566 | 0.0001819 | 6284 | tags=62%, list=37%, signal=39% | 5159/84617/5155/2771/7846/2983/2982/2697/56034/108/3356/196883/115/3708/5156/5566/5598/5330/5592/5154/2885/111/1453/203068/2778/7280/2977/113/3357/2767/5579/7082/6714/107/1902/109/5894/5604/80310/347733/10381/6654/10376/112/1956/5594/2770/5607 |
| hsa00534 | Glycosaminoglycan biosynthesis - heparan sulfate / heparin | 22 | 0.5031248 | 2.06013268 | 0.0012098 | 0.0048991 | 0.0024999 | 4398 | tags=64%, list=26%, signal=47% | 9955/3340/9394/2134/8509/2137/2131/9953/64131/9956/64132/26229/2132/126792 |
| hsa05132 | Salmonella infection | 236 | 0.3239574 | 2.056100695 | 1.97E-07 | 3.23E-06 | 1.65E-06 | 7872 | tags=69%, list=46%, signal=38% | 84617/10398/57381/2316/83439/7846/10000/5600/338382/9265/23207/257364/929/5293/1639/27128/23339/60/112574/5420/10121/57617/5602/391/3071/9844/127829/2318/8517/10392/5609/5970/114548/5878/5063/58484/3798/51176/207/1778/7132/7186/23265/63916/9266/10095/10454/9267/208/4792/23643/4615/10109/4790/5585/55823/114609/29109/79443/203068/64601/23191/51429/4074/7097/1781/8655/8737/7280/113146/388/10333/3654/79026/9842/10540/7879/3326/64837/84516/10093/10810/6237/3831/140735/3800/5216/60412/387/10096/2317/1783/6300/27072/51209/5894/8717/5604/51143/5879/55770/79659/375/5606/5869/1499/347733/64746/10381/10120/5898/1785/5287/3551/382/3725/10552/23118/7189/5788/7124/596/10376/5290/65082/5594/3836/8976/57180/11035/1780/4671/8677/55845/5294/6885/10092/5605/7184/10097/10006/3320/837/8772/5601/84790/8743/1432/83658/3265/55207/5289/6500/71/5058/399/7100/5595/10382/55860/81873/197259/3569/302 |
| hsa04062 | Chemokine signaling pathway | 176 | 0.3325824 | 2.052261553 | 6.43E-07 | 7.73E-06 | 3.94E-06 | 6273 | tags=57%, list=37%, signal=37% | 2771/10000/6387/7454/108/2791/10235/57580/146850/2268/6464/6347/399694/409/5293/59345/3055/196883/115/2793/6773/2869/23533/1230/3718/10681/54331/9844/8517/5566/6777/1237/5970/2788/5330/1794/2885/207/6366/1234/111/5336/2782/208/4792/653361/4790/9564/2790/6774/408/7074/6376/7409/7852/2931/113/5880/2868/5829/58191/4793/6348/94235/156/1399/5335/5580/1524/56288/7410/5579/2185/6714/387/6354/1398/107/109/5894/5604/8503/5879/1236/6772/6355/2309/729230/6093/6654/3551/5906/6352/112/5290/6367/6358/5747/6368/5594/2770 |
| hsa04520 | Adherens junction | 71 | 0.3738707 | 2.045527581 | 0.0001384 | 0.0007543 | 0.0003849 | 7566 | tags=73%, list=44%, signal=41% | 2260/83439/5797/6591/87/7454/6615/60/7414/1387/2534/5777/51176/5770/10580/10163/2033/81/5819/3480/5880/7046/1495/10810/56288/3643/5795/7082/7048/6714/387/4088/5879/5787/1500/8936/1499/1460/10458/4008/1956/5594/5818/8976/8826/6885/1457/5792/71/4301/29119/5595 |
| hsa05217 | Basal cell carcinoma | 60 | 0.3848163 | 2.038195593 | 0.0002845 | 0.001414 | 0.0007215 | 1904 | tags=30%, list=11%, signal=27% | 2735/2736/2737/83439/8321/8325/8322/4616/7472/8643/51684/7483/6608/1857/374654/1856/2535/51176 |
| hsa04010 | MAPK signaling pathway | 269 | 0.3170368 | 2.034154839 | 1.13E-08 | 3.09E-07 | 1.58E-07 | 5943 | tags=52%, list=35%, signal=34% | 5159/2260/2324/22808/7424/5155/2316/1435/4215/7010/7043/8912/10000/5600/2768/3554/3791/1436/2323/2246/7040/4616/56034/781/7786/10235/4791/929/6722/4772/2255/25780/6196/2252/409/2321/4208/5971/9261/8605/9448/4803/5228/7042/9344/5602/5156/284/7423/9020/2318/8517/5566/5609/5970/4804/5598/1849/4914/3082/5154/2247/2885/207/7132/115727/7186/3479/1845/10454/208/4615/285/84867/1616/2002/4790/4149/1843/408/9479/55799/5924/3480/784/5880/8569/3654/3305/7046/4216/4908/11184/4296/2250/1399/6237/775/9064/369/627/8986/3643/51347/5579/782/468/7048/1847/5871/2317/6300/1398/4137/5894/8717/5604/5879/3315/773/1647/3306/5606/80310/5536/9693/5533/51776/51378/93589/6654/3551/5906/3725/2872/1326/23118/7189/7124 |
| hsa05163 | Human cytomegalovirus infection | 194 | 0.3231676 | 2.017031829 | 1.17E-06 | 1.20E-05 | 6.13E-06 | 6273 | tags=57%, list=37%, signal=37% | 2771/10000/5600/2768/3554/2775/6387/108/2791/4776/5733/6347/4772/3690/5293/59345/196883/115/2793/3708/1230/10681/3587/54331/5156/8517/5566/5970/10488/2788/5330/2885/207/7132/11214/7186/1234/111/2782/208/4792/9138/2002/4790/9564/2790/808/3661/811/6774/801/2778/9826/6376/7852/8737/3685/113/5880/5829/3588/7249/1388/6348/842/94235/1399/3716/2475/595/2767/5579/468/2185/6714/387/6300/1398/107/109/5894/8717/5604/64764/8503/5879/3570/340061/5533/1499/7248/6093/4773/6667/6654/23365/3551/6892/1385/7124/6009/6352/5532/112/5290/1871/5747/6891/1956/5594/2770 |
| hsa04722 | Neurotrophin signaling pathway | 116 | 0.3389254 | 1.999919053 | 5.65E-05 | 0.0003371 | 0.000172 | 7379 | tags=68%, list=43%, signal=39% | 4215/10000/5600/6464/2889/6196/399694/5293/10019/9261/25/4803/4794/3667/5602/5609/5970/4145/4804/5598/4914/2885/207/5336/208/4792/9500/4790/397/808/4916/801/57498/818/3654/4793/396/4908/1399/5335/5580/5664/5170/627/25970/468/387/6300/1398/5894/5604/8503/5879/815/2309/11108/6654/3551/5906/3725/3656/7189/596/5663/6272/5290/5594/5607/814/5605/10782/5295/5601/6655/1432/2932/3265/5781/11213 |
| hsa05131 | Shigellosis | 221 | 0.3152888 | 1.994734513 | 6.60E-07 | 7.73E-06 | 3.94E-06 | 7328 | tags=64%, list=43%, signal=37% | 10398/3678/7094/10000/5600/718/3554/87/3611/9265/3059/929/5293/3101/27128/23048/3708/60/5602/9844/25851/7414/8517/10318/10392/5970/23176/3688/114548/55072/58484/5330/23157/10670/207/7132/26100/9181/7186/63916/5336/3098/9266/10095/10454/9267/208/10163/4792/4615/84812/81/7316/10109/4790/9564/57521/3661/10801/1793/4303/7314/8945/8737/2931/5829/8878/4793/23291/10093/1399/2017/8678/2475/598/5335/5580/1729/30849/58528/84335/6714/5216/387/10096/8454/6300/2308/1398/823/64121/8717/8503/5879/643246/375/989/55752/340061/2309/8936/6093/4735/3551/55054/382/3725/10552/23118/7189/7124/51619/199746/596/6352/824/5290/5747/1956/5594/8976/57180/4671/81631/440738/10241/6885/10092/10097/837/5295/5601/7321/55964/10325/1432/2932/81858/5581/5289/6500/71 |
| hsa04620 | Toll-like receptor signaling pathway | 88 | 0.3525223 | 1.991549795 | 0.0001403 | 0.0007543 | 0.0003849 | 6825 | tags=66%, list=40%, signal=40% | 1513/10000/5600/929/5293/5602/8517/5609/5970/207/7187/942/10454/208/4792/23643/3455/4615/3665/4790/353376/148022/54472/114609/3661/7097/8737/958/10333/3663/3654/6348/51284/6300/5604/8503/6696/5879/9641/5606/3454/6772/3551/3725/1326/23118/7189/7124/6352/5290/5594/7096/941/6885/5605/8772/5295/5601 |
| hsa05152 | Tuberculosis | 142 | 0.328589 | 1.987757936 | 1.61E-05 | 0.0001145 | 5.84E-05 | 6843 | tags=62%, list=40%, signal=37% | 9902/4046/7043/10000/5600/718/7040/3684/8877/3689/929/2209/1509/3687/7042/3587/5602/2207/11151/1387/5970/2212/2214/537/5878/207/7132/2213/208/4615/2033/4790/114609/808/801/527/5993/3916/7097/8844/1263/10333/4802/3588/972/818/3654/535/842/7879/4801/64581/3716/4800/6714/3460/387/6300/5894/8717/51606/4360/8625/5869/6772/815/9114/5533/245972/7421/26253/6850/23365/3656/3920/1385/7189/7124/596/5532/5594/7096/56848/10312/8772/5601/1051/3586 |
| hsa05140 | Leishmaniasis | 57 | 0.3787654 | 1.985915064 | 0.0010206 | 0.0042374 | 0.0021622 | 6566 | tags=65%, list=38%, signal=40% | 7043/5600/718/7040/3684/3689/2209/7042/5970/2212/3688/2214/5777/10454/4792/4688/4615/653361/2002/4790/3676/7097/1536/3654/4793/3716/5579/3460/6300/6772/3725/23118/7189/7124/5594/65108/6885 |
| hsa05032 | Morphine addiction | 74 | 0.3617752 | 1.9858832 | 0.0002373 | 0.001231 | 0.0006282 | 3322 | tags=39%, list=19%, signal=32% | 5138/2771/5153/2775/108/2791/5136/409/59345/196883/134/115/2793/2563/2869/10681/54331/5566/2788/3762/5141/111/2782/27115/2790/408/2778/113/2868 |
| hsa05169 | Epstein-Barr virus infection | 163 | 0.3242847 | 1.985367362 | 9.66E-06 | 7.54E-05 | 3.85E-05 | 7207 | tags=65%, list=42%, signal=38% | 7431/10000/5600/4616/953/4791/3383/5293/5971/6773/4794/3718/5602/9612/9020/8517/5609/5970/695/207/7297/7187/7186/5336/3683/864/10454/208/4792/3455/7128/4615/5708/3665/4790/5710/23586/3661/811/6774/7097/25942/5713/10018/8737/958/5705/79685/5700/3654/4793/842/5704/3716/4940/595/11047/5702/896/5707/5701/9541/6300/4939/8717/8503/9636/5879/5717/9641/1647/22938/5606/3454/6772/3280/8900/6850/10379/1027/3551/6892/3725/8819/23118/7189/7124/596/57506/5290/916/1871/7979/6891/6885/3516/2923/8772/5295/5601/5610/4938/9861/5709/1432/7874 |
| hsa05167 | Kaposi sarcoma-associated herpesvirus infection | 164 | 0.3234714 | 1.982140315 | 9.24E-06 | 7.39E-05 | 3.77E-05 | 6849 | tags=61%, list=40%, signal=37% | 5155/83439/10000/5600/718/2791/4776/57580/146850/4772/3383/5293/59345/3055/9976/2793/3708/6773/9261/23533/1230/10681/5602/54331/8517/1387/5609/1237/5970/2788/2247/51176/207/7132/7297/7187/7186/1234/942/5336/2782/208/4792/3455/2033/285/3665/7316/4790/148022/2790/808/3661/6774/1827/801/7314/842/94235/3572/3716/8678/2475/5335/595/6714/7538/6300/5894/8717/5604/8503/5879/9641/643246/3454/6772/5533/1499/4773/6850/10379/3551/64422/3725/1385/5532/100507436/5290/1871/5594/3091/5294/81631/440738/5605/8772/5295/5601/5610 |
| hsa05221 | Acute myeloid leukemia | 64 | 0.3689766 | 1.980738524 | 0.0007013 | 0.0030267 | 0.0015444 | 6959 | tags=67%, list=41%, signal=40% | 83439/10000/862/6688/1436/3684/929/2209/5293/861/5914/8517/6777/5970/5371/5292/51176/2885/207/6776/208/5467/4790/6774/7704/2475/369/595/5894/5604/8503/11040/3728/8900/6654/3551/597/5290/5594/5605/8864/5295/6655 |
| hsa05165 | Human papillomavirus infection | 291 | 0.3055719 | 1.973136984 | 6.13E-08 | 1.26E-06 | 6.42E-07 | 6227 | tags=52%, list=36%, signal=34% | 1292/5159/1284/1291/4854/1278/1277/4855/3678/1293/22801/3910/26508/7450/1282/3913/83439/7058/8321/8325/7148/3371/10000/2335/3915/23493/8322/3693/7472/3679/7059/7057/23462/7483/3690/63923/4242/84552/5293/3908/6773/1311/3912/5521/3672/1857/4600/27175/8517/5566/1387/5970/4853/3996/7060/10488/3688/537/1856/2535/8515/5649/2885/207/7132/7297/1740/7187/1108/9794/208/3455/5526/4799/2033/534/4790/148022/3661/527/3676/2778/5528/3914/10474/7484/3685/5829/23352/5700/7474/7249/535/4851/7482/4599/8992/3716/2475/529/7143/10297/595/3918/5518/56288/9223/896/3381/3680/10134/6908/55534/10319/55844/2308/5894/8717/5604/51606/64764/8503/9636/6696/3911/9641/84441/3454/6772/9114/1499/7248/245972/3280/8900/528/10379/6654/1027/3551/526/8312/1385/7124/6009/5663/182/5290/5747/1956/5594/51382 |
| hsa04740 | Olfactory transduction | 40 | 0.4036569 | 1.968660427 | 0.0029329 | 0.0104971 | 0.0053563 | 5526 | tags=60%, list=32%, signal=41% | 5138/5153/5136/409/5997/5566/81285/6546/2774/2788/5592/2782/808/408/801/818/156/123041/1261/143503/109/57101/815/83988 |
| hsa05130 | Pathogenic Escherichia coli infection | 178 | 0.3183056 | 1.967666686 | 6.19E-06 | 5.20E-05 | 2.66E-05 | 8153 | tags=70%, list=48%, signal=37% | 84617/7846/2149/5600/2768/3554/4542/9265/4628/3059/4629/7456/4644/4627/27128/60/25/5010/5602/3071/8517/2846/5970/2212/64005/3688/114548/2534/5063/5777/4641/7132/9181/7186/7122/9266/10095/10454/9267/10163/4792/9138/4615/10109/4790/114609/203068/8440/23191/9826/8737/7280/3654/4793/9632/842/4691/10093/4643/2017/10810/10061/4651/7430/7082/147179/6714/387/10096/6300/1902/8717/5879/375/8936/347733/6093/10381/10458/23365/3551/9871/382/3725/10552/23118/7189/7124/9170/5861/10376/5594/8976/57180/4671/55845/4690/6885/10092/5062/10097/10006/837/79784/8772/5601/84790/8743/644150/1432/5781/71/55971/5058/4430/7100/5595/10382/81873/4642/3569/1364/7009/10972/5603 |
| hsa04928 | Parathyroid hormone synthesis, secretion and action | 96 | 0.3417076 | 1.965438088 | 0.0002582 | 0.0013031 | 0.0006649 | 6273 | tags=58%, list=37%, signal=37% | 2260/4323/2771/9935/2768/108/4209/5744/409/196883/5745/115/4208/3708/4325/860/5566/10488/5338/2625/5330/5141/11214/111/9138/408/6257/6256/2778/4205/9826/113/1388/4326/369/2767/5579/468/387/107/109/5894/5604/64764/1958/7421/9365/6667/4322/1385/4041/596/112/1956/5594/2770 |
| hsa04962 | Vasopressin-regulated water reabsorption | 40 | 0.4027352 | 1.96416491 | 0.0031629 | 0.0111553 | 0.0056922 | 6316 | tags=68%, list=37%, signal=43% | 1639/115/6810/5566/10488/6844/5878/1778/554/397/2778/1781/8655/9230/396/10540/84516/140735/1783/109/64764/51143/79659/5869/1385/112/1780 |
| hsa04141 | Protein processing in endoplasmic reticulum | 162 | 0.3187398 | 1.951165792 | 1.23E-05 | 9.38E-05 | 4.79E-05 | 10148 | tags=88%, list=59%, signal=36% | 1410/7991/57134/3300/5602/7415/5609/84447/10956/29927/55666/7186/54788/22872/91445/8720/51035/5589/11253/811/23193/85479/7841/3301/56893/3309/23645/10525/55968/6238/1388/3305/80700/440275/9632/80331/7353/3326/81567/64374/29978/5886/55829/10961/3703/133022/10134/573/468/10113/8454/55741/10130/823/6746/9709/6400/3306/5034/2081/10484/267/64215/10277/5887/51726/9373/6185/9601/6184/9871/6396/7327/22926/51619/596/824/11231/7095/10952/10970/7184/23480/9451/56681/27102/100506742/2923/3320/5601/56886/5610/7321/118424/3303/79139/3310/10483/10960/7326/1650/6500/51360/9532/51009/27248/165324/821/10299/29979/51465/10294/7466/9695/10427/56605/6048/581/23471/7322/4780/23190/4217/1603/7323/3312/10802/4287/9978/578/10905/3304/1649/6748/5611/6747/4121/5599/10808/1965/26270/54431 |
| hsa04020 | Calcium signaling pathway | 204 | 0.31097 | 1.950609014 | 7.02E-06 | 5.75E-05 | 2.94E-05 | 4086 | tags=39%, list=24%, signal=30% | 5159/2260/2324/6915/7424/5155/8912/4638/5153/2149/3791/2246/56034/108/8877/5733/3707/3356/2255/57192/80228/5136/2252/493/2321/5027/196883/115/5350/3708/4803/57172/1909/8536/5156/7423/5566/6546/3274/2774/4846/5330/4914/3082/5154/2247/6786/5737/185/5336/552/624/80271/3270/5724/219931/84812/808/4916/801/845/2778/3269/5260/6261/7852/488/113/93129/818/623/3357/10800/53373/2250/135/775/844/5335 |
| hsa05161 | Hepatitis B | 144 | 0.3217306 | 1.950064533 | 3.34E-05 | 0.0002235 | 0.0001141 | 6959 | tags=62%, list=41%, signal=37% | 7043/3339/10000/5600/4318/1959/7040/4776/4772/5293/6773/3718/7042/5602/8517/1387/6777/5609/5970/10488/537/2885/207/7297/7187/6776/10454/208/4792/4615/2033/2002/3665/4790/353376/148022/23586/114609/3661/6774/1642/7097/3654/1388/6778/7046/842/64135/3716/369/5579/468/7048/2185/6714/6300/5894/5604/64764/8503/4088/9641/5606/3454/6772/4773/8900/6654/3551/3725/1385/23118/7189/7124/1960/596/57506/5290/1871/1654/5594/6885/5605/100506742/8772/5295/5601/7529/1386/6655 |
| hsa04935 | Growth hormone synthesis, secretion and action | 104 | 0.3351309 | 1.946033127 | 0.0002215 | 0.0011718 | 0.0005979 | 7249 | tags=65%, list=42%, signal=38% | 2771/10000/5600/3486/108/6464/9021/399694/5293/196883/115/3708/3667/5602/5566/1387/6777/10488/5330/2885/207/3479/111/5336/6776/6752/208/2033/9564/6774/2778/113/1388/1399/2475/775/5335/2767/5579/468/6300/1398/107/109/5894/5604/64764/8503/2690/5606/6772/3726/6654/1385/112/5290/5747/5594/2770/5605/5295/5601/1386/6655/2776/1432/2932/3265 |
| hsa04144 | Endocytosis | 234 | 0.3067562 | 1.944270008 | 6.34E-07 | 7.73E-06 | 3.94E-06 | 9267 | tags=78%, list=54%, signal=36% | 30846/30845/11031/440073/5662/23396/857/9265/9922/23111/7456/273/409/84552/116985/27128/2869/26056/1173/5156/22905/11021/10938/6455/5371/8729/5338/160/5878/55048/58513/9815/3798/161/116988/9727/64744/1234/858/1759/9266/10095/9267/112936/80230/832/57154/10109/253725/408/3482/9744/84364/6642/4218/3480/27183/7852/3560/2868/29934/89853/64145/387680/9230/79643/3305/50807/7046/377/55616/7879/55738/11267/23096/116987/867/10093/156/9798/30844/8218/3800/100287171/56288/29924/9146/91782/7048/128866/147179/6714/10193/387/8853/10096/3559/27243/51652/57132/9135/5119/4088/11311/1175/6643/375/26286/3306/28964/5869/1601/2350/868/51324/60682/9372/7251/1785/382/10552/8724/7189/93343/1956/118813/8976/57180/64411/8394/10092/9897/10097/51510/10617/23550/2870/51100/378/3303/1213/3310/644150/30011/3949/163/10015/3265/26119/29082/64750/79720/10890/81873/51160/8027/157/84440/381/1212/1211/9101/22841/5590/57403/116986/116983/80223/3561/51534/10564/9525/3312/4734/58533/155382/5868/2060/23362/3799/998/119016 |
| hsa03250 | Viral life cycle - HIV-1 | 62 | 0.36322 | 1.935967033 | 0.0009428 | 0.0039646 | 0.002023 | 5969 | tags=61%, list=35%, signal=40% | 4130/9638/4131/8178/920/23299/6829/25939/4600/1025/1387/5045/1234/4298/2033/55201/79869/27183/7852/79643/23534/6598/4599/684/5300/7469/9146/128866/200316/10955/25920/85363/60489/636/7251/140564/200315/11052 |
| hsa05202 | Transcriptional misregulation in cancer | 159 | 0.3161582 | 1.935207242 | 3.73E-05 | 0.00024 | 0.0001225 | 5941 | tags=51%, list=35%, signal=34% | 4066/2078/4211/4094/2313/862/5327/6688/6935/4318/1436/4005/3486/4616/3684/4286/5328/7030/5218/929/2209/2321/5090/4208/8861/861/7185/5914/3087/860/604/1025/5970/4804/5371/7709/4914/5154/3479/942/4298/2521/2130/4790/4149/9915/6257/6256/6760/25942/2892/3480/958/3560/8148/5546/2115/7704/4086/51804/598/4297/4221/6929/8013/7048/2308/1031/5087/1647/1655/2120/3728/8464/6667/8900/1027/6495/171558/26471/597 |
| hsa01522 | Endocrine resistance | 91 | 0.3402967 | 1.933938504 | 0.0002402 | 0.001231 | 0.0006282 | 7249 | tags=66%, list=42%, signal=38% | 4854/4313/4855/10000/5600/4318/108/6464/399694/5293/196883/115/5602/5566/4853/54567/2885/207/3479/111/208/2778/3480/113/2099/4851/28514/2475/369/595/6714/6300/107/109/5894/1031/5604/8503/8202/10498/5469/6667/6654/1027/3725/596/112/182/5290/1871/5747/1956/5594/5605/5295/5601/9611/6655/1432/3265 |
| hsa04140 | Autophagy - animal | 137 | 0.3195849 | 1.930798271 | 9.53E-05 | 0.0005299 | 0.0002704 | 8719 | tags=76%, list=51%, signal=38% | 22808/10000/338382/1514/53349/5293/8408/3708/1509/1508/1613/23710/3667/5602/5566/9776/10670/207/26100/79065/9711/55626/208/23130/10645/58476/8837/57521/7405/140775/3916/4218/60673/9342/3480/64419/8878/10533/11337/7249/440275/7879/8897/6237/8678/2475/598/5580/9706/30849/5170/23192/58528/84335/64121/5894/5604/8503/643246/29982/2081/7248/55054/64422/3920/55102/7189/1612/5861/596/6009/54541/5290/5594/3091/81631/440738/6885/5605/9451/5295/5601/8673/51100/10325/11345/3476/3265/5289/5595/115201/55255/5516/22863/55578/55062/572/9821/6198/10010/29110/5728/6794/55014 |
| hsa04360 | Axon guidance | 173 | 0.3125777 | 1.925312622 | 1.58E-05 | 0.0001145 | 5.84E-05 | 6700 | tags=58%, list=39%, signal=36% | 10398/6586/91653/10501/84628/219699/2771/56920/56963/8829/1949/91584/2242/9353/6387/3611/4776/10500/5998/57689/7223/84552/3984/5293/54910/7220/54437/54434/2042/6608/25/9423/23380/8482/7225/5361/10154/22885/3688/2534/5063/6091/5336/1073/7222/8440/2050/8633/7852/23654/64221/3985/5880/7474/818/1072/10507/57522/5362/6237/659/5335/5170/56288/658/6714/223117/387/90249/2051/5894/8503/64218/5879/5365/151449/55558/815/54961/5533/5727/6093/4773/23365/85464/10505/55740/5532/5290/5747/5594/2770/64101/1947/4690/6405/1808/5062/10298/1020 |
| hsa04390 | Hippo signaling pathway | 146 | 0.3163968 | 1.920151625 | 5.81E-05 | 0.0003403 | 0.0001736 | 4926 | tags=45%, list=29%, signal=32% | 2736/83439/1742/8321/7043/1490/6591/126374/8325/122786/25937/2246/7040/8322/26524/7472/3689/7483/84552/5054/60/7005/5521/7042/1857/353500/4092/3996/392255/1856/2535/51176/9231/1740/7533/84962/1453/1454/8200/654/7532/8945/7484/7474/7046/23291/4086/7482/1495/7159/659/10297/595/5518/56288/896/658/23286/7048/7003/55844/11186/4088/656/60485 |
| hsa04022 | cGMP-PKG signaling pathway | 147 | 0.3155752 | 1.914174795 | 4.52E-05 | 0.0002796 | 0.0001427 | 4814 | tags=44%, list=28%, signal=32% | 10398/4881/5138/10335/2771/2983/2982/4638/10000/3779/2768/3764/4882/108/4776/146850/4209/6722/4772/482/493/196883/134/115/4208/5350/3708/23533/1909/3667/7225/5997/6546/10488/4846/5330/5592/207/185/111/624/208/808/801/4205/488/2977/113/477/1388/8654/3778/2969/775/2767/3643/468/7408/387/107/109/146/5894/5604/64764 |
| hsa05206 | MicroRNAs in cancer | 160 | 0.3115108 | 1.907618356 | 4.52E-05 | 0.0002796 | 0.0001427 | 4933 | tags=44%, list=29%, signal=32% | 5159/7431/4854/4855/3678/5155/7148/3371/23414/8434/6935/4318/9839/5328/6464/7057/399694/3690/63923/5293/7078/25/4325/90427/3667/7042/5156/1387/4853/5598/5292/5154/2885/599/10014/1545/5336/3162/5962/2033/4790/57521/1788/6774/7168/10018/6624/9759/4082/4851/1786/6659/1399/2475/659/7143/10297/5335/595/4363/4170/5579/7430/387/1398/5894/5604/11186/8503/3190/7329 |
| hsa05215 | Prostate cancer | 91 | 0.3333718 | 1.89458365 | 0.0003546 | 0.0017103 | 0.0008727 | 7249 | tags=64%, list=42%, signal=37% | 5159/2260/5155/83439/2078/10000/5327/6935/4318/56034/5328/5293/5156/8517/1387/5970/10488/367/5154/51176/2885/207/3479/208/4792/2033/4790/3480/842/3326/2475/369/595/5170/468/2308/5894/5604/64764/8503/80310/1499/6654/1027/3551/1385/596/5290/1871/1956/5594/5605/7184/3320/5295/6655/2932/3265 |
| hsa04130 | SNARE interactions in vesicular transport | 33 | 0.4100292 | 1.894539559 | 0.0036838 | 0.0127188 | 0.00649 | 8719 | tags=88%, list=51%, signal=43% | 10791/6810/10652/6844/6811/51272/9570/8676/2054/9342/10228/55850/10490/9482/9554/8773/112755/6845/6843/9527/8673/53407/6804/8417/143187/8674/9341/8675/55014 |
| hsa04625 | C-type lectin receptor signaling pathway | 97 | 0.3276178 | 1.887577323 | 0.0005638 | 0.0025334 | 0.0012927 | 8254 | tags=72%, list=48%, signal=38% | 22808/4046/10000/5600/1959/4776/4791/4772/5293/3708/5971/6773/9261/5602/2207/9020/8517/5970/114548/207/5336/208/4792/4790/808/801/1540/8844/1263/64581/6237/5580/6714/387/6300/5894/8503/9641/6772/5533/26253/4773/868/6850/10379/23365/3551/3725/7124/1960/5532/5290/6367/5594/5295/5601/3586/1432/3265/5781/5058/5595/64170/602/30835/3569/3709/5603/5534/3710 |
| hsa05323 | Rheumatoid arthritis | 67 | 0.3482456 | 1.883471894 | 0.0026079 | 0.0096207 | 0.0049092 | 8272 | tags=72%, list=48%, signal=37% | 1435/7010/7043/1513/7040/6387/1514/3689/6347/3383/2321/54/7042/4050/284/537/942/3683/534/10673/527/7097/535/6348/8992/529/51606/9114/1493/245972/528/526/3725/7124/6352/940/51382/941/10312/9296/9550/7422/155066/3569/90423/8741/8600/523 |
| hsa05142 | Chagas disease | 97 | 0.3263129 | 1.880059006 | 0.0006188 | 0.002743 | 0.0013997 | 6843 | tags=61%, list=40%, signal=37% | 2771/7043/10000/5600/718/2775/7040/6347/5293/5054/712/5521/714/7042/5602/8517/5970/2774/5330/207/7132/713/624/208/4792/4615/4790/148022/8837/811/2778/7097/10333/3654/6348/7046/2767/5518/7048/3460/55844/6300/107/8503/9630/3551/3725/7189/7124/6352/5290/916/5594/2770/1636/8772/5295/5601/3586 |
| hsa04921 | Oxytocin signaling pathway | 136 | 0.3111644 | 1.87831418 | 0.0003122 | 0.0015281 | 0.0007798 | 7413 | tags=62%, list=43%, signal=35% | 10398/4881/2771/2983/2982/4638/2775/4882/108/781/4776/146850/4772/196883/115/4208/3708/60/8605/57172/23533/54776/8536/5997/5566/5598/4846/5330/3762/111/4660/10645/2002/808/140465/1827/801/2778/55799/6261/784/2977/113/4637/818/29904/775/595/5579/782/6714/387/6262/107/109/5894/5604/85366/815/5571/5533/6093/4773/93589/3725/5532/112/1956/5594/2770/5607/814/5294/5565/5021/5605/6263/4659/2776/1938/57118/3265/71/340156 |
| hsa05410 | Hypertrophic cardiomyopathy | 81 | 0.3359726 | 1.872329353 | 0.0012884 | 0.0051535 | 0.0026297 | 3204 | tags=36%, list=19%, signal=29% | 3678/22801/7169/7043/6444/7040/3693/781/3679/3690/3908/60/7042/3672/6442/6546/3688/8515/3479/70/7171/4000/7168/3676/55799/784/1605/488/3685 |
| hsa04659 | Th17 cell differentiation | 86 | 0.3325131 | 1.87077248 | 0.0008154 | 0.0034736 | 0.0017725 | 6212 | tags=59%, list=36%, signal=38% | 5600/3554/7040/50943/4772/920/3566/4794/861/5914/3718/5602/8517/6777/5970/50615/2625/7297/6776/4792/4790/6774/6257/6256/3560/4793/6778/7046/3572/3326/3716/2475/6095/5335/7048/3460/6300/3559/4088/9466/6772/3570/3594/5533/4773/3551/10148/3725/5532/916/5594 |
| hsa05120 | Epithelial cell signaling in Helicobacter pylori infection | 65 | 0.3478391 | 1.869029242 | 0.0026105 | 0.0096207 | 0.0049092 | 9255 | tags=83%, list=54%, signal=38% | 83700/5600/58494/5602/9020/8517/10392/5970/537/5336/4792/534/4790/1445/527/535/8992/529/5335/6868/7082/6714/6300/51606/5879/5803/28964/9114/245972/50848/528/3551/526/3725/6352/1956/51382/10312/5601/9296/9550/1432/5781/5058/155066/90423/5603/523/102/4067/6416/1147/23545/998 |
| hsa04630 | JAK-STAT signaling pathway | 128 | 0.310429 | 1.860360991 | 0.0004356 | 0.0020315 | 0.0010366 | 6193 | tags=55%, list=36%, signal=35% | 5159/5155/9180/10000/3563/9021/2273/5293/6773/3566/3718/3587/5156/7066/1387/6777/1439/1489/50615/5292/5777/5154/2885/207/7297/10401/6776/208/3455/2033/3977/6774/51588/3560/316/3588/2057/6778/3572/3601/3716/2475/598/595/9655/3590/896/4170/3460/1438/3559/5894/8503/2670/1441/2690/9466/3454/6772/8554/3570/3594/3581/10379/6654/3597/64109/596/5290/1956 |
| hsa05416 | Viral myocarditis | 37 | 0.3875282 | 1.855857076 | 0.0061707 | 0.0185686 | 0.009475 | 7879 | tags=76%, list=46%, signal=41% | 6444/857/3689/3383/3908/60/25/6442/2534/942/1981/3683/27/1605/958/5880/842/595/5879/8672/6443/940/941/1756/5551/71/6445/1982 |
| hsa04392 | Hippo signaling pathway - multiple species | 28 | 0.414668 | 1.830403428 | 0.0073393 | 0.0213035 | 0.0108705 | 2432 | tags=39%, list=14%, signal=34% | 8642/126374/122786/25937/26524/9770/83937/79633/7005/84962/1454 |
| hsa04211 | Longevity regulating pathway | 81 | 0.3272392 | 1.823659256 | 0.0021051 | 0.008122 | 0.0041444 | 6938 | tags=63%, list=41%, signal=38% | 10000/108/5293/196883/8408/115/3667/5566/5970/10488/9776/207/3479/111/208/10645/79813/4790/57521/10919/60673/3480/113/7249/1388/6648/2475/3643/84335/468/2308/107/109/64764/8503/9470/2309/5571/7248/9365/1385/6009/112/5290/83667/814/51094/5565/79602/5295/1386 |
| hsa05170 | Human immunodeficiency virus 1 infection | 183 | 0.292484 | 1.815653044 | 5.36E-05 | 0.0003256 | 0.0001661 | 9513 | tags=78%, list=56%, signal=35% | 2771/10000/5600/2775/2791/4776/8907/4772/3984/5293/59345/920/2793/3708/25939/10681/5602/54331/8517/5609/5970/5063/2788/207/7132/7186/1234/5336/2782/10454/1073/208/4792/4615/162/7133/4790/2790/808/3661/811/801/1642/7097/8945/7852/8737/3985/5880/5829/3654/1072/842/94235/23291/9730/684/1399/2475/598/5335/2767/5579/2185/8454/200316/6300/1398/9616/5894/8717/5604/8503/85363/5879/60489/5606/340061/5533/4773/8905/3551/6892/140564/3725/200315/23118/7189/7124/596/5532/5290/916/5747/6891/5594/2770/6885/5062/5605/10298/2923/8772/5295/5601/2776/1432/2783/3265/6500/8906/919/5058/567/5595/164668/3709/27350/5603/5534/3710/572/2773/581/6198/6890/29110/56924/8450/472/164/4775/1147/841/5578/9978/578/2784/8065/5530/6199/8451 |
| hsa00603 | Glycosphingolipid biosynthesis - globo and isoglobo series | 13 | 0.5118504 | 1.814457826 | 0.0113065 | 0.0303979 | 0.0155111 | 3033 | tags=54%, list=18%, signal=44% | 53947/6489/6483/26301/4668/3073/6482 |
| hsa05162 | Measles | 120 | 0.3039062 | 1.806720669 | 0.0006915 | 0.0030241 | 0.0015431 | 7043 | tags=62%, list=41%, signal=37% | 4478/10000/5293/6773/3718/5602/4600/8517/6777/5970/207/103/7297/7187/6776/2213/208/4792/3455/7128/4615/3665/4790/23586/3661/6774/7097/3560/3654/3305/4793/440275/842/64135/4599/51284/3716/598/4940/595/896/3559/4939/51209/8717/8503/9641/3306/3454/6772/1460/868/10379/1027/3551/3725/23118/7189/596/57506/5290/916/940/6885/9451/6504/27102/8772/5295/5601/5610/4938/3303/1457/3310 |
| hsa05412 | Arrhythmogenic right ventricular cardiomyopathy | 70 | 0.3300319 | 1.805349494 | 0.0034569 | 0.0120622 | 0.006155 | 3204 | tags=36%, list=19%, signal=29% | 3678/22801/83439/6444/2697/3693/781/1000/3679/3690/3908/60/3672/6442/6546/3688/8515/51176/4000/3676/55799/784/1605/488/3685 |
| hsa04658 | Th1 and Th2 cell differentiation | 74 | 0.3288209 | 1.804987937 | 0.0014797 | 0.0058475 | 0.0029838 | 6212 | tags=58%, list=36%, signal=37% | 4854/4094/5600/4772/920/3566/4794/3718/5602/8517/6777/5970/4853/54567/2625/7297/6776/864/9794/4792/4790/3560/4793/6778/4851/28514/3716/5335/55534/3460/6300/3559/84441/6772/3594/5533/4773/3551/3725/5532/182/916/5594 |
| hsa05211 | Renal cell carcinoma | 67 | 0.3330145 | 1.801095251 | 0.0058448 | 0.0177752 | 0.0090701 | 7621 | tags=69%, list=45%, signal=38% | 5155/7043/10000/7040/7030/2889/5293/2113/7042/1387/2034/5063/3082/2885/207/201163/112398/208/2033/405/9915/5546/1399/369/1398/5894/5604/8503/5879/6654/5906/3725/5290/5594/3091/5062/5605/10298/5295/6655/8453/3265/5781/5058/5595/7422 |
| hsa04923 | Regulation of lipolysis in adipocytes | 50 | 0.3510614 | 1.797354295 | 0.0069927 | 0.0204786 | 0.0104496 | 3313 | tags=38%, list=19%, signal=31% | 4881/2771/10000/108/5733/5293/196883/134/115/5742/3667/5566/5592/207/111/208/2778/2167/113 |
| hsa04713 | Circadian entrainment | 89 | 0.317254 | 1.795096367 | 0.0024405 | 0.0092008 | 0.0046949 | 3633 | tags=37%, list=21%, signal=29% | 2771/2983/2982/8912/2775/108/2791/59345/196883/115/2793/3708/10681/54331/5187/5566/2788/5330/5592/117/3762/111/2782/2790/808/801/2778/2892/6261/2977/113/818/94235 |
| hsa04621 | NOD-like receptor signaling pathway | 146 | 0.2918472 | 1.771165265 | 0.0005266 | 0.0023991 | 0.0012242 | 8338 | tags=69%, list=49%, signal=36% | 51393/84168/5600/22861/6347/5027/3708/1508/9051/6773/23710/5602/8517/10392/5970/114548/55072/58484/5330/7297/7187/7186/22900/91662/10454/24145/4792/3455/7128/4615/79671/3665/4790/5585/148022/3661/9927/1536/8737/3428/11337/4793/3326/118429/2634/3716/598/7158/4940/5580/387/6300/4939/9641/56919/643246/3454/6772/340061/2633/10379/3551/55054/84674/3725/23118/7189/7124/596/6352/57506/10628/5594/11035/4671/81631/440738/115362/6885/100506742/3320/837/8772/5601/4938/331/1432/11345/81858/6041/5595/64170/3569/3709/79184/5603/10616/3710/8767/115361/7531 |
| hsa01521 | EGFR tyrosine kinase inhibitor resistance | 75 | 0.3205651 | 1.76281171 | 0.0054347 | 0.0173065 | 0.008831 | 9514 | tags=81%, list=56%, signal=36% | 5159/5155/558/10000/3791/56034/6464/399694/5293/5156/3082/5154/2247/2885/207/3479/5336/208/6774/3480/10018/3716/2475/598/5335/369/5579/6714/5894/5604/8503/9470/3570/2309/80310/6654/596/5290/1956/5594/5605/5295/6655/2065/2932/3265/5595/7422/3569/572/673/4763/581/6198/2064/5728/5578/2621/2549/6199/25759 |
| hsa05224 | Breast cancer | 133 | 0.2921853 | 1.761881453 | 0.0010769 | 0.0044152 | 0.002253 | 2009 | tags=25%, list=12%, signal=22% | 4854/2260/2324/4855/26508/83439/8321/8325/10000/2246/23493/8322/4616/7472/4791/6464/2255/23462/7483/2252/399694/5293/5241/1857/4853/54567/1856/2535/2247/51176/2885/207/3479 |
| hsa03040 | Spliceosome | 127 | 0.2939521 | 1.758902151 | 0.0016676 | 0.0065116 | 0.0033227 | 10106 | tags=85%, list=59%, signal=35% | 9092/10291/4670/8449/10594/9785/10523/2521/23020/5093/10992/22985/1659/9416/153527/24148/9939/10262/26121/11338/3305/11325/6625/6429/56949/27339/56259/84991/8683/6100/57461/6634/151903/3192/9129/23450/144983/9343/3190/3306/22938/8175/25766/25949/51729/1655/9128/10946/55696/10929/10569/3183/199746/6635/10084/10713/55119/29896/10450/6430/220988/988/22916/23451/4809/3303/51362/3310/11017/3178/9775/5356/9879/51503/6426/6631/6626/8559/51340/6432/9716/51691/27316/84950/51639/4686/22827/57187/10286/55660/3312/58517/6431/6637/6428/1665/10772/8896/10285/10907/3304/10189/6627/4116/9410/7919/6633/6434 |
| hsa05218 | Melanoma | 63 | 0.3224063 | 1.727485063 | 0.0079338 | 0.0228271 | 0.011648 | 2291 | tags=29%, list=13%, signal=25% | 5159/2260/5155/10000/2246/4616/56034/4286/2255/2252/5293/5156/3082/5154/2247/207/3479/208 |
| hsa05166 | Human T-cell leukemia virus 1 infection | 190 | 0.2771233 | 1.726395191 | 0.0004397 | 0.0020315 | 0.0010366 | 4859 | tags=42%, list=28%, signal=30% | 4215/7043/8829/7094/10000/6688/3554/1959/7040/108/4776/4791/3689/6722/4772/3383/5293/196883/920/115/2113/5971/64784/3718/7042/8379/5602/9020/8517/5566/1387/200186/6777/5970/8498/10488/1030/207/7132/111/3683/6776/208/4792/23373/55697/2033/2002/4790/811/4049/29882/958/8295/113/3560/1388/7046/10524/3601/4801/246184/3716/598/595/896/4055/6908/6929/468/7048/7538/3559/107/109/1031/5604/64764/8503/4088 |
| hsa04622 | RIG-I-like receptor signaling pathway | 53 | 0.3334843 | 1.722556599 | 0.0080311 | 0.0229062 | 0.0116883 | 8653 | tags=77%, list=51%, signal=38% | 5600/5602/55593/8517/5970/7187/7186/9755/4792/79671/3665/4790/23586/3661/1540/8737/79132/4793/64135/5300/6300/8717/9636/9641/340061/64343/3551/7189/7124/57506/1654/6885/8772/5601/1432/26007/4214/5603/7706/10010/29110 |
| hsa04668 | TNF signaling pathway | 106 | 0.2957562 | 1.722065291 | 0.0042057 | 0.0140761 | 0.0071826 | 7140 | tags=59%, list=42%, signal=35% | 4323/7424/1435/10000/5600/4318/7412/6347/9021/3383/5293/7185/5602/9020/8517/5609/6401/5970/10488/207/7132/7187/7186/10454/208/4792/7128/7133/4790/8837/4049/6376/8737/1388/153090/8986/468/6300/8717/5604/64764/8503/5606/3726/3551/3725/1385/1326/23118/7124/6352/182/5290/5594/11035/6885/8772/5295/5601/1051/1386/2277/1432 |
| hsa04915 | Estrogen signaling pathway | 112 | 0.2919939 | 1.716652725 | 0.0029443 | 0.0104971 | 0.0053563 | 7288 | tags=60%, list=43%, signal=35% | 4313/2771/10000/4318/2775/108/6464/399694/5293/196883/115/3708/1509/5914/5241/5566/10488/4846/5330/2885/207/3762/111/208/808/8648/801/2778/113/2099/1388/3305/3326/5580/468/6714/107/109/5894/5604/64764/8503/5443/3306/8202/6667/6654/3725/1385/2289/596/112/5290/1956/5594/2770/5605/7184/3320/5295/3303/1386/6655/3310/2776/3265/10499 |
| hsa04724 | Glutamatergic synapse | 103 | 0.2955727 | 1.71547717 | 0.0039864 | 0.01362 | 0.0069499 | 2185 | tags=26%, list=13%, signal=23% | 2771/1742/85358/2775/108/50944/2791/9454/59345/196883/115/2793/7220/2901/3708/8605/10681/54331/5566/5338/2912/2788/5330/6507/111/2782/57030 |
| hsa04071 | Sphingolipid signaling pathway | 113 | 0.2905663 | 1.709616815 | 0.0044245 | 0.0145125 | 0.0074053 | 7278 | tags=60%, list=43%, signal=35% | 1901/2771/1903/9294/10000/5600/2768/8877/53637/5293/134/1509/5521/5602/2207/5970/5338/2534/4846/5330/207/7132/8560/7186/9846/6609/624/208/91012/5526/4790/29956/5528/8879/5880/79603/5518/5170/4363/5579/10715/387/55844/6300/5894/8717/5604/8503/5879/6093/7124/596/5290/5594/2770/56848/427/8439/5605/2206/5295/5601/10558/5529/2776/1432/3265/5581 |
| hsa05110 | Vibrio cholerae infection | 47 | 0.3361597 | 1.707455578 | 0.0149915 | 0.0369714 | 0.0188654 | 8272 | tags=74%, list=48%, signal=39% | 115/60/5566/537/29927/5336/534/527/2778/535/8992/529/5335/7082/109/51606/375/9114/245972/9601/528/11014/526/10945/51382/10952/10312/23480/9414/9296/9550/71/155066/90423/523 |
| hsa04912 | GnRH signaling pathway | 82 | 0.3038796 | 1.698755884 | 0.0084783 | 0.0237683 | 0.0121282 | 7249 | tags=61%, list=42%, signal=35% | 4313/4323/4215/5600/108/196883/115/3708/8605/5602/5566/5609/5598/5338/5330/2885/111/2002/808/801/2778/113/818/4216/775/5580/2767/5579/468/2185/6714/6300/107/109/5894/5604/5606/1958/815/6654/3725/112/1956/5594/5605/5601/6655/2776/1432/3265 |
| hsa05222 | Small cell lung cancer | 88 | 0.3006866 | 1.698707896 | 0.0054986 | 0.0173416 | 0.0088489 | 2976 | tags=32%, list=17%, signal=26% | 1284/3910/1282/3913/10000/2335/3915/4616/5293/3908/3912/5915/7185/8517/5970/3688/7709/1030/207/7187/7186/208/4792/4790/4149/6257/6256/3914 |
| hsa04370 | VEGF signaling pathway | 57 | 0.3236843 | 1.697117856 | 0.0142157 | 0.0355936 | 0.0181623 | 8318 | tags=72%, list=49%, signal=37% | 10000/5600/3791/8877/5293/9261/8605/4846/207/5336/208/5880/5829/842/5335/5579/6714/6300/5894/5604/8503/5879/3315/5533/4773/5532/5290/5747/5594/56848/5605/5295/9047/1432/3265/5595/7422/7867/5603/5534/572 |
| hsa04925 | Aldosterone synthesis and secretion | 86 | 0.3011712 | 1.694438241 | 0.0056482 | 0.0174774 | 0.0089182 | 7381 | tags=62%, list=43%, signal=35% | 4881/5138/8912/5587/108/482/493/196883/115/3708/57172/8536/5566/25865/10488/5330/221955/3762/185/111/808/3777/801/2778/113/818/477/1388/775/2767/5579/468/107/109/64764/5443/1589/815/84876/1385/1583/112/466/8913/814/3991/23683/1386/2776/3949/57118/5581/491 |
| hsa04210 | Apoptosis | 129 | 0.2812556 | 1.687600589 | 0.0021295 | 0.008122 | 0.0041444 | 7391 | tags=60%, list=43%, signal=35% | 5414/7846/1513/10000/4616/3563/1514/1522/8722/5293/3708/1509/1508/60/4803/7185/5602/9020/8517/5970/1439/6709/4914/207/7132/7186/208/4000/4792/1616/27429/4790/8837/1519/10018/8737/142/153090/842/598/5170/4170/468/823/5894/8717/5604/8503/84823/1647/2081/1075/3551/3725/597/7124/596/10376/824/5290/5594/56616/1676/5605/9451/10038/100506742/8772/5295/5601/84790/835/331/8743/5551/3265/71/9131 |
| hsa05220 | Chronic myeloid leukemia | 76 | 0.3051088 | 1.682042258 | 0.0133336 | 0.0344363 | 0.0175718 | 6212 | tags=55%, list=36%, signal=35% | 7043/10000/7040/4616/6464/399694/5293/25/861/7042/8517/6777/5970/2885/207/9846/6776/1487/208/4792/4790/7046/613/867/1399/598/369/595/1488/7048/1398/5894/5604/8503/4088/1647/6654/1027/3551/5290/1871/5594 |
| hsa04350 | TGF-beta signaling pathway | 88 | 0.296888 | 1.677247936 | 0.0067703 | 0.020006 | 0.0102084 | 6318 | tags=53%, list=37%, signal=34% | 8646/2200/56963/7043/4052/1634/3624/7040/7057/10468/26585/90/7042/353500/2331/1387/4092/392255/1030/57817/2033/57154/8200/654/7046/4086/60436/659/5518/658/7048/4681/387/8454/4088/656/151449/91/6093/6667/9372/1874/3625/7124/79875/5594/83729 |
| hsa04640 | Hematopoietic cell lineage | 75 | 0.303448 | 1.668683234 | 0.011827 | 0.0315386 | 0.0160932 | 1490 | tags=23%, list=9%, signal=21% | 947/3678/1435/3554/1436/2323/3563/3684/929/2209/3690/920/951/3566/3672/945/7066 |
| hsa04727 | GABAergic synapse | 71 | 0.3007184 | 1.645295585 | 0.0131962 | 0.034352 | 0.0175288 | 4746 | tags=42%, list=28%, signal=31% | 2771/2775/108/2791/59345/196883/115/6529/2793/2563/23710/10681/54331/5566/2788/111/2782/2790/113/11337/5334/94235/66008/57468/775/2752/5579/6714/107/109 |
| hsa04917 | Prolactin signaling pathway | 62 | 0.3082321 | 1.642880959 | 0.0147788 | 0.0367231 | 0.0187387 | 7566 | tags=66%, list=44%, signal=37% | 10000/5600/6464/9021/399694/5293/5602/6777/5970/2885/207/6776/208/4790/6774/2099/2592/595/9655/6714/6300/2645/5894/5604/8503/6772/2309/6654/5290/5594/5605/30837/5295/5601/6655/5617/1432/2932/3265/1154/5595 |
| hsa04137 | Mitophagy - animal | 70 | 0.2999556 | 1.640825003 | 0.0177504 | 0.0424972 | 0.021685 | 7249 | tags=63%, list=42%, signal=36% | 22808/338382/4286/7030/8408/23710/10370/5602/5970/10133/79735/79065/55626/65018/7316/7942/7314/9927/8878/11337/7879/6237/8678/598/468/6714/23786/643246/54543/2309/6667/1460/4077/3725/3091/81631/440738/10241/9451/5601/8887/1457/11345/3265 |
| hsa04750 | Inflammatory mediator regulation of TRP channels | 89 | 0.2895101 | 1.638115147 | 0.0137717 | 0.034916 | 0.0178166 | 4851 | tags=42%, list=28%, signal=30% | 51393/5600/3554/59341/108/3356/5293/196883/115/3708/8605/4803/5602/5566/5583/5330/4914/3479/111/5336/624/808/801/2778/3269/113/818/623/3357/5335/5580/5579/6714/6300/107/109/8503 |
| hsa05231 | Choline metabolism in cancer | 94 | 0.2856384 | 1.63140359 | 0.0105897 | 0.0289452 | 0.0147698 | 6959 | tags=56%, list=41%, signal=34% | 5159/5155/10000/56034/23396/7454/8613/8611/5293/9162/8605/5602/5156/5338/5900/5154/2885/207/208/10163/57153/5130/5880/7249/10810/2475/5335/5170/8525/5579/5894/5604/8503/5879/80310/8936/7248/6667/6654/3725/8527/6009/5290/1956/5594/6583/3091/8394/9468/5605/5295/5601/6655 |
| hsa04012 | ErbB signaling pathway | 79 | 0.293139 | 1.628393717 | 0.0131829 | 0.034352 | 0.0175288 | 9514 | tags=78%, list=56%, signal=35% | 10000/6464/399694/5293/25/5602/6777/5609/5063/2885/207/5336/6776/208/2002/8440/27/818/867/1399/2475/5335/369/5579/6714/1398/5894/5604/8503/815/868/6654/1027/3725/5290/5747/1956/5594/10718/4690/5062/5605/10298/5295/5601/6655/2065/2932/3265/5058/5595/572/673/6198/2064/56924/6416/816/5578/2549/6199/25759 |
| hsa04068 | FoxO signaling pathway | 119 | 0.2742969 | 1.627513642 | 0.0099489 | 0.0274222 | 0.0139927 | 7566 | tags=61%, list=44%, signal=34% | 1901/7043/10000/5600/7040/4616/9454/5293/23710/3667/7042/5602/604/1387/1030/2885/207/114907/3479/1032/208/2033/1454/6774/4303/3480/10365/10018/1263/11337/7046/6648/369/595/5170/3643/7048/6300/2308/5894/5604/10769/8503/4088/6446/1647/2309/5571/6654/1027/3551/80854/5290/6789/1956/5594/5565/5934/5605/5295/3575/5601/3586/6655/8743/92579/1432/7874/11345/3265/901/23411/5595 |
| hsa04924 | Renin secretion | 60 | 0.3048095 | 1.61443655 | 0.0175354 | 0.0422913 | 0.02158 | 2907 | tags=32%, list=17%, signal=26% | 4881/2771/2983/2982/5153/5136/134/358/3708/1508/1909/5566/5330/117/185/111/808/801/2778 |
| hsa04530 | Tight junction | 153 | 0.2643946 | 1.610632017 | 0.0056143 | 0.0174774 | 0.0089182 | 4610 | tags=39%, list=27%, signal=28% | 83700/10398/154810/4478/7846/11346/8777/87/58494/11149/7454/4628/3059/4629/4627/84552/60/51421/5872/5521/861/5010/23370/5602/5566/5609/3996/3688/93643/9181/1740/7122/10095/5962/84952/55114/81/10109/140465/4218/7074/8189/4637/123720/8531/10093/2017/595/5518/912/56288/9223/7430/7082/7408/6714/387/55844/10096 |
| hsa03015 | mRNA surveillance pathway | 86 | 0.2850863 | 1.603941671 | 0.0121039 | 0.0320168 | 0.0163372 | 10465 | tags=85%, list=61%, signal=33% | 140886/132430/5521/53918/10482/23293/5976/2521/5526/22985/23381/5528/79869/80335/9939/8189/55308/11269/23283/10921/5518/8761/23435/9887/81608/8106/22794/55844/26019/10978/55339/26528/2733/65109/11052/51585/29894/8731/29101/1478/1477/5411/22916/5529/65110/10898/9775/84305/10914/23049/53981/10250/5516/8732/55012/4686/2107/51692/124540/5499/64895/5527/10284/5523/10767/10189/5515/4116/7919/26986/2935/5500/11051 |
| hsa04936 | Alcoholic liver disease | 117 | 0.2714036 | 1.603472173 | 0.0095096 | 0.0264335 | 0.0134882 | 2717 | tags=29%, list=16%, signal=25% | 126129/83439/10000/5600/718/50507/721/929/728/23765/712/719/714/5602/720/9020/8517/5609/5970/51176/207/7132/7187/713/10454/208/4792/23643/4615/10645/4790/148022/114609/3661 |
| hsa04660 | T cell receptor signaling pathway | 99 | 0.2772902 | 1.60011962 | 0.0158023 | 0.0386802 | 0.0197373 | 8233 | tags=68%, list=48%, signal=35% | 10000/5600/4772/5293/920/4794/5602/9020/8517/5609/5970/2534/5063/5777/2885/207/3937/208/4792/4790/8440/7409/4793/5335/5170/7410/387/6300/5894/5604/8503/5533/1493/4773/868/6654/3551/3725/1326/5788/7124/5532/5290/916/940/5594/4690/6885/5062/5605/10298/5295/5601/3586/6655/1432/2932/3265/5133/919/5058/5595/9402/7535/5603/3702/5534 |
| hsa04910 | Insulin signaling pathway | 124 | 0.2643874 | 1.57717997 | 0.0138387 | 0.034916 | 0.0178166 | 7249 | tags=59%, list=42%, signal=34% | 10000/5507/6464/2889/9021/5837/399694/5293/3101/3667/5602/89801/5566/2885/207/3636/23433/23265/5770/3098/10580/208/3632/2002/57521/808/10211/801/5260/9322/2319/8569/7249/32/867/1399/2475/2997/369/5170/3643/5573/79660/2308/1398/2645/5894/5604/8503/9470/5571/7248/868/6654/3551/2872/6009/5256/5577/5290/5594/2203/5565/3991/5605/5261/5295/5601/6655/92579/5792/2932/3265 |
| hsa04261 | Adrenergic signaling in cardiomyocytes | 134 | 0.2597392 | 1.56657322 | 0.0112954 | 0.0303979 | 0.0155111 | 4842 | tags=40%, list=28%, signal=29% | 7169/2771/6324/10000/5600/108/781/146850/482/493/196883/115/5350/5521/23533/6330/5566/6546/10488/5330/207/70/185/111/1390/7171/208/5526/808/10411/7168/801/2778/55799/5528/784/488/113/818/477/1388/775/5518/6332/782/468/55844/6300/6262/107/109/146/64764/6548 |
| hsa05235 | PD-L1 expression and PD-1 checkpoint pathway in cancer | 87 | 0.2766992 | 1.561261705 | 0.0179812 | 0.0427379 | 0.0218079 | 9509 | tags=77%, list=56%, signal=34% | 4215/10000/5600/4772/5293/920/4794/8517/5970/5777/207/208/4792/4615/4790/353376/148022/114609/55509/6774/7097/4793/3716/2475/5335/3460/6300/5894/5604/8503/5606/6772/10538/5533/4773/1460/3551/3725/7189/5532/5290/916/940/1956/5594/3091/5605/5295/1457/1432/3265/5781/5133/919/5595/7535/5603/5534/27436/6198/5728/4775/1147/1459/3932/5530/6199 |
| hsa04916 | Melanogenesis | 92 | 0.2732503 | 1.555183152 | 0.0164207 | 0.0398962 | 0.0203578 | 4873 | tags=40%, list=29%, signal=29% | 83439/2771/8321/8325/2775/8322/108/7472/4286/7483/196883/115/1857/5566/1387/10488/1856/2535/5330/51176/111/2033/808/801/2778/7484/113/7474/818/7482/5579/107/109/5894/5604/64764/5443 |
| hsa05417 | Lipid and atherosclerosis | 183 | 0.249751 | 1.550379343 | 0.0044245 | 0.0145125 | 0.0074053 | 7249 | tags=56%, list=42%, signal=33% | 10000/5600/4318/5452/7412/6347/929/4772/3383/5293/3708/5602/8517/5609/6401/5970/114548/4846/5330/207/7132/4973/7187/7186/10454/208/4792/23643/4688/9138/4615/6403/653361/3665/4790/353376/148022/114609/808/3661/19/6774/801/6257/6256/7097/3309/7409/1536/958/10333/818/3654/3305/6348/842/3326/6648/948/598/5335/5170/7410/468/6714/387/6300/8503/5879/9641/3306/2081/5606/815/5533/4773/3551/5906/3725/23118/7189/9619/7124/22926/596/6352/5532/5290/5747/5594/6885/7184/9451/3320/5295/5601/3303/8743/3310/3949/1432/2932/3265 |
| hsa04150 | mTOR signaling pathway | 147 | 0.2455979 | 1.489715687 | 0.0135259 | 0.0346602 | 0.017686 | 7249 | tags=56%, list=42%, signal=33% | 8321/8325/10000/8322/7472/6196/7483/5293/8408/3667/1857/79109/1856/2535/10670/2885/207/7132/201163/3479/208/652968/534/57521/6249/3480/7484/7474/7249/7482/2475/529/9706/5170/92335/3643/58528/5579/8131/84335/2887/387/64121/220441/5894/5604/51606/8503/9470/9681/51719/55004/6446/6520/7248/528/6654/3551/526/6396/4041/7124/6009/54541/5290/5594/51382/83667/5605/28956/79726/5295/9296/23175/6655/9675/9663/10325/9550/1975/8324/2932/3265 |
| hsa00010 | Glycolysis / Gluconeogenesis | 51 | -0.204163 | -1.776992401 | 0.0052427 | 0.0168588 | 0.0086025 | 10737 | tags=88%, list=63%, signal=33% | 2203/219/5315/9562/92579/2821/83440/5162/221/5213/5211/5214/223/10327/130589/2023/2027/84532/217/1738/501/55276/128/2597/5230/7167/5106/3099/3939/1737/5160/226/55902/5236/224/387712/230/80201/3945/8789/125/160287/218/5105/5313 |
| hsa01230 | Biosynthesis of amino acids | 60 | -0.185966 | -1.821087641 | 0.0041992 | 0.0140761 | 0.0071826 | 11449 | tags=90%, list=67%, signal=30% | 48/2026/4548/5832/5315/5091/3421/27430/5213/1431/50/5211/5214/6888/113675/22934/2023/3420/2027/4144/3419/2597/5230/7167/5831/5634/5723/6120/6472/587/2806/7086/226/3417/2805/435/84706/3418/387712/65263/440/875/384/6470/162417/230/95/445/102724560/29968/383/26227/4143/5313 |
| hsa04146 | Peroxisome | 74 | -0.161895 | -1.857630559 | 0.0028325 | 0.010323 | 0.0052675 | 9249 | tags=77%, list=54%, signal=36% | 10005/1891/6647/5052/8443/1384/2182/10901/26061/2181/2180/8504/84769/4598/10478/5189/283927/5194/225/3155/6342/8540/54677/847/11264/3417/390916/10455/5264/51703/23600/5191/5190/26063/55825/3418/84188/3295/1962/8800/373156/92960/51/5828/25824/5825/5827/11001/55711/83594/51268/8528/8309/570/189/255027/23305 |
| hsa04742 | Taste transduction | 48 | -0.220458 | -1.926742176 | 0.0044986 | 0.0146092 | 0.0074547 | 2844 | tags=52%, list=17%, signal=44% | 5030/6328/83756/5567/954/10021/259295/6337/50840/5028/54429/22953/50832/255022/5726/80835/2911/2914/40/3352/6340/29850/5332/342372/6338 |
| hsa00100 | Steroid biosynthesis | 20 | -0.340732 | -1.94277948 | 0.0058528 | 0.0177752 | 0.0090701 | 7096 | tags=80%, list=42%, signal=47% | 4047/1594/1717/3930/7108/1718/6309/10682/51478/6713/120227/2222/6307/1591/8435/1056 |
| hsa05204 | Chemical carcinogenesis - DNA adducts | 32 | -0.258685 | -1.981629454 | 0.0064802 | 0.0193228 | 0.0098598 | 3711 | tags=59%, list=22%, signal=47% | 373156/2947/2953/2950/6818/1646/1551/2948/1558/6817/4258/5743/1548/1576/1577/7365/54578/2938/2944 |
| hsa05033 | Nicotine addiction | 26 | -0.300861 | -2.042604692 | 0.0084323 | 0.0237683 | 0.0121282 | 7686 | tags=85%, list=45%, signal=47% | 8973/2570/2890/55879/116443/2903/2905/1141/2566/2893/2906/2562/2564/2891/1139/116444/774/2904/2902/2569/2561/2568 |
| hsa00620 | Pyruvate metabolism | 37 | -0.307835 | -2.374248634 | 0.0003998 | 0.0019005 | 0.0009698 | 10629 | tags=95%, list=62%, signal=36% | 3029/219/5315/5091/5162/223/10327/4190/31/84532/217/4191/1738/501/98/128/5106/3939/1737/5160/55902/2271/38/224/39/97/2739/4199/3945/125/10873/4200/160287/5105/5313 |
