# Supplementary material for: The Potential Dual Role of H2.0-like Homeobox in the Tumorgenesis and Development of Colorectal Cancer and Its Prognostic Value
Source: Can J Gastroenterol Hepatol. 2023 Sep 9;2023:5521544. doi: 10.1155/2023/5521544 (PMC10505080; doi:10.1155/2023/5521544)
Supplement: Supplementary Materials — Table S1: The result of FIMO prediction. Table S2: Detailed results of significantly enriched pathways in HLX high expression group. Table S3: The relative proportions of 22 immune infiltrating cells of CRC samples in TCGA cohort. [file 5521544.f1.zip › Table S3.docx]

**Table S3** The relative proportions of 22 immune infiltrating cells of CRC samples in TCGA cohort

|  | B.cells.naive | B.cells.memory | Plasma.cells | T.cells.CD8 | T.cells.CD4.naive | T.cells.CD4.memory.resting | T.cells.CD4.memory.activated | T.cells.follicular.helper | T.cells.regulatory..Tregs. | T.cells.gamma.delta | NK.cells.resting | NK.cells.activated | Monocytes | Macrophages.M0 | Macrophages.M1 | Macrophages.M2 | Dendritic.cells.resting | Dendritic.cells.activated | Mast.cells.resting | Mast.cells.activated | Eosinophils | Neutrophils |
| --- | --- | --- | --- | --- | --- | --- | --- | --- | --- | --- | --- | --- | --- | --- | --- | --- | --- | --- | --- | --- | --- | --- |
| TCGA-AG-A025-01A | 0.0333047 | 0.0220514 | 0.0117086 | 0.1466686 | 0 | 0.0767079 | 0.0057381 | 0.090225 | 0.0501855 | 0 | 0.1294836 | 0 | 0.0853926 | 0.1268875 | 0.011484 | 0.1090795 | 0.003042 | 0 | 0 | 0.0859998 | 0.0120411 | 0 |
| TCGA-EI-6884-01A | 0.0274195 | 0 | 0.1100926 | 0.0864324 | 0 | 0.2071474 | 0 | 0.0547242 | 0.0021985 | 0 | 0.003945 | 0.0173588 | 0 | 0.0959457 | 0.0711323 | 0.2342166 | 0.0186205 | 0 | 0.0594836 | 0 | 0 | 0.0112828 |
| TCGA-AG-3582-01A | 0.024138 | 0 | 0.073095 | 0.0438732 | 0 | 0.1426115 | 0.0770627 | 0.0385425 | 0.0188182 | 0 | 0 | 0.0114776 | 0.0050069 | 0.0834159 | 0.0761447 | 0.2713108 | 0.0018304 | 0 | 0 | 0.1326727 | 0 | 0 |
| TCGA-DC-6154-01A | 0.0140138 | 0.0146281 | 0.0420518 | 0.0679736 | 0 | 0.1282263 | 0.0355063 | 0.0245912 | 0.0136773 | 0 | 0.0622116 | 0.0223381 | 0.0529192 | 0.1857185 | 0.0114612 | 0.3119331 | 0 | 0 | 0 | 0.0127497 | 0 | 0 |
| TCGA-AG-3611-01A | 0.0049697 | 0 | 0.0967778 | 0.1204993 | 0 | 0.1337087 | 0 | 0.0703366 | 0.0470154 | 0 | 0.0217261 | 0 | 0.0190436 | 0.0168356 | 0.0249419 | 0.2806492 | 0.0648381 | 0 | 0 | 0.098658 | 0 | 0 |
| TCGA-AG-3609-01A | 0.0059605 | 0.0503507 | 0.0014878 | 0.158513 | 0 | 0 | 0.0378862 | 0.0328707 | 0.0674002 | 0 | 0.0274387 | 0 | 0.0198182 | 0.1619034 | 0.0153188 | 0.2414159 | 0.0094372 | 0 | 0 | 0.1701987 | 0 | 0 |
| TCGA-AG-A01L-01A | 0.0532238 | 0 | 0.0524437 | 0.1434194 | 0 | 0.1901279 | 0 | 0.0542396 | 0.0749858 | 0 | 0 | 0.0894529 | 0.0084684 | 0.0845991 | 0.0249677 | 0.1444415 | 0 | 0.0057219 | 0.0739084 | 0 | 0 | 0 |
| TCGA-AG-3742-01A | 0 | 0 | 0.0319575 | 0.0322224 | 0 | 0.1474266 | 0 | 0.0692106 | 0.005031 | 0 | 0.1378006 | 0 | 0 | 0.1723349 | 0.06333 | 0.2772998 | 0 | 0.0154806 | 0.0237447 | 0.0032612 | 0 | 0.0208999 |
| TCGA-AG-A01W-01A | 0.1318923 | 0 | 0.0421236 | 0.1384158 | 0 | 0.0362929 | 0 | 0.1703706 | 0.05879 | 0 | 0 | 0.0460861 | 0 | 0.0698316 | 0.1132801 | 0.1533575 | 0.0366679 | 0 | 0.0028916 | 0 | 0 | 0 |
| TCGA-AG-A02N-01A | 0.004748 | 0.0021867 | 0.0841751 | 0.1363568 | 0 | 0.1922735 | 0.0113886 | 0.1488184 | 0.0038412 | 0.0271116 | 0 | 0.0301138 | 0.0002752 | 0.0097369 | 0.0798455 | 0.1855772 | 0.0090532 | 0 | 0 | 0.0744984 | 0 | 0 |
| TCGA-AH-6643-01A | 0.016982 | 0 | 0 | 0.13099 | 0 | 0 | 0 | 0.0820309 | 0.0398449 | 0 | 0.0453071 | 0.0011164 | 0.0481381 | 0.3066413 | 0 | 0.0443341 | 0 | 0 | 0 | 0.1865569 | 0 | 0.0980584 |
| TCGA-AH-6549-01A | 0.0339678 | 0 | 0.0443849 | 0.0160122 | 0 | 0.3052193 | 0.0687481 | 0.0154289 | 0.0064494 | 0 | 0.0592714 | 0 | 0.0136904 | 0.0695632 | 0.0926269 | 0.2728583 | 0 | 0 | 0 | 0 | 0 | 0.0017792 |
| TCGA-AH-6903-01A | 0.0129839 | 0.0369399 | 0.0519541 | 0.0855359 | 0 | 0.3546553 | 0.0811971 | 0 | 0.0155448 | 0 | 0.1218903 | 0 | 0.0486695 | 0.0226658 | 0 | 0.0349446 | 0.0019155 | 0.0269732 | 0 | 0.0796384 | 0.0244917 | 0 |
| TCGA-AG-3727-01A | 0.1665251 | 0 | 0.0834973 | 0.0670696 | 0 | 0.2131347 | 0 | 0.020458 | 0.0675341 | 0 | 0.0675496 | 0 | 0.0260405 | 0.0682152 | 0.0096334 | 0.1507031 | 0 | 0 | 0 | 0.0468354 | 0 | 0.0128041 |
| TCGA-F5-6863-01A | 0.0205775 | 0.0557098 | 0 | 0.1050808 | 0 | 0.1019254 | 0 | 0.0518715 | 0.0125522 | 0 | 0 | 0.0407629 | 0.022397 | 0.2777769 | 0.0226461 | 0.2544542 | 0 | 0.0147538 | 0.0194918 | 0 | 0 | 0 |
| TCGA-AG-3887-01A | 0.0289603 | 0 | 0.0169549 | 0.2757351 | 0 | 0.111648 | 0.0293883 | 0.0896341 | 0.0613014 | 0 | 0.062264 | 0 | 0.0403753 | 0.0114847 | 0.0234243 | 0.0893685 | 0.0194231 | 0 | 0 | 0.140038 | 0 | 0 |
| TCGA-AG-A01J-01A | 0.0188926 | 0 | 0.1122242 | 0.2014901 | 0 | 0.0693312 | 0 | 0.1447261 | 0.0837219 | 0 | 0 | 0.0922394 | 0.0171125 | 0.1298177 | 0.0257096 | 0.0977128 | 0 | 0 | 0.0007879 | 0.006234 | 0 | 0 |
| TCGA-AF-5654-01A | 0.0056512 | 0 | 0.0585935 | 0.0648172 | 0 | 0.325604 | 0.0593699 | 0.0685049 | 0.0201867 | 0 | 0.0545085 | 0 | 0.0197154 | 0.0324073 | 0.0408602 | 0.1045313 | 0 | 0.0332023 | 0 | 0.1120477 | 0 | 0 |
| TCGA-DY-A1DE-01A | 0.0604882 | 0 | 0.0063409 | 0.0800627 | 0 | 0.0489536 | 0 | 0.0831753 | 0.0389992 | 0 | 0 | 0.0376995 | 0 | 0.3301593 | 0.0631849 | 0.2437384 | 0 | 0 | 0.007198 | 0 | 0 | 0 |
| TCGA-AF-3913-01A | 0.0284587 | 0 | 0.0228758 | 0.0083431 | 0 | 0.1921656 | 0.068329 | 0.030623 | 0.0041197 | 0 | 0.0549618 | 0 | 0.0348838 | 0.0901896 | 0.0196375 | 0.2995993 | 0 | 0.0072644 | 0 | 0.1013425 | 0.0040634 | 0.0331427 |
| TCGA-AG-A014-01A | 0 | 0.0815209 | 0.0231258 | 0.0236653 | 0 | 0.0734102 | 0 | 0.0833939 | 0.043632 | 0 | 0 | 0.009077 | 0.0014602 | 0.1854084 | 0.0600728 | 0.2420007 | 0 | 0 | 0 | 0.1454781 | 0 | 0.0277548 |
| TCGA-AG-3592-01A | 0.0256207 | 0 | 0.0379764 | 0.0656399 | 0 | 0.242689 | 0.050142 | 0.0384221 | 0.0220883 | 0 | 0.0327858 | 0 | 0 | 0.0899027 | 0.0724664 | 0.2464427 | 0 | 0.0032413 | 0 | 0.068106 | 0 | 0.0044767 |
| TCGA-AG-4015-01A | 0.0560264 | 0 | 0.0615266 | 0.122582 | 0 | 0.0702058 | 0 | 0.0871019 | 0.0566048 | 0 | 0 | 0.027983 | 0.004049 | 0.049183 | 0.0763753 | 0.3158332 | 0.0099356 | 0 | 0 | 0.0625932 | 0 | 0 |
| TCGA-G5-6572-01A | 0.0091133 | 0.0398916 | 0 | 0.0161928 | 0 | 0.1578235 | 0 | 0 | 0.0087136 | 0 | 0.0126027 | 0 | 0 | 0.4110873 | 0.0260902 | 0.2546747 | 0 | 0 | 0.0638104 | 0 | 0 | 0 |
| TCGA-AG-A011-01A | 0.0311643 | 0.0159891 | 0.145831 | 0.1334376 | 0.125191 | 0 | 0 | 0.0270531 | 0.0999108 | 0 | 0.037727 | 0 | 0.0314224 | 0.1043342 | 0 | 0.1944102 | 0.0185614 | 0 | 0 | 0.0349676 | 0 | 0 |
| TCGA-F5-6464-01A | 0.0280565 | 0 | 0 | 0.1298265 | 0 | 0.0963726 | 0 | 0.0421911 | 0.0580056 | 0 | 0 | 0.047612 | 0 | 0.1124406 | 0.0737061 | 0.3302534 | 0.022006 | 0 | 0.0508711 | 0 | 0.00591 | 0.0027486 |
| TCGA-AG-4008-01A | 0.0133119 | 0.0224407 | 0.018478 | 0.0889987 | 0 | 0.0233484 | 0.051454 | 0 | 0.0650198 | 0 | 0.0727608 | 0 | 0.0648547 | 0.1102818 | 0.0046854 | 0.2980423 | 0.0039195 | 0.0072008 | 0 | 0.1332884 | 0 | 0.0219149 |
| TCGA-AG-3731-01A | 0.0604274 | 0 | 0.0094491 | 0.0871259 | 0 | 0.1457466 | 0 | 0.0923085 | 0.0093237 | 0 | 0 | 0.0513319 | 0.0052695 | 0.0751673 | 0.1413877 | 0.2654846 | 0 | 0 | 0.0307312 | 0 | 0.0005556 | 0.0256911 |
| TCGA-AH-6544-01A | 0.2183457 | 0.1513772 | 0.0291326 | 0.0009301 | 0 | 0.0952446 | 0 | 0.1944461 | 0 | 0 | 0 | 0.050483 | 0 | 0.0999788 | 0 | 0.0925544 | 0 | 0.0675075 | 0 | 0 | 0 | 0 |
| TCGA-AG-A020-01A | 0.0193728 | 0.0157612 | 0.0575145 | 0.1812452 | 0 | 0.1050919 | 0.0270314 | 0.1042569 | 0.0254231 | 0 | 0.0813507 | 0 | 0.0270042 | 0.0572565 | 0.0268621 | 0.1435913 | 0 | 0.0080555 | 0 | 0.1131725 | 0.0070101 | 0 |
| TCGA-AG-A026-01A | 0.0237719 | 0 | 0.0979304 | 0.0965694 | 0 | 0.0784695 | 0 | 0.2953336 | 0 | 0 | 0 | 0.0206146 | 0.0158759 | 0.1256521 | 0.0281338 | 0.1705906 | 0 | 0 | 0 | 0.0463295 | 0.0007286 | 0 |
| TCGA-CL-5918-01A | 0.0274443 | 0.0052573 | 0 | 0.0673005 | 0 | 0.3096919 | 0.0211742 | 0.0214127 | 0.0134319 | 0 | 0.0225709 | 0.1861252 | 0.0220803 | 0.1386039 | 0.0335907 | 0.0158552 | 0.0090775 | 0.0001622 | 0.0933924 | 0 | 0 | 0.012829 |
| TCGA-EI-6882-01A | 0.0298638 | 0 | 0.0364225 | 0.0497446 | 0 | 0.3308374 | 0.0210077 | 0.0098066 | 0 | 0 | 0.0517018 | 0.0060827 | 0.0186928 | 0.0920076 | 0.0681046 | 0.1986984 | 0 | 0 | 0 | 0.0809995 | 0 | 0.0060297 |
| TCGA-AG-3883-01A | 0.0368467 | 0 | 0.0269205 | 0.0537148 | 0 | 0.0880619 | 0 | 0.0941346 | 0.078635 | 0 | 0.014731 | 0 | 0.0122877 | 0.1804795 | 0.0184628 | 0.225202 | 0 | 0 | 0 | 0.1456419 | 0.01359 | 0.0112915 |
| TCGA-EI-7004-01A | 0.0276652 | 0.0178979 | 0 | 0.0530394 | 0 | 0.204793 | 0 | 0 | 0.0069291 | 0 | 0.045113 | 0 | 0.0269124 | 0.1515523 | 0.0191195 | 0.3378635 | 0 | 0 | 0 | 0.0895163 | 0 | 0.0195983 |
| TCGA-AF-6136-01A | 0.1357858 | 0 | 0.0663639 | 0.0736863 | 0 | 0.2473832 | 0 | 0.0809795 | 0.007621 | 0 | 0 | 0.0258873 | 0.0307283 | 0.0430354 | 0.0281324 | 0.1267325 | 0.0551626 | 0 | 0.0785018 | 0 | 0 | 0 |
| TCGA-DC-4745-01A | 0.0873682 | 0 | 0.0406438 | 0.0397499 | 0 | 0.3305578 | 0.0058348 | 0.0253565 | 0.0155037 | 0 | 0 | 0.0010324 | 0 | 0.0973997 | 0.0310546 | 0.2536087 | 0.0220349 | 0.0094535 | 0 | 0.018439 | 0.0128445 | 0.009118 |
| TCGA-EI-6883-01A | 0.1911547 | 0 | 0.0752023 | 0.0807015 | 0 | 0.0735159 | 0.0025229 | 0.0533971 | 0.0162826 | 0 | 0 | 0.0553675 | 0 | 0.2757543 | 0.0412747 | 0.1133455 | 0 | 0 | 0.0214808 | 0 | 0 | 0 |
| TCGA-EI-6917-01A | 0.0286 | 0 | 0.0208795 | 0.0682949 | 0 | 0.0426656 | 0.0159869 | 0.0521056 | 0.0345363 | 0 | 0.0657238 | 0.0223872 | 0.0393348 | 0.1514896 | 0.1164273 | 0.3085974 | 0.0267667 | 0 | 0.0062045 | 0 | 0 | 0 |
| TCGA-F5-6864-01A | 0.0514788 | 0 | 0.0334147 | 0.1230593 | 0 | 0.0835279 | 0 | 0.1105305 | 0.0134129 | 0 | 0 | 0.0588083 | 0.0208938 | 0.0703756 | 0.0850363 | 0.2681811 | 0.0421031 | 0 | 0.0391777 | 0 | 0 | 0 |
| TCGA-EI-6508-01A | 0.001441 | 0 | 0.1017433 | 0.0598052 | 0 | 0.2763443 | 0 | 0.049129 | 0.0265474 | 0 | 0 | 0.0513821 | 0.0191389 | 0.0482577 | 0.060823 | 0.2777357 | 0.0042954 | 0 | 0.0233572 | 0 | 0 | 0 |
| TCGA-F5-6465-01A | 0.0469385 | 0 | 0.0076233 | 0.1122803 | 0 | 0.2146636 | 0.0258712 | 0.0553932 | 0.0370097 | 0 | 0.0454026 | 0 | 0.0565472 | 0.1012247 | 0.0687365 | 0.1171638 | 0.0299108 | 0 | 0 | 0.0812346 | 0 | 0 |
| TCGA-AF-6672-01A | 0.0968289 | 0.0684016 | 0.0234843 | 0.1335982 | 0 | 0.1540839 | 0 | 0.1282757 | 0.039602 | 0 | 0.0138653 | 0 | 0.0240184 | 0 | 0.021776 | 0.1622378 | 0.0284123 | 0.0053222 | 0 | 0.1000933 | 0 | 0 |
| TCGA-AG-3885-01A | 0.1139363 | 0 | 0.0258845 | 0.2038391 | 0 | 0 | 0 | 0.0210876 | 0.1870153 | 0 | 0.0662548 | 0 | 0.0301389 | 0.0906353 | 0.028505 | 0.0421732 | 0.0250748 | 0 | 0 | 0.1551929 | 0.0102623 | 0 |
| TCGA-AG-A00H-01A | 0.0392026 | 0 | 0.0136614 | 0.0878871 | 0 | 0.1299896 | 0 | 0 | 0.0856307 | 0 | 0 | 0.0137857 | 0 | 0.2397024 | 0.0155027 | 0.2890037 | 0 | 0 | 0 | 0.0856339 | 0 | 0 |
| TCGA-DC-5337-01A | 0.0116033 | 0 | 0.0252765 | 0.0349444 | 0 | 0.3461246 | 0.0794244 | 0.0968868 | 0.0046294 | 0 | 0.0360896 | 0 | 0.0113849 | 0.0042531 | 0.0747178 | 0.1386095 | 0.0199983 | 0.0083194 | 0 | 0.1077379 | 0 | 0 |
| TCGA-AG-3893-01A | 0.0499002 | 0 | 0.0200209 | 0.1024393 | 0 | 0.064287 | 0 | 0.0455756 | 0.051574 | 0 | 0.0175724 | 0 | 0 | 0.1001634 | 0.0991976 | 0.4049637 | 0.0041261 | 0 | 0.0050648 | 0.025167 | 7.45E-05 | 0.0098733 |
| TCGA-AG-A02G-01A | 0.0544265 | 0 | 0.0538518 | 0.1660422 | 0 | 0.1445829 | 0 | 0.1336209 | 0.0642765 | 0 | 0 | 0.0775798 | 0.0132996 | 0 | 0.0401896 | 0.2038874 | 0 | 0 | 0 | 0.0482429 | 0 | 0 |
| TCGA-AG-3901-01A | 0.0340729 | 0 | 0.0067483 | 0.0443344 | 0 | 0.1221103 | 0.0096998 | 0.0288328 | 0.0833059 | 0 | 0.0324636 | 0 | 0 | 0.2884296 | 0.0442753 | 0.1900368 | 0.0092567 | 0 | 0 | 0.0762292 | 0 | 0.0302044 |
| TCGA-AG-3726-01A | 0.0836929 | 0 | 0.026755 | 0.0855637 | 0 | 0.1742794 | 0.0123834 | 0.1463262 | 0.0020521 | 0 | 0.1121997 | 0 | 0.0101481 | 0.0829533 | 0.0058811 | 0.1296934 | 0 | 0.0292369 | 0 | 0.0858853 | 0 | 0.0129496 |
| TCGA-F5-6810-01A | 0.1073937 | 0 | 0.0422065 | 0.0906613 | 0.0052443 | 0.2491807 | 0 | 0 | 0.0031623 | 0 | 0.0193407 | 0.0208596 | 0.048669 | 0.0515482 | 0.1378475 | 0.195466 | 0.0133191 | 0 | 0 | 0.0151011 | 0 | 0 |
| TCGA-DC-6160-01A | 0.0023893 | 0.0024249 | 0.1043377 | 0.059822 | 0 | 0.2303932 | 0.029475 | 0.1306596 | 0.0126086 | 0 | 0.0343604 | 0 | 0.0039108 | 0.1230503 | 0.043975 | 0.1446015 | 0.0292086 | 0.0067091 | 0 | 0.042074 | 0 | 0 |
| TCGA-AG-3581-01A | 0.0196042 | 0 | 0.0544397 | 0.1143403 | 0 | 0.0999594 | 0 | 0.0717599 | 0.0678522 | 0 | 0 | 0.0480915 | 0.0125098 | 0.0980658 | 0.032913 | 0.3006132 | 0 | 0.007735 | 0.0436102 | 0.0098488 | 0 | 0.0186567 |
| TCGA-F5-6812-01A | 0.0924892 | 0.0088259 | 0 | 0.066403 | 0 | 0.1143085 | 0 | 0.1295637 | 0.039315 | 0 | 0 | 0.0439568 | 0 | 0.11787 | 0.1761549 | 0.1942665 | 0 | 0 | 0.0168463 | 0 | 0 | 0 |
| TCGA-AG-4001-01A | 0.036732 | 0 | 0.0129226 | 0.0617538 | 0 | 0.0959659 | 0 | 0.0696259 | 0.0384234 | 0 | 0 | 0.0429366 | 0.0086086 | 0.1680398 | 0.0710939 | 0.38636 | 0.0024558 | 0 | 0 | 0.0025987 | 0 | 0.0024831 |
| TCGA-AF-2687-01A | 0.0121375 | 0.0157599 | 0.0314212 | 0.1455815 | 0 | 0.0264876 | 0.0415575 | 0.0305265 | 0 | 0 | 0.0394503 | 0 | 0.0460019 | 0.1329313 | 0.1554442 | 0.2149877 | 0.0328189 | 0 | 0 | 0.0578398 | 0 | 0.0170543 |
| TCGA-AH-6644-01A | 0.0584882 | 0 | 0 | 0.1265433 | 0 | 0.0508091 | 0.0121799 | 0.0837848 | 0.0154064 | 0 | 0.0472271 | 0 | 0.0210312 | 0.1084616 | 0.0616452 | 0.2908123 | 0 | 0 | 0.0431754 | 0.0804356 | 0 | 0 |
| TCGA-AG-3586-01A | 0.034779 | 0 | 0.0590131 | 0.078804 | 0 | 0.1457482 | 0 | 0.0748772 | 0.0462603 | 0 | 0.0301659 | 0.0197166 | 0 | 0.0946472 | 0.0594603 | 0.2728134 | 0 | 0 | 0 | 0.079758 | 0 | 0.0039568 |
| TCGA-AG-3909-01A | 0.0689181 | 0 | 0.0386175 | 0.0942531 | 0 | 0.1285676 | 0 | 0.0867437 | 0.0445679 | 0 | 0 | 0.0276817 | 0 | 0.051522 | 0.0287142 | 0.3298593 | 0.0120496 | 0 | 0.0885054 | 0 | 0 | 0 |
| TCGA-AG-3591-01A | 0.0866369 | 0 | 0.0279078 | 0.0373184 | 0 | 0.3286288 | 0 | 0.079899 | 0 | 0 | 0 | 0.0314099 | 0.0102836 | 0.0417249 | 0.0073981 | 0.1992525 | 0.0433645 | 0.018892 | 0 | 0.0428765 | 0.044407 | 0 |
| TCGA-AG-3612-01A | 0.033757 | 0 | 0.026487 | 0.1000862 | 0 | 0.0734695 | 0 | 0.0807872 | 0.0813232 | 0 | 0 | 0.0330261 | 0.0329767 | 0.117253 | 0.0843467 | 0.2398172 | 0.0307526 | 0 | 0.0465188 | 0 | 0.0193988 | 0 |
| TCGA-F5-6814-01A | 0.0700618 | 0 | 0.0339808 | 0.1846406 | 0 | 0.0700086 | 0.0277032 | 0.0959516 | 0.0483645 | 0 | 0.0363048 | 0.0088359 | 0 | 0.0992341 | 0.1369155 | 0.1234907 | 0.020818 | 0 | 0 | 0.0436898 | 0 | 0 |
| TCGA-AG-3605-01A | 0.033949 | 0 | 0.103179 | 0.1545866 | 0 | 0.1054557 | 0.0079328 | 0.0667729 | 0.0875173 | 0 | 0 | 0.0081051 | 0.0201564 | 0 | 0.0034922 | 0.263048 | 0 | 0.0123076 | 0 | 0.0993996 | 0 | 0.0340978 |
| TCGA-EI-6513-01A | 0.0417323 | 0 | 0.0573412 | 0.1036058 | 0 | 0.1317767 | 0 | 0.1262382 | 0 | 0 | 0 | 0.0509952 | 0 | 0.1409149 | 0.0750283 | 0.2534243 | 0 | 0 | 0.0123714 | 0 | 0 | 0.0065718 |
| TCGA-DC-6158-01A | 0.0585249 | 0 | 0.007174 | 0.0900577 | 0 | 0.1305056 | 0.1037205 | 0.0478455 | 0.019158 | 0 | 0.0448465 | 0 | 0.0207161 | 0.1110021 | 0.0653285 | 0.2234563 | 0.0018113 | 0 | 0 | 0.0758531 | 0 | 0 |
| TCGA-EI-6512-01A | 0.0131607 | 0 | 0.0332562 | 0.0461668 | 0 | 0.1956472 | 0 | 0.0622291 | 0.0408249 | 0 | 0.0073085 | 0.0643772 | 0.0116254 | 0.0788495 | 0.090011 | 0.278313 | 0 | 0.0096106 | 0.0382696 | 0.0025373 | 0 | 0.027813 |
| TCGA-AG-3601-01A | 0.0498993 | 0 | 0.0727313 | 0.1180111 | 0 | 0 | 0.0093304 | 0.0471671 | 0.0339631 | 0 | 0 | 0 | 0 | 0.0257181 | 0.0579598 | 0.4490521 | 0.022782 | 0 | 0 | 0.0979085 | 0 | 0.0154772 |
| TCGA-CI-6622-01A | 0.0509117 | 0 | 0.0926326 | 0.0476103 | 0 | 0.1425585 | 0.0897581 | 0 | 0 | 0 | 0.0194477 | 0 | 0.0366996 | 0.1052166 | 0 | 0.3294639 | 0 | 0.0058733 | 0 | 0.0798277 | 0 | 0 |
| TCGA-BM-6198-01A | 0.0995003 | 0 | 0.0801834 | 0.0863284 | 0 | 0.1394289 | 0.0646516 | 0.0654485 | 0 | 0 | 0.053345 | 0 | 0 | 0.0998426 | 0.0471938 | 0.1511034 | 0 | 0 | 0 | 0.1102458 | 0 | 0.0027282 |
| TCGA-AG-3881-01A | 0.0695976 | 0 | 0.0094746 | 0.084323 | 0 | 0.0918808 | 0 | 0.0696247 | 0.0301407 | 0 | 0.0399857 | 0 | 0.055183 | 0.1957316 | 0.0432237 | 0.1738785 | 0.0154694 | 0 | 0 | 0.0576793 | 0.0512301 | 0.0125774 |
| TCGA-DY-A1DC-01A | 0.0196692 | 0.0515052 | 0 | 0.1437759 | 0 | 0.1527301 | 0.0273598 | 0.0409416 | 0.0822458 | 0 | 0.0259616 | 0.0132694 | 0.0519358 | 0.0744318 | 0.0657515 | 0.1480948 | 0.0004831 | 0 | 0 | 0.0924973 | 0.0093472 | 0 |
| TCGA-DY-A1DD-01A | 0 | 0.1064197 | 0.0592897 | 0.0559535 | 0.1764502 | 0 | 0.0057005 | 0.0538261 | 0 | 0.007006 | 0.026613 | 0.0728011 | 0 | 0.3147831 | 0 | 0.0414087 | 0.0207138 | 0 | 0 | 0.0590346 | 0 | 0 |
| TCGA-AG-3600-01A | 0.0205906 | 0 | 0.0327254 | 0.121229 | 0 | 0.1321996 | 0 | 0.0588718 | 0.0719247 | 0 | 0.0091797 | 0.0073252 | 0.0023485 | 0.0578536 | 0.0273541 | 0.3254361 | 0.012289 | 0 | 0 | 0.1068692 | 0 | 0.0138034 |
| TCGA-AG-3599-01A | 0.0223667 | 0 | 0.0512843 | 0.2058802 | 0 | 0.0064017 | 0.021354 | 0.0602204 | 0.1295321 | 0 | 0.0172642 | 0.0364357 | 0.0275952 | 0.0155854 | 0.0605795 | 0.235431 | 0.0063709 | 0 | 0 | 0.1036988 | 0 | 0 |
| TCGA-AG-3584-01A | 0.0130741 | 0.0445121 | 0.0324998 | 0.1196023 | 0 | 0.0026055 | 0 | 0.0355381 | 0.1589451 | 0 | 0.0279072 | 0.0011909 | 0 | 0.2079361 | 0.0242916 | 0.1992984 | 0.0125538 | 0 | 0 | 0.120045 | 0 | 0 |
| TCGA-DC-6156-01A | 0.0275216 | 0 | 0.0065289 | 0.2423485 | 0 | 0.0121417 | 0 | 0.03926 | 0.0438976 | 0 | 0.020398 | 0 | 0.0066024 | 0.1225154 | 0.0532777 | 0.3914927 | 0.0009573 | 0 | 0 | 0.0174073 | 0 | 0.0156511 |
| TCGA-DT-5265-01A | 0.0044117 | 0.035366 | 0 | 0.043758 | 0.0056012 | 0 | 0 | 0.0310247 | 0.0401335 | 0 | 0.0158636 | 0.0214788 | 0.0055297 | 0.2751474 | 0 | 0.4193931 | 0.0265623 | 0 | 0.0679948 | 0 | 0 | 0.0077353 |
| TCGA-AG-3902-01A | 0.0073414 | 0.0062456 | 0.0212591 | 0.2271817 | 0 | 0 | 0.009825 | 0.1312617 | 0.0229223 | 0 | 0.0192845 | 0.0191078 | 0.0365297 | 0 | 0.0647974 | 0.3411979 | 0.0218958 | 0.0133946 | 0 | 0.0268004 | 0.030955 | 0 |
| TCGA-AG-A036-01A | 0.0668401 | 0 | 0.0427422 | 0.1261052 | 0 | 0.0057961 | 0.0498051 | 0.1113469 | 0.041034 | 0 | 0.0627563 | 0 | 0 | 0.2509685 | 0.0687668 | 0.1051958 | 0 | 0 | 0 | 0.0686431 | 0 | 0 |
| TCGA-AG-3593-01A | 0.0525169 | 0 | 0.0777765 | 0.0774938 | 0 | 0.1363587 | 0.0326576 | 0.0551328 | 0.0626806 | 0 | 0.0253954 | 0.0025644 | 0.0243168 | 0.0206288 | 0.0735124 | 0.2666845 | 0.0263999 | 0 | 0 | 0.0658808 | 0 | 0 |
| TCGA-AG-A01Y-01A | 0.0357707 | 0.0294571 | 0.0363924 | 0.1510272 | 0 | 0.0249337 | 0.0055261 | 0.0925939 | 0.0510557 | 0 | 0 | 0.0287604 | 0 | 0.2493751 | 0.0559276 | 0.1438262 | 0.0087284 | 0 | 0 | 0.0866253 | 0 | 0 |
| TCGA-AG-3574-01A | 0.0201765 | 0 | 0.076468 | 0.1455907 | 0 | 0.0505786 | 0 | 0.116031 | 0.0291581 | 0 | 0.0454104 | 0 | 0.0242205 | 0.0480605 | 0.039116 | 0.339331 | 0.0027346 | 0 | 0 | 0.0585022 | 0 | 0.004622 |
| TCGA-F5-6571-01A | 0.035327 | 0 | 0.0133852 | 0.0729009 | 0 | 0.1925418 | 0 | 0.0489985 | 0.0463073 | 0 | 0.0109327 | 0 | 0.0135689 | 0.0898334 | 0.0572498 | 0.2699121 | 0.084438 | 0.0093735 | 0.0552308 | 0 | 0 | 0 |
| TCGA-DC-6683-01A | 0.0992028 | 0 | 0.001394 | 0.0124609 | 0 | 0.158505 | 0 | 0.0743843 | 0.0356099 | 0 | 0.0032969 | 0.0008218 | 0 | 0.268069 | 0.0634764 | 0.2656492 | 0 | 0 | 0.0171298 | 0 | 0 | 0 |
| TCGA-AF-6655-01A | 0.0282002 | 0 | 0.0664561 | 0.0061687 | 0 | 0.1349921 | 0 | 0.0530956 | 0.0274088 | 0 | 0.0349921 | 0 | 0.0196216 | 0.1424338 | 0.008712 | 0.2589805 | 0 | 0.0646659 | 0 | 0.1267459 | 0.0029058 | 0.024621 |
| TCGA-AG-A023-01A | 0.0789384 | 0.0345923 | 0.0633394 | 0.1940086 | 0 | 0.062624 | 0 | 0.1505716 | 0.1093167 | 0 | 0 | 0.006954 | 0.0229419 | 0.0362285 | 0.0147257 | 0.1539337 | 0.0065294 | 0 | 0 | 0.0652958 | 0 | 0 |
| TCGA-EI-6510-01A | 0 | 0.028788 | 0.0906336 | 0.1561624 | 0 | 0.347529 | 0 | 0.0904063 | 0.0543326 | 0 | 0 | 0.0298929 | 0.0190932 | 0.05013 | 0.0125257 | 0.0741486 | 0.0128197 | 0.0166723 | 0 | 0.0168658 | 0 | 0 |
| TCGA-AF-2691-01A | 0.0306342 | 0 | 0.0432164 | 0.1659944 | 0 | 0.1762086 | 0 | 0.0807226 | 0.0434676 | 0 | 0.0029799 | 0.0530245 | 0.0354863 | 0.064198 | 0.0436638 | 0.2160239 | 0.004468 | 0 | 0 | 0.0284899 | 0.0114218 | 0 |
| TCGA-F5-6813-01A | 0.0374616 | 0 | 0.0338145 | 0.1297278 | 0 | 0.1009996 | 0 | 0.080052 | 0.0404137 | 0 | 0 | 0.029766 | 0.002269 | 0.2742312 | 0.0970131 | 0.1665185 | 0 | 0 | 0.0013526 | 0.0063804 | 0 | 0 |
| TCGA-F5-6861-01A | 0.0275176 | 0 | 0.0517876 | 0.1041531 | 0 | 0.0567849 | 0 | 0.0450402 | 0.0310502 | 0 | 0 | 0.0360164 | 0 | 0.3460267 | 0.0572536 | 0.2284599 | 0 | 0 | 0.0159099 | 0 | 0 | 0 |
| TCGA-AF-A56K-01A | 0.0074835 | 0.0278636 | 0.114101 | 0.1513755 | 0 | 0.0187323 | 0.0605583 | 0.0984315 | 0 | 0 | 0.0360035 | 0.0057733 | 0.0591973 | 0.1956442 | 0.0159451 | 0.1506616 | 0.0145247 | 0 | 0 | 0.0428703 | 0.0008343 | 0 |
| TCGA-AG-4021-01A | 0 | 0.0087106 | 0.0109249 | 0.0188614 | 0 | 0.0327172 | 0.0332038 | 0.0112384 | 0.0135159 | 0 | 0.0194883 | 0 | 0.0282788 | 0.217811 | 0.0325038 | 0.3620729 | 0 | 0 | 0 | 0.1975195 | 0 | 0.0131535 |
| TCGA-AG-A01N-01A | 0 | 0.0469015 | 0.0713766 | 0.0246767 | 0 | 0.0585953 | 0.0311258 | 0.030376 | 0.0367889 | 0 | 0 | 0 | 0 | 0.4241088 | 0.0103423 | 0.1488687 | 0 | 0 | 0 | 0.0901954 | 0 | 0.0266439 |
| TCGA-DC-5869-01A | 0.0188653 | 0.0422292 | 0.008488 | 0.070153 | 0 | 0.2094096 | 0.0196923 | 0.0811373 | 0.0366773 | 0 | 0.0174019 | 0 | 0.0204005 | 0.0570235 | 0.048834 | 0.1026443 | 0.1416516 | 0.02714 | 0 | 0.0362421 | 0.06201 | 0 |
| TCGA-DY-A0XA-01A | 0.0294029 | 0.0132177 | 0.0884462 | 0.15097 | 0 | 0.171233 | 0 | 0.0786969 | 0.019193 | 0 | 0 | 0.0293134 | 0 | 0.2171294 | 0.0353498 | 0.1426515 | 0 | 0 | 0 | 0.0243963 | 0 | 0 |
| TCGA-AG-3594-01A | 0.0170509 | 0 | 0.0209094 | 0.2841708 | 0 | 0 | 0.002238 | 0.1683326 | 0.0257466 | 0 | 0 | 0.0699994 | 0 | 0.0496273 | 0.0918888 | 0.1808733 | 0 | 0 | 0.0004917 | 0 | 0 | 0.0886711 |
| TCGA-EF-5831-01A | 0.074645 | 0 | 0.0084747 | 0.0774336 | 0 | 0.0935773 | 0.0346365 | 0.0535402 | 0.0293933 | 0 | 0.0036553 | 0.0284943 | 0.0194264 | 0.1573173 | 0.0709402 | 0.310488 | 0.0195514 | 0 | 0 | 0.0184265 | 0 | 0 |
| TCGA-AG-3725-01A | 0.0007987 | 0.0049511 | 0.0343938 | 0.1504829 | 0 | 0.1183038 | 0.0761861 | 0.0880496 | 0.0623745 | 0 | 0 | 0.0214087 | 0.0207455 | 0.0608133 | 0.0635983 | 0.2033651 | 0.0010378 | 0 | 0 | 0.0934908 | 0 | 0 |
| TCGA-AG-3608-01A | 0.0159816 | 0.0061915 | 0.0324587 | 0.1660826 | 0 | 0.0688086 | 0.00489 | 0.129196 | 0.0389009 | 0 | 0 | 0.0497731 | 0.0510608 | 0 | 0.0996203 | 0.1986254 | 0.0410352 | 0 | 0 | 0.0920857 | 0.0052895 | 0 |
| TCGA-CI-6624-01C | 0.0616101 | 0 | 0.0040513 | 0.0585102 | 0 | 0.2122571 | 0 | 0.0725822 | 0.0827624 | 0 | 0 | 0.0144828 | 0 | 0.0959947 | 0.1007038 | 0.2031421 | 0.016641 | 0 | 0.0736428 | 0 | 0 | 0.0036194 |
| TCGA-CI-6620-01A | 0.0124222 | 0.0763146 | 0.0243969 | 0.1025449 | 0 | 0.2091981 | 0.0038024 | 0.0309525 | 0.0212166 | 0 | 0.0470605 | 0 | 0.0442069 | 0.1311973 | 0 | 0.1804435 | 0.0063061 | 0.0375414 | 0.0370848 | 0.0353112 | 0 | 0 |
| TCGA-CI-6621-01A | 0.0598066 | 0 | 0.0331624 | 0.0580257 | 0 | 0.1769157 | 0.0616104 | 0.0330346 | 0.0123707 | 0 | 0 | 0.019215 | 0 | 0.1418931 | 0.0764242 | 0.2629226 | 0 | 0 | 0.0293728 | 0 | 0 | 0.0352463 |
| TCGA-DY-A1DG-01A | 0 | 0.1032994 | 0 | 0.1630406 | 0 | 0.0267485 | 0 | 0.029806 | 0.0697616 | 0 | 0 | 0.0312941 | 0.0012976 | 0.3092531 | 0.0689701 | 0.1515079 | 0 | 0.0450212 | 0 | 0 | 0 | 0 |
| TCGA-AG-A02X-01A | 0.0966071 | 0 | 0.1389253 | 0.0711857 | 0 | 0.1505557 | 0.017714 | 0.0650903 | 0.0413275 | 0 | 0.0133602 | 0.0034488 | 0 | 0.1108245 | 0.0245093 | 0.2283454 | 0 | 0 | 0 | 0.0381063 | 0 | 0 |
| TCGA-AG-3587-01A | 0.0536953 | 0 | 0.0532344 | 0.1038086 | 0 | 0.1825975 | 0 | 0.1190792 | 0.0463187 | 0 | 0.0319194 | 0 | 0.0120192 | 0.0725094 | 0.0393731 | 0.2384931 | 0.0093011 | 0.0009038 | 0 | 0.0365303 | 0 | 0.000217 |
| TCGA-AG-3894-01A | 0.0504825 | 0 | 0.0482319 | 0.1379179 | 0 | 0.0988789 | 0 | 0.1129958 | 0.0075955 | 0 | 0.0417422 | 0 | 0.0299134 | 0.0398863 | 0.0325145 | 0.3642209 | 0 | 0 | 0.0103809 | 0.019847 | 0.0034229 | 0.0019691 |
| TCGA-AG-3999-01A | 0.0348352 | 0 | 0.0342596 | 0.0787251 | 0 | 0 | 0 | 0.1520548 | 0.0198279 | 0 | 0.0453438 | 0 | 0 | 0.143541 | 0.0201671 | 0.3453365 | 0 | 0 | 0.0033789 | 0.0877249 | 0 | 0.0348052 |
| TCGA-AF-A56N-01A | 0.1164647 | 0 | 0.1071209 | 0.0392304 | 0 | 0.1687515 | 0.0563688 | 0.0259632 | 0.0811784 | 0 | 0.025497 | 0 | 0.0321568 | 0.1486844 | 0.0125366 | 0.1515096 | 0.0020486 | 0.0004084 | 0 | 0.0320808 | 0 | 0 |
| TCGA-AG-A00C-01A | 0.0149775 | 0.0381915 | 0.1024497 | 0.1116624 | 0.0141701 | 0.0659051 | 0 | 0.2312586 | 0.0312841 | 0 | 0 | 0.0461547 | 0.0141672 | 0.1261081 | 0.0249064 | 0.1641253 | 0 | 0 | 0 | 0.0146393 | 0 | 0 |
| TCGA-EI-6885-01A | 0.0649102 | 0 | 0.0141191 | 0.0468494 | 0 | 0.1117565 | 0.0132696 | 0.0353263 | 0.0055283 | 0 | 0.030861 | 0 | 0.0035451 | 0.3150456 | 0.0498083 | 0.1843202 | 0 | 0.0155646 | 0 | 0.1090958 | 0 | 0 |
| TCGA-AG-3732-01A | 0.071914 | 0.1076147 | 0.0977732 | 0.0995684 | 0 | 0.20086 | 0 | 0.0745461 | 0.0834018 | 0 | 0 | 0.0112265 | 0.0017796 | 0.0188385 | 0.046886 | 0.0866408 | 0 | 0.0031305 | 0.0958198 | 0 | 0 | 0 |
| TCGA-AG-4007-01A | 0 | 0.02598 | 0.1463688 | 0.1612199 | 0 | 0.0516764 | 0 | 0.0556533 | 0.0952079 | 0 | 0 | 0.0150593 | 0.004449 | 0.0439305 | 0.0464404 | 0.3527049 | 0 | 0 | 0 | 0.0013097 | 0 | 0 |
| TCGA-AG-A016-01A | 0.0374286 | 0 | 0.1501263 | 0.1114642 | 0.0342455 | 0.0286347 | 0 | 0.0988355 | 0 | 0 | 0 | 0.1066659 | 0.0293651 | 0.032449 | 0.0033751 | 0.327257 | 0 | 0 | 0 | 0.0401531 | 0 | 0 |
| TCGA-DY-A1H8-01A | 0.023007 | 0.0629942 | 0 | 0.10048 | 0 | 0.1336481 | 0.0556547 | 0.1142288 | 0 | 0 | 0.1305362 | 0 | 0 | 0.2430665 | 0 | 0.0017295 | 0 | 0.0243473 | 0 | 0.1103077 | 0 | 0 |
| TCGA-AG-3575-01A | 0 | 0.0393511 | 0 | 0.1262321 | 0 | 0.0798385 | 0.0316652 | 0.0584231 | 0 | 0 | 0 | 0.0249223 | 0.0567901 | 0 | 0.0209574 | 0.2581401 | 0.0035864 | 0.0349867 | 0 | 0.1842135 | 0.0602764 | 0.0206172 |
| TCGA-AF-2693-01A | 0.0606353 | 0 | 0.0209206 | 0.1233781 | 0 | 0.2521048 | 0 | 0.0418729 | 0.0307848 | 0 | 0.0329305 | 0 | 0.0570291 | 0 | 0.0272364 | 0.142742 | 0.0378687 | 0.0237432 | 0 | 0.1374617 | 0.0112919 | 0 |
| TCGA-DC-6157-01A | 0 | 0.0102743 | 0.0612397 | 0.0538121 | 0 | 0.1916044 | 0 | 0.117949 | 0 | 0 | 0 | 0.0238802 | 0.0054849 | 0.0630601 | 0.1308179 | 0.2780356 | 0.0206001 | 0 | 0.0432417 | 0 | 0 | 0 |
| TCGA-AF-4110-01A | 0.0044413 | 0.0030721 | 0.1044473 | 0.1319819 | 0 | 0.1065307 | 0.004867 | 0.0784359 | 0.0488287 | 0 | 0.016651 | 0 | 0.0100523 | 0.164155 | 0.0539084 | 0.218928 | 0 | 0 | 0.0077107 | 0.0265942 | 0 | 0.0193953 |
| TCGA-AG-3878-01A | 0.1437775 | 0 | 0.0586039 | 0.2287171 | 0 | 0.022169 | 0.0220909 | 0.0783287 | 0.1089087 | 0 | 0.0723897 | 0 | 0.0331656 | 0.0141483 | 0.0304813 | 0.1004307 | 0.0145588 | 0 | 0 | 0.0718191 | 0.0004108 | 0 |
| TCGA-EI-6509-01A | 0 | 0.011192 | 0 | 0.0029734 | 0.1252397 | 0 | 0 | 0 | 0.0026057 | 0 | 0.0417297 | 0.001746 | 0 | 0.5889713 | 0.0058303 | 0.1962137 | 0 | 0 | 0 | 0.0234983 | 0 | 0 |
| TCGA-EI-6511-01A | 0 | 0.0144846 | 0.0887421 | 0.1410191 | 0 | 0.1334924 | 0 | 0.0573917 | 0.0544464 | 0 | 0 | 0.0245346 | 0.0088635 | 0.1782933 | 0.1071238 | 0.1629366 | 0 | 0 | 0.0006987 | 0.01755 | 0 | 0.0104232 |
| TCGA-EI-6881-01A | 0.0388044 | 0 | 0.0201422 | 0.0571687 | 0 | 0.2718744 | 0.0573687 | 0.0447376 | 0.0228001 | 0 | 0.0570506 | 0 | 0.0368746 | 0.0701908 | 0.0059824 | 0.110012 | 0 | 0.0385273 | 0 | 0.127136 | 0 | 0.0413301 |
| TCGA-AG-A008-01A | 0 | 0 | 0.0462659 | 0.0446065 | 0 | 0.4110825 | 0 | 0.0280273 | 0 | 0 | 0 | 0.109241 | 0.0102078 | 0.0102096 | 9.28E-06 | 0.2226727 | 0 | 0.0151212 | 0 | 0.1025561 | 0 | 0 |
| TCGA-AG-A015-01A | 0 | 0.0953367 | 0.0245041 | 0.0282834 | 0.0982882 | 0.144556 | 0 | 0.0277348 | 0.0468346 | 0 | 0.026805 | 0 | 0.0157399 | 0.1806532 | 0 | 0.0930847 | 0.0158372 | 0.0671488 | 0 | 0.1351935 | 0 | 0 |
| TCGA-AG-3892-01A | 0.0289983 | 0 | 0.0661899 | 0.1650517 | 0 | 0.0241212 | 0.0244926 | 0.1339246 | 0.0524431 | 0 | 0.0359763 | 0 | 0.0245053 | 0.0618971 | 0.0890393 | 0.125748 | 0.0094026 | 0.002741 | 0 | 0.110714 | 0.0062837 | 0.0384714 |
| TCGA-G5-6233-01A | 0.1037054 | 0 | 0.0385631 | 0.0346005 | 0 | 0.121134 | 0.0030757 | 0.0529923 | 0.0090233 | 0 | 0.0178447 | 0 | 0 | 0.2059596 | 0 | 0.2847297 | 0 | 0.0236941 | 0.073082 | 0.0315883 | 0 | 7.33E-06 |
| TCGA-AG-3898-01A | 0 | 0.0469859 | 0.0298632 | 0.1112837 | 0 | 0 | 0.0078418 | 0.1211232 | 0.0277474 | 0 | 0 | 0.026592 | 0.000369 | 0.0915366 | 0.1282941 | 0.2345422 | 0.0145263 | 0.0001146 | 0 | 0.0551812 | 0 | 0.1039986 |
| TCGA-AG-3578-01A | 0 | 0.0035102 | 0.0857931 | 0.0524606 | 0 | 0.1933432 | 0 | 0.0335405 | 0.0011335 | 0 | 0.0333187 | 0 | 0 | 0.2033236 | 0.0247466 | 0.2301802 | 0 | 0 | 0.0316878 | 0.0382691 | 0 | 0.0686929 |
| TCGA-DC-6682-01A | 0.0976528 | 0 | 0.0039303 | 0.164984 | 0 | 0.0574546 | 0 | 0.1153213 | 0.0702093 | 0 | 0 | 0.0616513 | 0 | 0.2187762 | 0.0796678 | 0.1158966 | 0 | 0 | 0 | 0.0144558 | 0 | 0 |
| TCGA-EI-6507-01A | 0.0254474 | 0 | 0.0215881 | 0.1816452 | 0 | 0.0733018 | 0.0301055 | 0.058387 | 0 | 0 | 0.0217971 | 0.0108616 | 0.0297903 | 0.158975 | 0.0498374 | 0.170596 | 0 | 0 | 0 | 0.1233712 | 0 | 0.0442964 |
| TCGA-F5-6702-01A | 0.0434357 | 0 | 0.0070095 | 0.0440685 | 0 | 0.1510803 | 0 | 0.0491662 | 0.0105679 | 0 | 0 | 0.0064226 | 0 | 0.1588602 | 0.0984651 | 0.3851512 | 0.011062 | 0 | 0.0325507 | 0 | 0 | 0.0021599 |
| TCGA-CL-5917-01A | 0.0199922 | 0.0273616 | 0 | 0.135175 | 0 | 0.1445168 | 0.0434983 | 0.0071707 | 0.018509 | 0 | 0.0222123 | 0 | 0.0858884 | 0.2002348 | 0 | 0.0195993 | 0.0308498 | 0.0049635 | 0 | 0.1760476 | 0.0639807 | 0 |
| TCGA-AG-3598-01A | 0.0242684 | 0 | 0.0130197 | 0.1619203 | 0 | 0 | 0.0792604 | 0.0948419 | 0.0218149 | 0 | 0.0179199 | 0 | 0 | 0.0775354 | 0.0939304 | 0.2677868 | 0.0065935 | 0 | 0 | 0.1296881 | 0 | 0.0114205 |
| TCGA-AG-3728-01A | 0.0451221 | 0.061323 | 0.0123737 | 0.15515 | 0 | 0.1423382 | 0.0108165 | 0.0293376 | 0.1426394 | 0 | 0.0169646 | 0.0263988 | 0.006918 | 0.1072764 | 0.0651755 | 0.1327451 | 0.0237781 | 0 | 0 | 0.0216431 | 0 | 0 |
| TCGA-DC-4749-01A | 0.0195079 | 0.066043 | 0.0599798 | 0.0656766 | 0 | 0.1908323 | 0.0106571 | 0.0679178 | 0.0153054 | 0 | 0.07799 | 0.0233175 | 0.0465348 | 0.1926066 | 0.0356334 | 0.0385057 | 0.017516 | 0 | 0 | 0.071976 | 0 | 0 |
| TCGA-AF-3400-01A | 0.0231629 | 0 | 0.0044195 | 0 | 0 | 0.0638397 | 0 | 0.0110495 | 0 | 0 | 0 | 0.0184672 | 0.0518348 | 0.1004493 | 0.0101278 | 0.2393806 | 0 | 0.0226772 | 0 | 0.316212 | 0.0102688 | 0.1281108 |
| TCGA-AF-2690-01A | 0.0529758 | 0 | 0.0123477 | 0.0746617 | 0 | 0.1757768 | 0 | 0.058776 | 0.0524513 | 0 | 0 | 0.0148066 | 0.0376628 | 0.0712855 | 0.0429453 | 0.3206275 | 0.0252242 | 0 | 0.0403362 | 0.0088184 | 0 | 0.0113042 |
| TCGA-EF-5830-01A | 0.0032347 | 0 | 0 | 0.1699653 | 0 | 0.1012838 | 0 | 0.0006327 | 0 | 0 | 0.0156928 | 0.1225316 | 0.0170974 | 0.0877776 | 0.03067 | 0.3659049 | 0 | 0.0198514 | 0.0653579 | 0 | 0 | 0 |
| TCGA-AF-3911-01A | 0.0534062 | 0 | 0.0358176 | 0.0779459 | 0 | 0.1638925 | 0 | 0.0671334 | 0 | 0 | 0.0060503 | 0.0084495 | 0 | 0.1571767 | 0.0621285 | 0.2978515 | 0 | 0 | 0 | 0.0376973 | 0.0257298 | 0.0067207 |
| TCGA-DC-6155-01A | 0 | 0.1347003 | 0.0312371 | 0.1264783 | 0.0279487 | 0.0222102 | 0 | 0.0364169 | 0.0326545 | 0 | 0.0164412 | 0.0285502 | 0 | 0.2822897 | 0.019841 | 0.1098297 | 0 | 0 | 0 | 0.1187687 | 0 | 0.0126337 |
| TCGA-DC-6681-01A | 0.0332263 | 0 | 0.1186826 | 0.0566559 | 0 | 0.239804 | 0 | 0.0349659 | 0.0525527 | 0 | 0 | 0.0052828 | 0 | 0.182654 | 0.0444181 | 0.21637 | 0 | 0 | 0.0147138 | 0.0006742 | 0 | 0 |
| TCGA-AF-A56L-01A | 0.0560463 | 0 | 0.0910867 | 0.1092392 | 0 | 0 | 0 | 0.1090953 | 0.0808853 | 0 | 0 | 0.0612975 | 0 | 0.1039063 | 0.0756855 | 0.2478974 | 0 | 4.68E-05 | 0.0648136 | 0 | 0 | 0 |
| TCGA-CI-6623-01B | 0.0264423 | 0 | 0.0424998 | 0.0826169 | 0 | 0.1037351 | 0.0337765 | 0.0270453 | 0.0181109 | 0 | 0 | 0.0250237 | 0.0396546 | 0.1543605 | 0.0490396 | 0.1865486 | 0 | 0.0038251 | 0 | 0.0140927 | 0 | 0.1932284 |
| TCGA-AH-6897-01A | 0 | 0.0297748 | 0.0537563 | 0.0778262 | 0 | 0.210175 | 0.0961926 | 0.0277967 | 0.0193529 | 0 | 0.1313069 | 0 | 0.0236546 | 0.0591819 | 0.0215033 | 0.1023199 | 0 | 0.0361272 | 0 | 0.1110318 | 0 | 0 |
| TCGA-AG-3580-01A | 0.0465456 | 0 | 0.048066 | 0.1039945 | 0 | 0.10215 | 0.0887353 | 0.1078615 | 0.0238411 | 0 | 0.021845 | 0 | 0 | 0.0611357 | 0.021862 | 0.1804419 | 0 | 0.0189193 | 0 | 0.1524515 | 0 | 0.0221507 |
| TCGA-AG-4022-01A | 0.0862947 | 0 | 0.0081484 | 0.0756408 | 0 | 0.1678271 | 0 | 0.0730533 | 0.0097175 | 0 | 0.01725 | 0.0002482 | 0.0122401 | 0.0990882 | 0.1260571 | 0.2447261 | 0.0449963 | 0 | 0.0285606 | 0 | 0.0061516 | 0 |
| TCGA-G5-6235-01A | 0.0519583 | 0.2299323 | 0 | 0.257985 | 0 | 0.0250799 | 0.0346877 | 0.0587181 | 0.0169836 | 0 | 0.0672273 | 0.0330366 | 0.0497435 | 0.0837829 | 0.0078989 | 0 | 0 | 0.0311344 | 0 | 0.0424769 | 0.0093546 | 0 |
| TCGA-AG-4005-01A | 0.0089816 | 0.0011946 | 0.0895521 | 0.0671106 | 0 | 0.2165659 | 0 | 0.0427537 | 0.0505824 | 0 | 0 | 0.0405057 | 0.0324603 | 0.0445541 | 0 | 0.2181716 | 0.0196246 | 0.0158517 | 0 | 0.1291823 | 0 | 0.0229088 |
| TCGA-AG-3583-01A | 0.0166638 | 0 | 0.0493025 | 0.1248332 | 0 | 0.226122 | 0 | 0.0631 | 0.0505497 | 0 | 0.0383483 | 0 | 0.0172392 | 0 | 0.0186301 | 0.1981441 | 0.0107211 | 0.0054447 | 0 | 0.145038 | 0.0251703 | 0.010693 |
| TCGA-AH-6547-01A | 0.0579429 | 0.1075713 | 0.0218838 | 0.1169241 | 0 | 0.1945769 | 0.00265 | 0.0436446 | 0.0195429 | 0 | 0.0205199 | 0 | 0 | 0.1017 | 0.0347557 | 0.2501291 | 0 | 0 | 0.0048315 | 0.0233274 | 0 | 0 |
| TCGA-EI-7002-01A | 0.0171202 | 0.0317629 | 0.027245 | 0.0761401 | 0 | 0.1047527 | 0.0137314 | 0.023624 | 0.0363151 | 0 | 0.0529772 | 0 | 0.0463165 | 0.1477103 | 0.037152 | 0.2813926 | 0.0327068 | 0 | 0 | 0.0710531 | 0 | 0 |
| TCGA-AG-A00Y-01A | 0.0403809 | 0.0683497 | 0.0494348 | 0.1356711 | 0 | 0 | 0.0419962 | 0.1326274 | 0.028366 | 0 | 0.0168562 | 0.0178593 | 0.0488218 | 0.1640577 | 0.1475314 | 0.0232822 | 0 | 0 | 0 | 0.0847653 | 0 | 0 |
| TCGA-EI-6514-01A | 0 | 0.0139027 | 0.0103664 | 0.0323965 | 0 | 0.0803946 | 0 | 0.0267281 | 0.03886 | 0 | 0 | 0.0453585 | 0 | 0.4401665 | 0.0260207 | 0.2676783 | 0 | 0 | 0.0063896 | 0.0117382 | 0 | 0 |
| TCGA-EI-6506-01A | 0 | 0.0052768 | 0.0850583 | 0.0638089 | 0 | 0.2264966 | 0 | 0.0295105 | 0.0485189 | 0 | 0 | 0.0263821 | 0.0074615 | 0.0783687 | 0.0335417 | 0.2990767 | 0 | 0.0127695 | 0.0559712 | 0.0259178 | 0 | 0.0018408 |
| TCGA-AG-A032-01A | 0.0449714 | 0 | 0.0973508 | 0.1108671 | 0 | 0.0926791 | 0 | 0.1749899 | 0.1269134 | 0 | 0 | 0.0962791 | 0.0114696 | 0.0543653 | 0.0324379 | 0.1459036 | 0 | 0.0090005 | 0.0027723 | 0 | 0 | 0 |
| TCGA-AG-3890-01A | 0.0170039 | 0.0328369 | 0.0672145 | 0.145066 | 0 | 0.2186787 | 0 | 0.1324672 | 0.057752 | 0 | 0 | 0.0317185 | 0.0141177 | 0.0121118 | 0.0218254 | 0.2104978 | 0 | 0.0132186 | 0 | 0.0254909 | 0 | 0 |
| TCGA-DY-A1DF-01A | 0 | 0.191508 | 0.0106914 | 0.018195 | 0.0580206 | 0.1058471 | 0 | 0.0186243 | 0.0385984 | 0 | 0.0730638 | 0.0375813 | 0.0768731 | 0.1158198 | 0 | 0.2049364 | 0.0030465 | 0.0095585 | 0 | 0.0286342 | 0.0067045 | 0.0022972 |
| TCGA-AG-A002-01A | 0.0337319 | 0.0032451 | 0 | 0.059828 | 0 | 0.1528413 | 0 | 0.0236195 | 0.0500012 | 0 | 0.012574 | 0.0575567 | 0.0517124 | 0.0211049 | 0 | 0.2186762 | 0 | 0.0168501 | 0 | 0.2838936 | 0 | 0.0143651 |
| TCGA-AG-3882-01A | 0.1054505 | 0 | 0.0779114 | 0.2591035 | 0 | 0 | 0.016154 | 0.0998971 | 0.1272221 | 0 | 0.031512 | 0.0009059 | 0.0440405 | 0.078688 | 0.0449754 | 0.0769559 | 0.0102424 | 0 | 0 | 0.0269413 | 0 | 0 |
| TCGA-CL-4957-01A | 0.1438832 | 0 | 0.0688426 | 0.0486906 | 0 | 0.1798601 | 0.046472 | 0.1043175 | 0.0162225 | 0 | 0 | 0.0222902 | 0.0065248 | 0.116625 | 0 | 0.1947941 | 0 | 0.0375579 | 0.0139194 | 0 | 0 | 0 |
| TCGA-F5-6811-01A | 0.0119575 | 0.0377438 | 0.0116765 | 0.0747759 | 0 | 0.1136558 | 0.03736 | 0.0464304 | 0.0440367 | 0 | 0.021307 | 0.0237444 | 0.0226484 | 0.2256394 | 0.0407573 | 0.2465009 | 0.0081891 | 0.0011071 | 0 | 0.0324696 | 0 | 0 |
| TCGA-AG-3896-01A | 0 | 0.0521725 | 0.0241939 | 0.0648676 | 0 | 0.0968748 | 0.0165736 | 0.0708407 | 0.0464215 | 0 | 0.1080365 | 0 | 0.0377393 | 0.0166462 | 0.0342496 | 0.3509308 | 0.0153758 | 0 | 0 | 0.0584597 | 0.0066176 | 0 |
| TCGA-G5-6641-01A | 0.0571764 | 0 | 0.2052591 | 0.0679161 | 0 | 0.2728538 | 0 | 0.0970766 | 0 | 0 | 0.0510775 | 0 | 0.0153488 | 0.100298 | 0.009944 | 0.0702937 | 0 | 0.0118839 | 0 | 0.040872 | 0 | 0 |
| TCGA-CI-6619-01B | 0.0399651 | 0 | 0.035443 | 0.0656985 | 0 | 0.159368 | 0.0031912 | 0.0719233 | 0.0062108 | 0 | 0.0042422 | 0.021856 | 0.0098476 | 0.0742231 | 0.0912362 | 0.222774 | 0 | 0.0307588 | 0.123105 | 0 | 0 | 0.0401574 |
| TCGA-AG-3602-01A | 0.0274594 | 0 | 0.0306611 | 0.123099 | 0 | 0.0786216 | 0 | 0.0976779 | 0 | 0.0581303 | 0 | 0.0350128 | 0 | 0.0028421 | 0.0291778 | 0.2531912 | 0.0657759 | 0.0001151 | 0 | 0.1376675 | 0.0408252 | 0.019743 |
| TCGA-AF-2692-01A | 0 | 0.0065505 | 0.0619858 | 0.0773494 | 0 | 0.1596205 | 0.0433423 | 0.0715986 | 0.015251 | 0 | 0.0994267 | 0 | 0.0171676 | 0.0916523 | 0.005835 | 0.1258013 | 0.0121995 | 0.0148019 | 0 | 0.1615307 | 0.0358869 | 0 |
| TCGA-DM-A288-01A | 0.0040503 | 0.0458335 | 0.0191397 | 0.0642434 | 0.0398925 | 0 | 0 | 0.0295672 | 0.0099986 | 0 | 0.0734225 | 0 | 0 | 0.5112007 | 0 | 0.046142 | 0 | 0 | 0 | 0.1565096 | 0 | 0 |
| TCGA-QL-A97D-01A | 0.0244139 | 0.0446025 | 0.0280048 | 0.2292222 | 0 | 0.0940647 | 0 | 0.1075729 | 0.0823403 | 0 | 0.0811587 | 0 | 0.0294083 | 0 | 0.035161 | 0.0523913 | 0.0133814 | 0.0155837 | 0 | 0.1579272 | 0.0047672 | 0 |
| TCGA-CM-6164-01A | 0.0031389 | 0.028825 | 0.0510126 | 0.1870957 | 0 | 0.0194453 | 0 | 0.1002803 | 0.0114292 | 0 | 0.0189895 | 0.0127172 | 0 | 0.2028456 | 0.109375 | 0.1715428 | 0 | 0 | 0 | 0.0740374 | 0 | 0.0092655 |
| TCGA-G4-6299-01A | 0.0714214 | 0 | 0.0149158 | 0.3048821 | 0 | 0 | 0 | 0.0920286 | 0.0295228 | 0 | 0.0083561 | 0.0152907 | 0.030259 | 0.0681842 | 0.052719 | 0.2941686 | 0.0149842 | 0 | 0 | 0.0032674 | 0 | 0 |
| TCGA-AA-3542-01A | 0.0116391 | 0 | 0.0865173 | 0.056165 | 0.0115837 | 0 | 0 | 0.0909469 | 0.0461419 | 0 | 0.0304404 | 0 | 0 | 0.2440296 | 0.0263757 | 0.2198461 | 0.0018147 | 0 | 0 | 0.1617072 | 0 | 0.0127924 |
| TCGA-AZ-4615-01A | 0.0334755 | 0 | 0.0229914 | 0.3491503 | 0 | 0.0265718 | 0.0114702 | 0.1016287 | 0.0418818 | 0 | 0 | 0.0552406 | 0 | 0.0261701 | 0.087219 | 0.2295605 | 0 | 0 | 0.0146401 | 0 | 0 | 0 |
| TCGA-AA-3549-01A | 0.0079815 | 0 | 0.042243 | 0.1460881 | 0 | 0.0137296 | 0.0096766 | 0.086344 | 0.0737075 | 0 | 0.0367594 | 0 | 0.0040333 | 0.2231917 | 0.0252499 | 0.0901936 | 0 | 0.0157995 | 0 | 0.211299 | 0 | 0.0137033 |
| TCGA-AY-4071-01A | 0.0467138 | 0 | 0.0415343 | 0.1283232 | 0 | 0.2149965 | 0 | 0.1053629 | 0.0899769 | 0 | 0.0222068 | 0 | 0.0095724 | 0.0209523 | 0.0567028 | 0.1830154 | 0.022899 | 0 | 0 | 0.0577439 | 0 | 0 |
| TCGA-CM-4752-01A | 0.088968 | 0 | 0.0368585 | 0.0594733 | 0 | 0.1014461 | 0 | 0.1769417 | 0.0490734 | 0 | 0.0184359 | 0.0113668 | 0 | 0.0933081 | 0.1225858 | 0.2026145 | 0.035358 | 0 | 0 | 0.00357 | 0 | 0 |
| TCGA-AA-3688-01A | 0.0035038 | 0.0162421 | 0.0945495 | 0.1260597 | 0 | 0.0737313 | 0.0255605 | 0.0346469 | 0.0938647 | 0 | 0.0056549 | 0 | 0.032321 | 0.0983749 | 0.0528104 | 0.2403474 | 0.026503 | 0 | 0 | 0.0758299 | 0 | 0 |
| TCGA-AA-3854-01A | 0.0518354 | 0 | 0.0562645 | 0.0452227 | 0 | 0.2716563 | 0 | 0.0145144 | 0.0292622 | 0 | 0.0072234 | 0.0095145 | 0.0113021 | 0 | 0.0191566 | 0.3252235 | 0.0009671 | 0.0078067 | 0 | 0.1367452 | 0 | 0.0133055 |
| TCGA-A6-3809-01A | 0.006298 | 0.00999 | 0.0015393 | 0.0966408 | 0 | 0.1099433 | 0.0459628 | 0.0682739 | 0.0081488 | 0 | 0 | 0.0413435 | 0 | 0.0912373 | 0.0868324 | 0.2417782 | 0 | 0 | 0 | 0.1644102 | 0 | 0.0276016 |
| TCGA-CM-6165-01A | 0.0505724 | 0 | 0.0205997 | 0.057102 | 0 | 0.0909838 | 0 | 0.0428228 | 0.0165712 | 0 | 0 | 0 | 0 | 0.3355109 | 0.0592092 | 0.2679767 | 0.0160129 | 0 | 0.0405294 | 0 | 0 | 0.0021089 |
| TCGA-CM-4751-01A | 0.0083883 | 0.0411682 | 0.0158431 | 0.2165131 | 0 | 0.1103171 | 0 | 0.1131649 | 0.0272189 | 0 | 0.0168945 | 0.0263958 | 0.0249179 | 0.0223467 | 0.1023524 | 0.218843 | 0.0029681 | 0.0016271 | 0.0394783 | 0.0115625 | 0 | 0 |
| TCGA-A6-5659-01A | 0.0405202 | 0.0131694 | 0.0010024 | 0.046662 | 0 | 0.0673606 | 0.0456042 | 0.0078991 | 0.0032291 | 0 | 0.0941165 | 0 | 0.0568858 | 0.3276315 | 0.062024 | 0.1093995 | 0.0164065 | 0 | 0 | 0.1080892 | 0 | 0 |
| TCGA-AA-3494-01A | 0.0448624 | 0 | 0.0656424 | 0.0627396 | 0 | 0.1312461 | 0.0339016 | 0.0910387 | 0.0379769 | 0 | 0 | 0.0306507 | 0 | 0.0245651 | 0.0106146 | 0.3177441 | 0 | 0.0202699 | 0 | 0.0949462 | 0 | 0.0338016 |
| TCGA-CM-4750-01A | 0.0663075 | 0.0066367 | 0.045354 | 0.2469112 | 0 | 0 | 0.0001369 | 0.20483 | 0.0780253 | 0 | 0 | 0.0058377 | 0.0214238 | 0.1122916 | 0.0755745 | 0.0556065 | 0.0154913 | 0 | 0 | 0.0617293 | 0.0038437 | 0 |
| TCGA-CM-6161-01A | 0.0119345 | 0 | 0.0198114 | 0.1283748 | 0 | 0.2604356 | 0.048761 | 0.0644143 | 0.0075389 | 0 | 0.0177637 | 0 | 0.0215066 | 0.0276254 | 0.0172593 | 0.2924493 | 0.0480616 | 0.0082677 | 0 | 0.0155687 | 0.0102272 | 0 |
| TCGA-NH-A50U-01A | 0.0023762 | 0.0362042 | 0 | 0.0290883 | 0 | 0 | 0 | 0.0128398 | 0.0735962 | 0 | 0.1050789 | 0 | 0.0068008 | 0.3396997 | 0.0054935 | 0.1524871 | 0 | 0 | 0 | 0.2363353 | 0 | 0 |
| TCGA-DM-A0XF-01A | 0.0200966 | 0 | 0.0622772 | 0.0379866 | 0 | 0.0917078 | 0 | 0.1347046 | 0.0416955 | 0 | 0 | 0.0334757 | 0.0001454 | 0.1424789 | 0.1912356 | 0.184506 | 0.0103259 | 0 | 0.0493642 | 0 | 0 | 0 |
| TCGA-AA-3529-01A | 0 | 0.0672458 | 0.01003 | 0.0923541 | 0 | 0.0782161 | 0.0695974 | 0 | 0.0592105 | 0 | 0.1062466 | 0 | 0.0633954 | 0.0262128 | 0 | 0.2143871 | 0.0037812 | 0.007139 | 0.0672435 | 0.1260484 | 0.0022405 | 0.0066516 |
| TCGA-AA-3949-01A | 0.0288536 | 0 | 0.0032353 | 0.1690596 | 0 | 0.0736012 | 0.0578034 | 0.0874419 | 0.0213919 | 0 | 0.0142228 | 0.0234035 | 0.0147057 | 0 | 0.0769945 | 0.1725017 | 0.0204939 | 0.0115522 | 0 | 0.1960277 | 0 | 0.028711 |
| TCGA-AA-3848-01A | 0.0196729 | 0.0289001 | 0.1007825 | 0.1487499 | 0 | 0.0968547 | 0.0307678 | 0.0184387 | 0.0711841 | 0 | 0.0600861 | 0.0723719 | 0.0628998 | 0.1579989 | 0.0154831 | 0.0839681 | 0.0185589 | 0 | 0.0044252 | 0.0080337 | 0.0008234 | 0 |
| TCGA-CA-6715-01A | 0 | 0.0018774 | 0.0809722 | 0.0381969 | 0 | 0.0935743 | 0 | 0.0306928 | 0.0325957 | 0 | 0.0277068 | 0.0089042 | 0.0043604 | 0.4069887 | 0.0097543 | 0.1261003 | 0 | 0 | 0 | 0.138276 | 0 | 0 |
| TCGA-AA-3818-01A | 0 | 0.0517057 | 0.014873 | 0.2446647 | 0 | 0.0289475 | 0 | 0.1299483 | 0.0104513 | 0 | 0 | 0.0433576 | 0 | 0.05449 | 0.0616396 | 0.324951 | 0.0016964 | 0.0039305 | 0 | 0.0271979 | 0 | 0.0021467 |
| TCGA-AA-3710-01A | 0.0134297 | 0 | 0.0074454 | 0.2388614 | 0 | 0 | 0.0688647 | 0.1220167 | 0.0189119 | 0 | 0.0043464 | 0.0125823 | 0.0027816 | 0 | 0.0631424 | 0.2067449 | 0.0184669 | 0.0059823 | 0 | 0.1466452 | 0 | 0.0697782 |
| TCGA-AA-3950-01A | 0 | 0 | 0.001813 | 0.0800848 | 0 | 0.09676 | 0.0253287 | 0.0192858 | 0.0123425 | 0 | 0.02392 | 0.002119 | 0.0440379 | 0.1468042 | 0.0523168 | 0.3187951 | 0 | 0 | 0 | 0.1296793 | 0 | 0.0467131 |
| TCGA-AA-A00N-01A | 0.0059598 | 0.018804 | 0.0008309 | 0.0727506 | 0 | 0.1150608 | 0 | 0.0150114 | 0.0333278 | 0 | 0.0309003 | 0 | 0 | 0.269262 | 0.0053221 | 0.2271753 | 0.0036273 | 0 | 0 | 0.1727608 | 0 | 0.029207 |
| TCGA-A6-6138-01A | 0.0253591 | 0 | 0.0166822 | 0.1469325 | 0 | 0.0734197 | 0.0254128 | 0.068189 | 0.0402819 | 0 | 0 | 0.0262877 | 0 | 0.1614249 | 0.1006002 | 0.2930633 | 0.004618 | 0 | 0.0144483 | 0 | 0 | 0.0032804 |
| TCGA-D5-6538-01A | 0 | 0.0328911 | 0.1004651 | 0.1207066 | 0.1461316 | 0 | 0 | 0.0206182 | 0 | 0 | 0.0999003 | 0 | 0.0226388 | 0.0693296 | 0 | 0.2894098 | 0 | 0.0055393 | 0 | 0.0923697 | 0 | 0 |
| TCGA-CM-5341-01A | 0.0150354 | 0 | 0.0033899 | 0.0293722 | 0 | 0.0676513 | 0 | 0.0628174 | 0.0080919 | 0 | 0 | 0.0123299 | 0.0092704 | 0.0758743 | 0.0419746 | 0.4013925 | 0.0485094 | 0.0291098 | 0 | 0.1790383 | 0 | 0.0161427 |
| TCGA-AA-3715-01A | 0.0469728 | 0.0010823 | 0.0121523 | 0.1046534 | 0 | 0.1021553 | 0.0213726 | 0.0368123 | 0.033007 | 0 | 0.0733737 | 0 | 0 | 0.1942345 | 0.0944458 | 0.1341193 | 0.0022201 | 0 | 0 | 0.1128598 | 0 | 0.0305388 |
| TCGA-AA-3506-01A | 0.0273644 | 0 | 0.0299271 | 0.1426004 | 0 | 0.1506011 | 0 | 0.040539 | 0.0225284 | 0 | 0.0604405 | 0 | 0.046796 | 0.0880779 | 0.0325696 | 0.209944 | 0.0020652 | 0.00699 | 0 | 0.1395564 | 0 | 0 |
| TCGA-AA-3673-01A | 0.0781314 | 0 | 0.0240155 | 0.1002827 | 0 | 0.076539 | 0.0142751 | 0.0352513 | 0.0581289 | 0 | 0.0666157 | 0 | 0.0373051 | 0.0793071 | 0.0814492 | 0.2164641 | 0.0397215 | 0 | 0.0438065 | 0.0487069 | 0 | 0 |
| TCGA-AU-3779-01A | 0.0169809 | 0 | 0.0359043 | 0.0268589 | 0 | 0.2452899 | 0 | 0.07061 | 0.0901274 | 0 | 0.0286568 | 0 | 0.0006237 | 0.2165108 | 0.0206155 | 0.1579475 | 0 | 0.0252667 | 0 | 0.0504814 | 0 | 0.0141261 |
| TCGA-CK-5916-01A | 0.0493047 | 0 | 0 | 0.1480245 | 0 | 0.0574556 | 0.0172114 | 0.1091298 | 0.0223497 | 0 | 0 | 0.0772329 | 0 | 0.0443344 | 0.103501 | 0.3201034 | 0.0202107 | 0 | 0.0190483 | 0 | 0 | 0.0120935 |
| TCGA-QG-A5Z1-01A | 0.0096379 | 0.0147195 | 0.0259277 | 0.0093744 | 0 | 0.1073335 | 0 | 0.0179115 | 0.0130611 | 0 | 0.0241274 | 0 | 0 | 0.4208907 | 0.013933 | 0.3420096 | 0 | 0 | 0 | 0.0010737 | 0 | 0 |
| TCGA-AD-6890-01A | 0.0084284 | 0.0228119 | 0.0821563 | 0.1466884 | 0 | 0.0576378 | 0.1155465 | 0.0302869 | 0 | 0 | 0.0636911 | 0 | 0 | 0.2566021 | 0.0838508 | 0.0925562 | 0.0027864 | 0 | 0 | 0.0369573 | 0 | 0 |
| TCGA-A6-2685-01A | 0.0806297 | 0 | 0.0304321 | 0.0289852 | 0 | 0.1178346 | 0 | 0.0725123 | 0.0142198 | 0 | 0 | 0.0057554 | 0.008173 | 0.1141508 | 0.0300641 | 0.3940221 | 0.0052334 | 0 | 0.0003948 | 0.0481149 | 0.0306238 | 0.018854 |
| TCGA-AA-A00W-01A | 0.0247742 | 0.015909 | 0.1281021 | 0.1697003 | 0 | 0 | 0 | 0.1688928 | 0.1104675 | 0 | 0.0739522 | 0.0034612 | 0.0311753 | 0.0265155 | 0 | 0.1027928 | 0 | 0.0609807 | 0 | 0.0832763 | 0 | 0 |
| TCGA-AA-A00Z-01A | 0.0469065 | 0 | 0.1022258 | 0.1080569 | 0 | 0.0453564 | 0 | 0.088778 | 0.0955651 | 0 | 0 | 0.0202587 | 0.0094104 | 0.0544186 | 0.132048 | 0.2601234 | 0 | 0.002635 | 0.0342172 | 0 | 0 | 0 |
| TCGA-A6-2686-01A | 0.0521826 | 0 | 0.017295 | 0.2180742 | 0 | 0.1255181 | 0.047118 | 0.0631218 | 0 | 0 | 0.0087154 | 0.0135398 | 0.0060949 | 0 | 0.0733583 | 0.2504969 | 0.0251002 | 0 | 0 | 0.0808132 | 0.0048558 | 0.0137157 |
| TCGA-CA-5255-01A | 0.129326 | 0 | 0.0443047 | 0.1092632 | 0 | 0.1006083 | 0 | 0.1219842 | 0.0644774 | 0 | 0 | 0.0384666 | 0.0072032 | 0.0425379 | 0.0831573 | 0.129633 | 0.0078269 | 0.0744024 | 0.0347029 | 0.012106 | 0 | 0 |
| TCGA-A6-A5ZU-01A | 0.081624 | 0 | 0.0274782 | 0.1147728 | 0 | 0 | 0 | 0.0910282 | 1.79E-05 | 0 | 0.0300595 | 0 | 0 | 0.206552 | 0.0715475 | 0.3514582 | 0.0011335 | 0 | 0.024196 | 0 | 0 | 0.0001321 |
| TCGA-AZ-4614-01A | 0.0149029 | 0.0523009 | 0 | 0.3259621 | 0 | 0 | 0 | 0.0897489 | 0.0315406 | 0 | 0.0336255 | 0 | 0.0257923 | 0.1354302 | 0.1137099 | 0 | 0 | 0.0114366 | 0 | 0.1580338 | 0.0075163 | 0 |
| TCGA-NH-A50V-01A | 0.0515543 | 0 | 0.0452308 | 0.0955326 | 0 | 0.1485951 | 0 | 0.0599489 | 0.1140267 | 0 | 0.0370827 | 0 | 0.0410262 | 0 | 0.0402917 | 0.1645916 | 0.030622 | 0.0124537 | 0 | 0.150534 | 0.0085098 | 0 |
| TCGA-QG-A5YX-01A | 0 | 0.0359221 | 0.1126229 | 0.045523 | 0 | 0.1885766 | 0 | 0.0782989 | 0.0164795 | 0 | 0.012749 | 0 | 0.0035538 | 0.0941799 | 0.0087567 | 0.3137772 | 0 | 0.0170913 | 0 | 0.0724692 | 0 | 0 |
| TCGA-A6-2677-01A | 0.1063494 | 0 | 0.0398876 | 0.0908249 | 0 | 0.201075 | 0 | 0.0622109 | 0.0122781 | 0 | 0.0049998 | 0 | 0.0261533 | 0.094488 | 0.0775159 | 0.1882034 | 0.0287003 | 0 | 0 | 0.0673137 | 0 | 0 |
| TCGA-AZ-6608-01A | 0.0965038 | 0 | 0.1252949 | 0.022987 | 0 | 0.2358518 | 0 | 0.0895139 | 0.0249049 | 0 | 0.0314041 | 0.0119744 | 0.020296 | 0.156227 | 0 | 0.0989518 | 0 | 0.0193307 | 0 | 0.0667597 | 0 | 0 |
| TCGA-CA-6717-01A | 0.0705412 | 0.0162326 | 0.0190303 | 0.1008776 | 0 | 0.1464765 | 0 | 0.0610907 | 0.0652583 | 0 | 0 | 0.0306548 | 0.0260475 | 0.0923346 | 0.0555194 | 0.2139065 | 0.0255915 | 0 | 0.0691291 | 0.0073093 | 0 | 0 |
| TCGA-AA-3527-01A | 0.0123164 | 0 | 0.0279051 | 0.146766 | 0 | 0 | 0 | 0.0915768 | 0.0614401 | 0 | 0.0331675 | 0 | 0.0052267 | 0.4047878 | 0.0502498 | 0.1040374 | 0.0060417 | 0 | 0 | 0.0564849 | 0 | 0 |
| TCGA-CM-4746-01A | 0 | 0.0207421 | 0 | 0.2223083 | 0 | 0.1393836 | 0 | 0.0320954 | 0.113104 | 0 | 0 | 0.0190302 | 0.0262103 | 0.175712 | 0.0323163 | 0.0571389 | 0 | 0.0136916 | 0 | 0.1482675 | 0 | 0 |
| TCGA-D5-6539-01A | 0.0333453 | 0 | 0.1277237 | 0.0731475 | 0 | 0.1762358 | 0 | 0.0147869 | 0.0247895 | 0 | 0 | 0.0471955 | 0.0219753 | 0 | 0.0269169 | 0.3612515 | 0.0289108 | 0.0187 | 0.0450212 | 0 | 0 | 0 |
| TCGA-5M-AAT6-01A | 0.0577756 | 0 | 0.046815 | 0.1446042 | 0 | 0.0473283 | 0.0225101 | 0.1319782 | 0.0102193 | 0 | 0 | 0.021104 | 0.0045711 | 0.1010472 | 0.1190981 | 0.2712833 | 0.0070122 | 0 | 0.0144386 | 0 | 0 | 0.0002148 |
| TCGA-A6-4105-01A | 0.0329515 | 0 | 0.0150976 | 0.1424383 | 0 | 0.1167529 | 0.0229151 | 0.0918736 | 0.0228224 | 0 | 0.042652 | 0 | 0.0115052 | 0.0741915 | 0.0853553 | 0.2445599 | 0 | 0 | 0 | 0.0838201 | 0 | 0.0130646 |
| TCGA-F4-6569-01A | 0.0901192 | 0 | 0.0124637 | 0.0686167 | 0 | 0.0746988 | 0 | 0.011034 | 0.0283956 | 0 | 0.0089776 | 0.0053814 | 0 | 0.3617951 | 0.0247205 | 0.2648085 | 0 | 0 | 0.0397972 | 0.0091916 | 0 | 0 |
| TCGA-AA-A00R-01A | 0.0232325 | 0 | 0.0014384 | 0.2275025 | 0 | 0 | 0.1188603 | 0.1102171 | 0.0210339 | 0 | 0.0382969 | 0.0463465 | 0.025881 | 0.072701 | 0.112897 | 0.1373015 | 0.0173843 | 0 | 0 | 0.046907 | 0 | 0 |
| TCGA-CM-4743-01A | 0 | 0.0492027 | 0.0333115 | 0.1624647 | 0 | 0 | 0 | 0.1544127 | 0.039712 | 0 | 0 | 0.1528637 | 0.0080215 | 0.0150297 | 0.171586 | 0.1477699 | 0.0255881 | 0.0120638 | 0 | 0.0235463 | 0 | 0.0044273 |
| TCGA-CK-6747-01A | 0.1008047 | 0 | 0.1950891 | 0.088712 | 0 | 0.2064376 | 0 | 0.0332816 | 0.0461353 | 0 | 0.0117735 | 0 | 0.0140984 | 0.0029024 | 0.094332 | 0.1531856 | 0.0532479 | 0 | 0 | 0 | 0 | 0 |
| TCGA-D5-6533-01A | 0.0378946 | 0 | 0.0383086 | 0.0374369 | 0 | 0.1927971 | 0 | 0.0610367 | 0.0081579 | 0 | 0.0156541 | 0.0080833 | 0 | 0.1861907 | 0.0865571 | 0.2934024 | 0 | 0.0104043 | 0 | 0.0240763 | 0 | 0 |
| TCGA-A6-2672-01B | 0 | 0.0485624 | 0 | 0 | 0.0940636 | 0.2105611 | 0 | 0.1109976 | 0 | 0 | 0.0663485 | 0.0546978 | 0.0339986 | 0.0375186 | 0.1196994 | 0.0784966 | 0.0085936 | 0.0363867 | 0 | 0.0729586 | 0 | 0.0271169 |
| TCGA-QG-A5Z2-01A | 0.2796566 | 0 | 0.0001754 | 0.2039591 | 0 | 0.152018 | 0 | 0.1157691 | 0.030235 | 0 | 0.0382182 | 0 | 0.0185878 | 0.0204891 | 0.0489149 | 0.0689025 | 0 | 0.0012122 | 0 | 0.021862 | 0 | 0 |
| TCGA-A6-6780-01A | 0.0141291 | 0 | 0.00696 | 0.1341479 | 0 | 0.0211898 | 0.1043135 | 0.0495642 | 0.0210856 | 0 | 0.0124347 | 0.0419953 | 0.0057318 | 0.1096416 | 0.0909798 | 0.2301352 | 0.0218522 | 0 | 0 | 0.1331682 | 0 | 0.0026714 |
| TCGA-F4-6459-01A | 0.0289988 | 0.0444696 | 0 | 0.0821022 | 0 | 0.1243635 | 0.0141986 | 0.0432519 | 0.0423269 | 0 | 0.0286186 | 0 | 0.0256753 | 0.1656609 | 0.0217407 | 0.2344492 | 0.0274671 | 0 | 0 | 0.1011762 | 0.0155005 | 0 |
| TCGA-AA-3970-01A | 0.1145023 | 0 | 0.0323901 | 0.0982073 | 0 | 0.0904863 | 0.0253783 | 0.1170092 | 0.0251272 | 0 | 0.035913 | 0 | 0.0313209 | 0.0252898 | 0.0247264 | 0.2393917 | 0.0049416 | 0 | 0 | 0.13255 | 0.0027658 | 0 |
| TCGA-NH-A8F7-01A | 0 | 0.0154307 | 0.0589416 | 0.0135817 | 0.19895 | 0.0879734 | 0.0086659 | 0 | 0 | 0 | 0.072661 | 0 | 0.0004103 | 0.0926437 | 0.0099922 | 0.1881941 | 0 | 0.0112158 | 0 | 0.2074443 | 0 | 0.0338953 |
| TCGA-G4-6321-01A | 0.1323115 | 0 | 0 | 0.2796031 | 0 | 0.2135739 | 0 | 0.1236236 | 0.0055304 | 0 | 0.0239275 | 0.0329515 | 0.0339395 | 0.0287221 | 0.0112096 | 0.0627566 | 0.0180341 | 0.010136 | 0.0033865 | 0.0162356 | 0.0040585 | 0 |
| TCGA-AY-6196-01A | 0.0231085 | 0.0495098 | 0.031008 | 0.1078105 | 0 | 0.1838298 | 0 | 0.1059689 | 0.0430951 | 0 | 0 | 0.0151987 | 0.06646 | 0.0134171 | 0.0457701 | 0.2818435 | 0.0073462 | 0 | 0 | 0.024435 | 0 | 0.0011987 |
| TCGA-AA-3524-01A | 0.0486177 | 0 | 0.0144292 | 0.249342 | 0 | 0 | 0.0038303 | 0.0847521 | 0.0549071 | 0 | 0 | 0.0587825 | 0 | 0.009135 | 0.0359675 | 0.2651824 | 0.0123251 | 0 | 0 | 0.1343196 | 0.005698 | 0.0227116 |
| TCGA-DM-A1D0-01A | 0.0089263 | 0.0329483 | 0 | 0 | 0 | 0.0710296 | 0 | 0.0228143 | 0.0137018 | 0 | 0.0426063 | 0 | 0 | 0.6352862 | 0.0137897 | 0.056824 | 0 | 0 | 0 | 0.1020735 | 0 | 0 |
| TCGA-G4-6307-01A | 0.0930634 | 0.1237457 | 0 | 0.0596597 | 0.0264261 | 0.0919055 | 0.0570291 | 0.0304773 | 0.008132 | 0 | 0.1666161 | 0 | 0.0037797 | 0.189611 | 0.0316633 | 0.0132618 | 0.033643 | 0 | 0 | 0.0709862 | 0 | 0 |
| TCGA-5M-AATE-01A | 0.0376786 | 0.0100157 | 0.1521123 | 0.0763503 | 0 | 0.1719822 | 0 | 0.1032802 | 0 | 0 | 0.0627228 | 0 | 0.0120477 | 0.0931734 | 0.016876 | 0.2083439 | 0.0016511 | 0.0040686 | 0 | 0.0496973 | 0 | 0 |
| TCGA-AA-3697-01A | 0.007033 | 0.0639265 | 0.0803489 | 0.0828866 | 0.0017727 | 0.0100805 | 0.084856 | 0.0420363 | 0.031747 | 0 | 0.0422794 | 0 | 0 | 0.0719859 | 0.0450475 | 0.2564858 | 0.0040421 | 0.0255007 | 0 | 0.1499712 | 0 | 0 |
| TCGA-NH-A6GA-01A | 0 | 0.0628322 | 0.0331615 | 0.2097415 | 0 | 0.1037732 | 0.0263199 | 0.0154795 | 0.0654121 | 0 | 0.0606052 | 0 | 0.0348174 | 0.1133685 | 0.0007173 | 0.1527848 | 0.0608406 | 0 | 0 | 0.0601464 | 0 | 0 |
| TCGA-AY-6197-01A | 0.0413071 | 0 | 0.0640983 | 0.0537591 | 0 | 0.2094968 | 0.0682777 | 0.0342557 | 0.0015803 | 0 | 0.0777394 | 0 | 0 | 0.1250312 | 0.0236812 | 0.1294631 | 0 | 0.0060424 | 0 | 0.1514942 | 0 | 0.0137737 |
| TCGA-AA-3672-01A | 0.0373418 | 0 | 0.0272306 | 0.2554009 | 0 | 0 | 0.0725998 | 0.09272 | 0.0484309 | 0 | 0.0680642 | 0.0013639 | 0.0319853 | 0.0155178 | 0.0826998 | 0.122453 | 0.0178743 | 0 | 0 | 0.124814 | 0 | 0.0015039 |
| TCGA-AA-3684-01A | 0.0723092 | 0 | 0.0062318 | 0.1040309 | 0 | 0.087665 | 0 | 0.1562917 | 0.1070316 | 0 | 0.0106714 | 0.013186 | 0.0238573 | 0.1055505 | 0.023687 | 0.2050995 | 0.0216425 | 0 | 0 | 0.0344773 | 0 | 0.0282684 |
| TCGA-A6-2684-01C | 0 | 0.026587 | 0 | 0.0095862 | 0.0658869 | 0.1700341 | 0 | 0.0166285 | 0 | 0 | 0.0373963 | 0.0141205 | 0 | 0.0695085 | 0.1649438 | 0.2545909 | 0 | 0.0328894 | 0 | 0.1075142 | 0.0226823 | 0.0076314 |
| TCGA-AZ-4323-01A | 0.1989803 | 0.0577982 | 0.0212051 | 0.2441207 | 0 | 0.0795566 | 0 | 0.1548695 | 0.0594517 | 0 | 0 | 0 | 0 | 0.0459805 | 0.0337042 | 0.0964978 | 0 | 0 | 0.0078355 | 0 | 0 | 0 |
| TCGA-AA-3971-01A | 0.1491009 | 0 | 0.2008073 | 0.1221808 | 0 | 0.0564209 | 0 | 0.0379078 | 0.057745 | 0 | 0 | 0.038227 | 0 | 0.0026369 | 0.0335547 | 0.2367952 | 0.0016388 | 0 | 0.035648 | 0.0273367 | 0 | 0 |
| TCGA-AA-3989-01A | 0.0160199 | 0.0232104 | 0.0556238 | 0.1876793 | 0 | 0.0532186 | 0.0202785 | 0.0608306 | 0.0340121 | 0 | 0.0442475 | 0.0130936 | 0.0664137 | 0.0154493 | 0.075912 | 0.2470564 | 0.0214195 | 0 | 0 | 0.0655347 | 0 | 0 |
| TCGA-AA-A00D-01A | 0.0197941 | 0.0097833 | 0.0375634 | 0.2189026 | 0 | 0 | 0.0273778 | 0.0886283 | 0.0671083 | 0 | 0 | 0.0787918 | 0.0263607 | 0 | 0.1176041 | 0.2709859 | 0.0163524 | 0 | 0.0047295 | 0.0132975 | 0 | 0.0027202 |
| TCGA-CM-6171-01A | 0.0483053 | 0 | 0.0262546 | 0.0447595 | 0 | 0.1847445 | 0 | 0.0285084 | 0 | 0 | 0 | 0.0627388 | 0 | 0.0687591 | 0.0716139 | 0.2261175 | 0 | 0 | 0 | 0.1288689 | 0 | 0.1093295 |
| TCGA-A6-2679-01A | 0 | 0.064097 | 0.0886951 | 0.2401956 | 0 | 0.0153789 | 0 | 0.1681141 | 0.14343 | 0 | 0 | 0.0758517 | 0.0139241 | 0.0936559 | 0.029127 | 0.0557141 | 0 | 0.0001001 | 0 | 0.0117164 | 0 | 0 |
| TCGA-A6-2674-01A | 0.0171015 | 0 | 0.0127992 | 0.0260415 | 0 | 0.08334 | 0 | 0.0385355 | 0.0052692 | 0 | 0 | 0.0265084 | 0.0047306 | 0.0843859 | 0.0145707 | 0.1838945 | 0.000957 | 0 | 0 | 0.3919467 | 0.0126097 | 0.0973097 |
| TCGA-NH-A5IV-01A | 0.195119 | 0 | 0.0841897 | 0.1402182 | 0 | 0.0479048 | 0 | 0.0475465 | 0.00783 | 0 | 0 | 0.1015048 | 0.0330686 | 0.0448749 | 0.0208264 | 0.2769172 | 0 | 0 | 0 | 0 | 0 | 0 |
| TCGA-A6-5664-01A | 0.0672681 | 0 | 0.0207213 | 0.1612913 | 0 | 0.1184784 | 0 | 0.0658441 | 0.0072704 | 0 | 0.0294149 | 0 | 0.0468893 | 0.0098166 | 0.0222405 | 0.3761224 | 0 | 0.0215995 | 0.0294569 | 0.0125651 | 0 | 0.0110213 |
| TCGA-AZ-4315-01A | 0.0344259 | 0 | 0.0268327 | 0.1526711 | 0 | 0.0206129 | 0.0196506 | 0.126666 | 0 | 0 | 0.0451043 | 0.0078339 | 0.0095223 | 0.0047589 | 0.1907117 | 0.3292629 | 0.0145114 | 0 | 0 | 0.0174355 | 0 | 0 |
| TCGA-F4-6806-01A | 0.1805506 | 0 | 0.0629302 | 0.2249976 | 0 | 0.0339608 | 0 | 0.1194041 | 0.0727162 | 0 | 0 | 0.0436506 | 0.026685 | 0.0580702 | 0.0368244 | 0.0966727 | 0.0015979 | 0.0076524 | 0 | 0.0342873 | 0 | 0 |
| TCGA-AA-3510-01A | 0.0132161 | 0.0182411 | 0.03401 | 0.0603385 | 0 | 0.0757513 | 0.0215697 | 0.0449561 | 0.0243805 | 0 | 0.0456071 | 0 | 0.0409057 | 0.0492857 | 0.0920778 | 0.3425242 | 0.0165534 | 0 | 0 | 0.1205828 | 0 | 0 |
| TCGA-AY-A71X-01A | 0 | 0.090196 | 0.0933304 | 0.1030078 | 0.0121346 | 0.1470199 | 0 | 0.0325576 | 0.0341964 | 0 | 0.0416797 | 0 | 0.021659 | 0.1795257 | 0.0005019 | 0.1207786 | 0.0006676 | 0.007778 | 0 | 0.1149669 | 0 | 0 |
| TCGA-AA-3488-01A | 0 | 0 | 0.0558327 | 0.1266691 | 0 | 0.0245883 | 0 | 0.0460851 | 0.0472904 | 0 | 0 | 0.0502081 | 0.0466639 | 0.0673392 | 0.0437481 | 0.2760713 | 0 | 0 | 0 | 0.1788285 | 0 | 0.0366752 |
| TCGA-DM-A28F-01A | 0.0115622 | 0.0576155 | 0.0721522 | 0.1449829 | 0 | 0.0856971 | 0 | 0.1659777 | 0.0789196 | 0 | 0.001509 | 0 | 0.022413 | 0.0788835 | 0.1438697 | 0.0900228 | 0.0045735 | 0 | 0 | 0.0418212 | 0 | 0 |
| TCGA-G4-6293-01A | 0 | 0.0090788 | 0.098807 | 0.1360458 | 0 | 0.3381319 | 0 | 0.0447355 | 0.0688715 | 0 | 0.0050698 | 0.0158266 | 0.0084874 | 0.0623499 | 0.0683109 | 0.1025195 | 0 | 0 | 0.0181408 | 0.0163083 | 0 | 0.0073163 |
| TCGA-5M-AAT4-01A | 0 | 0.0740488 | 0.1000497 | 0.1895085 | 0 | 0 | 0 | 0.1323222 | 0 | 0 | 0.0065975 | 0 | 0.0341157 | 0.0995362 | 0.0059818 | 0.1837636 | 0 | 0.0125162 | 0 | 0.1392125 | 0.0137844 | 0.0085629 |
| TCGA-AU-6004-01A | 0.0695678 | 0 | 0.0050337 | 0.062016 | 0 | 0.1403358 | 0.0098199 | 0.0371551 | 0.033608 | 0 | 0.0508842 | 0 | 0 | 0.2155704 | 0.0578247 | 0.2827362 | 0.0108063 | 0 | 0 | 0.0209224 | 0 | 0.0037194 |
| TCGA-A6-5657-01A | 0.3886033 | 0 | 0 | 0.1110932 | 0 | 0.1404168 | 0 | 0.1129132 | 0.0460707 | 0 | 0 | 0 | 0 | 0.024878 | 0.0582786 | 0.0608016 | 0.0225135 | 0 | 0 | 0.028901 | 0.0055302 | 0 |
| TCGA-AA-3941-01A | 0.0312609 | 0.0951453 | 0 | 0.1310589 | 0 | 0.1704041 | 0 | 0.0614276 | 0.0142442 | 0 | 0.0217958 | 0 | 0.0534508 | 0 | 0 | 0.3200141 | 0.0129621 | 0.0350955 | 0 | 0.0453133 | 0.0078274 | 0 |
| TCGA-DM-A28K-01A | 0 | 0.0267812 | 0 | 0.0737227 | 0 | 0.0910626 | 0 | 0.0913978 | 0 | 0 | 0 | 0.0598545 | 0 | 0.2303154 | 0.0582591 | 0.2887481 | 0 | 0 | 0 | 0.0798587 | 0 | 0 |
| TCGA-CM-5344-01A | 0.0565073 | 0 | 0.0501893 | 0.0550493 | 0 | 0.0193272 | 0 | 0.026183 | 0.0487019 | 0 | 0 | 0.0426023 | 0 | 0.4496459 | 0.0380847 | 0.1945209 | 0 | 0 | 0.0191882 | 0 | 0 | 0 |
| TCGA-AA-3517-01A | 0.0123882 | 0.0073136 | 0.0604848 | 0.0785998 | 0.0046226 | 0.0126141 | 0.0598618 | 0.0395183 | 0.0739414 | 0 | 0.0962755 | 0 | 0.0571777 | 0.1835268 | 0 | 0.1243142 | 0.017072 | 0.0060289 | 0 | 0.1662602 | 0 | 0 |
| TCGA-G4-6309-01A | 0.0114208 | 0.0048082 | 0.0567262 | 0.1477181 | 0 | 0.1390268 | 0 | 0.1530852 | 0.042015 | 0 | 0.007376 | 0.0144133 | 0.0126562 | 0.0859627 | 0.0103816 | 0.1985231 | 0.0001878 | 0.0216112 | 0 | 0.094088 | 0 | 0 |
| TCGA-A6-3810-01A | 0.0364888 | 0 | 0.0333337 | 0.0886693 | 0 | 0.0940776 | 0 | 0.0993446 | 0.004884 | 0 | 0 | 0.0167506 | 0.0016342 | 0.1185828 | 0.0850025 | 0.2447508 | 0.0067572 | 0 | 0 | 0.1360531 | 0.0046935 | 0.0289772 |
| TCGA-AY-6386-01A | 0.0125643 | 0 | 0.040501 | 0.0373364 | 0 | 0.1502978 | 0 | 0.0686839 | 0.0484497 | 0 | 0.0440019 | 0 | 0 | 0.3213591 | 0.0381633 | 0.0949175 | 0 | 0 | 0 | 0.142358 | 0 | 0.0013672 |
| TCGA-AA-3678-01A | 0.0087192 | 0.025134 | 0.0609899 | 0.0530399 | 0 | 0.1815019 | 0.036578 | 0.0467377 | 0.0550277 | 0 | 0.1164469 | 0 | 0.0383806 | 0.0415368 | 0.0316202 | 0.1452282 | 0 | 0.0370747 | 0 | 0.1083305 | 0 | 0.0136539 |
| TCGA-AA-A01X-01A | 0.2886086 | 0 | 0.0246141 | 0.086627 | 0 | 0.18651 | 0 | 0.1365802 | 0.0081607 | 0 | 0 | 0.0252934 | 0.0148168 | 0.0309832 | 0.0109142 | 0.1490516 | 0.0238193 | 0.003686 | 0 | 0.010335 | 0 | 0 |
| TCGA-AA-3821-01A | 0.0078955 | 0.0016265 | 0.0147099 | 0.1125875 | 0 | 0.1690528 | 0.0055643 | 0.0347897 | 0 | 0 | 0.0467929 | 0.0005929 | 0.0252887 | 0.1090558 | 0.0613353 | 0.3378591 | 0.0067152 | 0 | 0.0118034 | 0.0495884 | 0 | 0.0047423 |
| TCGA-CM-6677-01A | 0.0245684 | 0.0370519 | 0.0170404 | 0.1206681 | 0 | 0.1472905 | 0.0518019 | 0.0476375 | 0.0484846 | 0 | 0.0855337 | 0.0042908 | 0.0189526 | 0.0629291 | 0.0450263 | 0.2244708 | 0.0172859 | 0.0093934 | 0 | 0.0375741 | 0 | 0 |
| TCGA-AA-3531-01A | 0.0007237 | 0.0168971 | 0.1159904 | 0.1896601 | 0 | 0 | 0.0191678 | 0.0357835 | 0.058806 | 0 | 0.0486047 | 0 | 0.027931 | 0.2002121 | 0.0403 | 0.1630997 | 0.016942 | 0 | 0 | 0.0621434 | 0.0037385 | 0 |
| TCGA-G4-6586-01A | 0.0345146 | 0 | 0.0089071 | 0.3759557 | 0 | 0 | 0.0290915 | 0.2383942 | 0.0254071 | 0 | 0 | 0.0795861 | 0.012552 | 0.0474686 | 0.0438587 | 0.0667223 | 0 | 0.00198 | 0 | 0.0355622 | 0 | 0 |
| TCGA-D5-6923-01A | 0.0360859 | 0 | 0.0209299 | 0.0071949 | 0.0015237 | 0.0817934 | 0.0002579 | 0.0198268 | 0 | 0 | 0.0118056 | 0.0102599 | 0 | 0.3340269 | 0.037154 | 0.2296204 | 0 | 0.0115876 | 0 | 0.1680331 | 0 | 0.0299002 |
| TCGA-F4-6703-01A | 0.0247447 | 0 | 0.0051776 | 0.0977878 | 0 | 0.1499896 | 0.0362148 | 0.0402612 | 0.0145028 | 0 | 0.0560508 | 0 | 0.0170149 | 0.1569934 | 0.0758007 | 0.2700185 | 0.002076 | 0 | 0 | 0.0461704 | 0 | 0.0071968 |
| TCGA-A6-3809-01B | 0 | 0.014865 | 0 | 0.0217359 | 0 | 0.2925186 | 0 | 0.0723067 | 0 | 0 | 0 | 0.0348787 | 0.0020123 | 0.0445921 | 0.0611543 | 0.1393005 | 0 | 0.0081688 | 0 | 0.2781445 | 0 | 0.0303226 |
| TCGA-A6-2677-01B | 0.0131723 | 0.0261067 | 0 | 0 | 0.0613499 | 0.4100379 | 0.0502944 | 0 | 0 | 0 | 0.0924159 | 0 | 0.0259556 | 0.1052239 | 0.0090075 | 0.1450084 | 0 | 0.0385501 | 0 | 0.0228773 | 0 | 0 |
| TCGA-NH-A6GB-01A | 0.0085557 | 0.0164831 | 0.0858983 | 0.0869183 | 0 | 0.085443 | 0.0601324 | 0.1236053 | 0.0146466 | 0 | 0.0374459 | 0.0214159 | 0.0229646 | 0.1544572 | 0.0322565 | 0.1710644 | 0.0091798 | 0 | 0 | 0.0695331 | 0 | 0 |
| TCGA-CM-5349-01A | 0.048225 | 0.0368717 | 0 | 0.0171153 | 0 | 0.0489695 | 0.0065906 | 0.0034931 | 0.0111585 | 0 | 0 | 0.0210887 | 0 | 0.6126298 | 0.0222077 | 0.1685232 | 0 | 0 | 0.003127 | 0 | 0 | 0 |
| TCGA-G4-6625-01A | 0.0248555 | 0.0371777 | 0.0137524 | 0.1154844 | 0 | 0.0969506 | 0.0010314 | 0.1083527 | 0.0610269 | 0 | 0 | 0.0301172 | 0.0003954 | 0.1238671 | 0.0926413 | 0.2315284 | 0 | 0.0104406 | 0.0523784 | 0 | 0 | 0 |
| TCGA-AA-3530-01A | 0.0215735 | 0.1121728 | 0.0325352 | 0.111017 | 0 | 0.1869313 | 0 | 0.0380256 | 0.0604033 | 0 | 0.0689296 | 0 | 0.0441795 | 0 | 0.0334324 | 0.1857279 | 0.021045 | 0 | 0 | 0.0840268 | 0 | 0 |
| TCGA-G4-6627-01A | 0.0788293 | 0.0342504 | 0.0544614 | 0.1426109 | 0 | 0.2239241 | 0 | 0.0606282 | 0.0235217 | 0 | 0 | 0 | 0 | 0.014484 | 0.0677896 | 0.2463558 | 0 | 0.0041734 | 0.0019499 | 0.0348488 | 0.0106012 | 0.0015714 |
| TCGA-DM-A28G-01A | 0.0025717 | 0.0122926 | 0.1207496 | 0.1784451 | 0.0008251 | 0.1311402 | 0.0525153 | 8.13E-06 | 0.020969 | 0 | 0.1267108 | 0 | 0.0490157 | 0.0938977 | 0.0230509 | 0.1247932 | 0.0144282 | 0 | 0 | 0.0485867 | 0 | 0 |
| TCGA-D5-6537-01A | 0 | 0.0200696 | 0.0142616 | 0.1831833 | 0 | 0.0944175 | 0.0064187 | 0.067505 | 0 | 0 | 0.0038804 | 0.0074234 | 0.0312677 | 0.1017558 | 0.0818085 | 0.1305406 | 0 | 0.0263964 | 0 | 0.1407891 | 0 | 0.0902824 |
| TCGA-D5-5539-01A | 0.0482934 | 0 | 0.0084122 | 0.0225999 | 0 | 0.1107737 | 0 | 0.042195 | 9.75E-05 | 0 | 0.0064683 | 0 | 0 | 0.3905008 | 0.0446222 | 0.1870207 | 0 | 0 | 0 | 0.1305534 | 0.0073781 | 0.0010849 |
| TCGA-CM-6163-01A | 0.0398317 | 0 | 0.047609 | 0.1493399 | 0 | 0.2428501 | 0 | 0.0490506 | 0.0382213 | 0 | 0 | 0 | 0 | 0.0841207 | 0.0471053 | 0.2196725 | 0.0381193 | 0.0013851 | 0 | 0.0060465 | 0.0128585 | 0.0237895 |
| TCGA-AZ-6607-01A | 0.0320276 | 0 | 0.0003167 | 0.1567136 | 0 | 0.1923336 | 0 | 0.0312774 | 0.0199204 | 0 | 0.0105345 | 0.0268375 | 0.0435227 | 0 | 0.0351142 | 0.2656339 | 0.0207634 | 0.0141627 | 0 | 0.1303977 | 0.0122291 | 0.0082151 |
| TCGA-AA-A004-01A | 0.0736256 | 0.0027629 | 0.019656 | 0.1423508 | 0 | 0.1384312 | 0 | 0.151686 | 0.1014582 | 0 | 0 | 0.1051964 | 0 | 0.1798076 | 0.0335902 | 0.0422922 | 0 | 0 | 0 | 0.0091429 | 0 | 0 |
| TCGA-AA-A00E-01A | 0.1024052 | 0 | 0.0004046 | 0.0843516 | 0 | 0.0633094 | 0 | 0.1206896 | 0.0135489 | 0 | 0 | 0.0418388 | 0 | 0.1884741 | 0.0999872 | 0.2117934 | 0.0109107 | 0 | 0 | 0.0622865 | 0 | 0 |
| TCGA-AA-A010-01A | 0.073101 | 0 | 0.0861055 | 0.1468443 | 0 | 0.0266411 | 0.0082481 | 0.0989237 | 0.057184 | 0 | 0 | 0.0377043 | 0.004018 | 0.0773992 | 0.0900891 | 0.221084 | 0 | 0 | 0 | 0.072658 | 0 | 0 |
| TCGA-AA-A02J-01A | 0.0248977 | 5.04E-05 | 0.1350834 | 0.0570136 | 0 | 0.1939193 | 0.0413574 | 0.0622292 | 0.0366957 | 0 | 0.0597238 | 0 | 0.0346836 | 0.1124669 | 0 | 0.0429352 | 0 | 0.0099502 | 0 | 0.1889937 | 0 | 0 |
| TCGA-AA-3869-01A | 0.0519666 | 0 | 0.0091634 | 0.1116449 | 0 | 0.0603416 | 0.0038687 | 0.0683947 | 0.0418582 | 0 | 0.0128207 | 0 | 0.0141606 | 0.1331747 | 0.070748 | 0.2890465 | 0 | 0 | 0 | 0.0782192 | 0 | 0.0545921 |
| TCGA-AA-3837-01A | 0.0898348 | 0 | 0.0228793 | 0.2020289 | 0 | 0.1148945 | 0.0066099 | 0.0281369 | 0.1298377 | 0 | 0 | 0.0030457 | 0.0576693 | 0 | 0 | 0.2547315 | 0.0049267 | 0.0035106 | 0 | 0.0818941 | 0 | 0 |
| TCGA-5M-AATA-01A | 0.0024185 | 0.0785277 | 0.0015186 | 0.1849444 | 0 | 0.1717332 | 0 | 0.0742226 | 0.0247091 | 0 | 0 | 0 | 0.0385943 | 0.0306161 | 0.0136712 | 0.1275269 | 0.0057944 | 0.0289839 | 0 | 0.2038398 | 0.0128993 | 0 |
| TCGA-CK-4947-01B | 0.0069637 | 0.0117728 | 0.0464204 | 0.1331427 | 0 | 0.2578384 | 0 | 0.0323758 | 0.0689943 | 0 | 0 | 0.0231168 | 0.0033203 | 0.0605448 | 0.0526728 | 0.1919557 | 0.0568257 | 0 | 0.0405123 | 0 | 0.0061527 | 0.0073908 |
| TCGA-CA-5256-01A | 0.0168144 | 0.0019132 | 0.015566 | 0.2479746 | 0 | 0.062436 | 0.0293477 | 0.0517953 | 0.0155922 | 0 | 0 | 0.0519399 | 0.0315474 | 0.0636738 | 0.0787667 | 0.1969092 | 0.1090261 | 0 | 0 | 0.0266973 | 0 | 0 |
| TCGA-CM-6167-01A | 0.0770167 | 0.047615 | 0 | 0.0697827 | 0 | 0.080751 | 0 | 0.058145 | 0.0461023 | 0 | 0 | 0.0272526 | 0.010502 | 0.0226925 | 0.0581892 | 0.2617365 | 0 | 0 | 0.2402147 | 0 | 0 | 0 |
| TCGA-4N-A93T-01A | 0.0375053 | 0.112621 | 0.2010551 | 0.102198 | 0 | 0.1097048 | 0 | 0.2235999 | 0.0214054 | 0 | 0 | 0.0565396 | 0.0305011 | 0.0155174 | 0.0078865 | 0.0628045 | 0 | 0.0186614 | 0 | 0 | 0 | 0 |
| TCGA-AA-3521-01A | 0.0297724 | 0.0154403 | 0.0368172 | 0.1268867 | 0 | 0.1887983 | 0 | 0.0922856 | 0.0289073 | 0 | 0.0057318 | 0 | 0.0459761 | 0.0522893 | 0.0053737 | 0.2598364 | 0.0229892 | 0.0061686 | 0 | 0.0561259 | 0 | 0.0266014 |
| TCGA-CA-5797-01A | 0.0494819 | 0 | 0.0851438 | 0.1114249 | 0 | 0.0417556 | 0 | 0.0593551 | 0.060212 | 0 | 0 | 0.0464649 | 0 | 0.183192 | 0.1382009 | 0.1286302 | 0.0150002 | 0 | 0.0811387 | 0 | 0 | 0 |
| TCGA-CM-6166-01A | 0.0172822 | 0.0264317 | 0 | 0.0582901 | 0 | 0.0063747 | 0.0631391 | 0 | 0.0526365 | 0 | 0.0913708 | 0 | 0 | 0.4967124 | 0.0114687 | 0.0956132 | 0 | 0 | 0 | 0.0806805 | 0 | 0 |
| TCGA-CK-4951-01A | 0.0083982 | 0.0019852 | 0 | 0.1109634 | 0 | 0.1765807 | 0 | 0.0475364 | 0.0506924 | 0 | 0.0406948 | 0.0488367 | 0.0409122 | 0.1086112 | 0.0738236 | 0.1796312 | 0.0033494 | 0.0130108 | 0 | 0.0949738 | 0 | 0 |
| TCGA-CA-6719-01A | 0.0212739 | 0.0153009 | 0.0088923 | 0.0929025 | 0 | 0 | 0 | 0.0213384 | 0.0938153 | 0 | 0.0297582 | 0 | 0 | 0.326237 | 0.0575347 | 0.2147871 | 0.0004837 | 0 | 0 | 0.117676 | 0 | 0 |
| TCGA-AA-A02R-01A | 0.0220075 | 0 | 0.000961 | 0.2208717 | 0 | 0 | 0.1049073 | 0.0990861 | 0.0210619 | 0 | 0.0059931 | 0.0458706 | 0.0345292 | 0.0167503 | 0.1983356 | 0.179097 | 0.0289034 | 0 | 0.0216252 | 0 | 0 | 0 |
| TCGA-AA-A02O-01A | 0 | 0.0154793 | 0.1093286 | 0.1680662 | 0 | 0.0758821 | 0.0082777 | 0.149123 | 0.1145063 | 0 | 0.0139839 | 0.0043424 | 0.0360473 | 0.0666556 | 0.0036448 | 0.1538532 | 0.0083945 | 0 | 0 | 0.0724152 | 0 | 0 |
| TCGA-AY-5543-01A | 0.0452262 | 0.0156717 | 0.0381474 | 0.1159735 | 0 | 0.1375638 | 0.0287731 | 0.1370611 | 0.0155153 | 0 | 0.0045121 | 0.0166258 | 0 | 0.0746471 | 0.0859049 | 0.166755 | 0 | 0.0209973 | 0 | 0.0966258 | 0 | 0 |
| TCGA-AA-3845-01A | 0.0455468 | 0 | 0.0367922 | 0.1831589 | 0 | 0.0617453 | 0.0627521 | 0.0904724 | 0.0264063 | 0 | 0.011045 | 0.0260252 | 0 | 0.0390872 | 0.0713096 | 0.201742 | 0 | 0.0200307 | 0 | 0.0773953 | 0.0238004 | 0.0226907 |
| TCGA-CK-5912-01A | 0.1076222 | 0 | 0.0482884 | 0.0247807 | 0 | 0.234827 | 0.0211927 | 0.005281 | 9.88E-05 | 0 | 0.0381565 | 0 | 0.0021216 | 0.2950174 | 0.0260277 | 0.130416 | 0.0052537 | 0 | 0 | 0.0609165 | 0 | 0 |
| TCGA-AA-3856-01A | 0.0090288 | 0.0256984 | 0.0410133 | 0.109923 | 0 | 0.0876514 | 0 | 0.0738872 | 0.1185169 | 0 | 0 | 0.044199 | 0.0119686 | 0.079305 | 0.07008 | 0.2442127 | 0.0439772 | 0 | 0.0405385 | 0 | 0 | 0 |
| TCGA-AA-A00Q-01A | 0.0407673 | 0 | 0.0581108 | 0.1500935 | 0 | 0.0434896 | 0.0172593 | 0.0794397 | 0.0923314 | 0 | 0 | 0.0242028 | 0.0287851 | 0.0460419 | 0.1265925 | 0.2418848 | 0.0304573 | 0 | 0.020544 | 0 | 0 | 0 |
| TCGA-A6-6781-01B | 0.0327882 | 0 | 0 | 0.0051124 | 0 | 0.193384 | 0 | 0.0447992 | 0 | 0 | 0.0159582 | 0 | 0 | 0.0188003 | 0.0554002 | 0.1605707 | 0 | 0.0217716 | 0 | 0.3189682 | 0.0160474 | 0.1163997 |
| TCGA-AA-A00F-01A | 0 | 0.0632405 | 0.1041432 | 0.0578457 | 0 | 0.0889312 | 0 | 0.1592654 | 0.0385637 | 0 | 0 | 0.0379581 | 0.0046407 | 0.0747388 | 0.0106454 | 0.2663968 | 0 | 0.0289708 | 0.0215994 | 0.0430602 | 0 | 0 |
| TCGA-D5-6920-01A | 0 | 0.0215836 | 0.04603 | 0.0816975 | 0 | 0.2243929 | 0.0194129 | 0.0439618 | 0.019208 | 0 | 0.0702958 | 0.0133746 | 0.0226142 | 0.131555 | 0.0082652 | 0.1210402 | 0 | 0.0386965 | 0 | 0.1378718 | 0 | 0 |
| TCGA-AA-3815-01A | 0.0441681 | 0 | 0.0037663 | 0.2952604 | 0 | 0 | 0.0279079 | 0.0882064 | 0.0297198 | 0 | 0.0539918 | 0.0315562 | 0.0336102 | 0.0437677 | 0.0944375 | 0.1770397 | 0.004417 | 0 | 0 | 0.0721509 | 0 | 0 |
| TCGA-D5-5538-01A | 0.0529056 | 0 | 0.012989 | 0.0577312 | 0 | 0.094032 | 0 | 0.0370291 | 0.0510187 | 0 | 0.0202943 | 0 | 0.0099235 | 0.1786007 | 0.0559539 | 0.3238518 | 0.0162665 | 0 | 0 | 0.0746064 | 0.0064276 | 0.0083698 |
| TCGA-A6-A566-01A | 0.0063194 | 0.0060698 | 0.0046626 | 0.0389479 | 0 | 0.0421525 | 0 | 0.0163178 | 0.0400547 | 0 | 0.0715352 | 0 | 0 | 0.3422047 | 0 | 0.3162263 | 0.0024668 | 0 | 0 | 0.1130422 | 0 | 0 |
| TCGA-A6-6782-01A | 0.0426359 | 0 | 0.0469302 | 0.1828665 | 0 | 0.0066555 | 0.0624189 | 0.0301389 | 0.0162996 | 0 | 0.0656287 | 0 | 0.139138 | 0.0880126 | 0.0613501 | 0.1478425 | 0.0086966 | 0 | 0 | 0.1013859 | 0 | 0 |
| TCGA-CM-6168-01A | 0.0661614 | 0.000754 | 0.0026407 | 0.035684 | 0 | 0.1559974 | 0 | 0.066947 | 0.0210389 | 0 | 0.0099128 | 0 | 0 | 0.1396017 | 0.0597646 | 0.3072641 | 0 | 0 | 0.0796553 | 0.0279489 | 0.009612 | 0.0170174 |
| TCGA-A6-6654-01A | 0.0228085 | 0 | 0.0024329 | 0.0679228 | 0 | 0.0821514 | 0 | 0.0522428 | 0.0378724 | 0 | 0.0157781 | 0 | 0 | 0.3162441 | 0.0542367 | 0.2756986 | 0 | 0 | 0 | 0.0726117 | 0 | 0 |
| TCGA-AA-A01D-01A | 0.0018382 | 0.0198062 | 0 | 0 | 0 | 0.0460783 | 0 | 0.038053 | 0.0449933 | 0 | 0.0293893 | 0.0088883 | 0 | 0.5695556 | 0.0109206 | 0.1503103 | 0 | 0 | 0 | 0.0801669 | 0 | 0 |
| TCGA-QG-A5YV-01A | 0 | 0.0184134 | 0.0887865 | 0.1865136 | 0 | 0.0992464 | 0 | 0.0799481 | 0.0572077 | 0 | 0.0272874 | 0 | 0.0294466 | 0.0629622 | 0.0878963 | 0.2306703 | 0.0105259 | 0.0023986 | 0 | 0.018697 | 0 | 0 |
| TCGA-A6-3807-01A | 0.0307218 | 0.0147013 | 0 | 0.1519037 | 0 | 0.1407297 | 0 | 0.1068966 | 0.02816 | 0 | 0.0329031 | 0.0047626 | 0.031881 | 0 | 0.0794462 | 0.2074045 | 0.0286261 | 0.003738 | 0 | 0.0630631 | 0.0223036 | 0.0527585 |
| TCGA-AZ-5403-01A | 0.0029834 | 0.0339697 | 0.0080959 | 0.0467244 | 0 | 0.0970217 | 0.0049659 | 0.0153606 | 0.0380973 | 0 | 0.0500727 | 0.0260724 | 0 | 0.2723386 | 0.0170162 | 0.2644641 | 0.018318 | 0 | 0 | 0.0831505 | 0 | 0.0213487 |
| TCGA-AD-6888-01A | 0.0709509 | 0 | 0.1211791 | 0.0882318 | 0 | 0.174311 | 0 | 0.0867102 | 0.0242766 | 0 | 0 | 0.0916042 | 0.0088814 | 0.1085918 | 0.0426056 | 0.1200977 | 0 | 0.0446686 | 0.0178911 | 0 | 0 | 0 |
| TCGA-G4-6628-01A | 0.0410016 | 0 | 0.0136634 | 0.2208236 | 0 | 0.0984765 | 0.0311332 | 0.0910892 | 0.0173933 | 0 | 0 | 0.0684535 | 0.032748 | 0 | 0.0776985 | 0.2836068 | 0.0042782 | 0.0166312 | 0 | 0.003003 | 0 | 0 |
| TCGA-DM-A1D6-01A | 0 | 0.0557371 | 0.0029651 | 0.1575851 | 0 | 0.192053 | 0 | 0.0832595 | 0 | 0 | 0 | 0.0385416 | 0.0139 | 0.1232488 | 0 | 0.2530772 | 0 | 0.0478923 | 0 | 0.0317403 | 0 | 0 |
| TCGA-AA-A00K-01A | 0.0165259 | 0.0338674 | 0.0509843 | 0.054719 | 0 | 0.0668489 | 0.0152274 | 0.0589407 | 0.1165478 | 0 | 0.0303859 | 0 | 0 | 0.1598245 | 0.0145487 | 0.2569381 | 0 | 0 | 0 | 0.1246414 | 0 | 0 |
| TCGA-AA-3862-01A | 0.0064738 | 0.0108711 | 0.0205956 | 0.2113283 | 0 | 0.0276327 | 0.0717716 | 0.1119058 | 0.0480619 | 0 | 0 | 0.024489 | 0 | 0.0950482 | 0.0850165 | 0.1872263 | 0 | 0 | 0.0337392 | 0.017575 | 0.0349531 | 0.013312 |
| TCGA-AA-3939-01A | 0.0467117 | 0 | 0.0126961 | 0.0894743 | 0 | 0.1157247 | 0 | 0.1473759 | 0.0221119 | 0 | 0 | 0.0083856 | 0.0028651 | 0.0550977 | 0.0413703 | 0.2720295 | 0.012581 | 0 | 0 | 0.16908 | 0 | 0.0044964 |
| TCGA-AA-3514-01A | 0.0170781 | 0.0080223 | 0.0045038 | 0.0618636 | 0 | 0.0809672 | 0 | 0.0228544 | 0.0994857 | 0 | 0.0610275 | 0.0015823 | 0.0039948 | 0.3662376 | 0.021556 | 0.1646303 | 0 | 0 | 0 | 0.0861964 | 0 | 0 |
| TCGA-AA-3660-01A | 0.0175597 | 0.0148059 | 0.0747254 | 0.0716932 | 0 | 0.0801833 | 0.1081633 | 0 | 0.0195659 | 0 | 0.0834327 | 0 | 0.0414534 | 0 | 0 | 0.338899 | 0.0431625 | 0.036378 | 0.0155569 | 0.0521502 | 0 | 0.0022704 |
| TCGA-AA-3833-01A | 0.0382019 | 0 | 0.0056897 | 0.1587779 | 0 | 0.1071345 | 0 | 0.0999116 | 0.053835 | 0 | 0.0050666 | 0.0038399 | 0.0072443 | 0.0767627 | 0.0287648 | 0.1965254 | 0 | 0 | 0 | 0.1752067 | 0.0133271 | 0.0297119 |
| TCGA-AA-A01P-01A | 0.0396403 | 0 | 0.0741657 | 0.2204482 | 0 | 0 | 0.0226509 | 0.0939197 | 0.0907791 | 0 | 0.0234371 | 0.0440817 | 0.003103 | 0.0654903 | 0.1087097 | 0.1886702 | 0 | 0 | 0 | 0.0194191 | 0 | 0.0054849 |
| TCGA-CM-5863-01A | 0.0336326 | 0.0284063 | 0.019784 | 0.1578393 | 0 | 0.1687217 | 0 | 0.0114578 | 0.0205597 | 0 | 0.0004778 | 0 | 0.1201509 | 0.1116445 | 0.0161136 | 0.1849812 | 0.0055359 | 0 | 0 | 0.1206948 | 0 | 0 |
| TCGA-AZ-6598-01A | 0.0231357 | 0 | 0.0060579 | 0.0471838 | 0 | 0.2740989 | 0.0300663 | 0 | 0 | 0 | 0.0602037 | 0.1145542 | 0.0433118 | 0.0638061 | 0.0550795 | 0.2371622 | 0.0177932 | 0 | 0.0275466 | 0 | 0 | 0 |
| TCGA-AA-3679-01A | 0 | 0.0168986 | 0.0941515 | 0.0560637 | 0 | 0.1489498 | 0 | 0.03726 | 0.0689413 | 0 | 0.0949936 | 0 | 0.0069092 | 0.1472872 | 0.056144 | 0.226833 | 0 | 0 | 0 | 0.0309585 | 0.0073015 | 0.0073082 |
| TCGA-G4-6304-01A | 0.0059496 | 0.0146295 | 0 | 0.182938 | 0.1036717 | 0 | 0.0658067 | 0.0050876 | 0 | 0 | 0.0412241 | 0 | 0.0168897 | 0.0305912 | 0.1472293 | 0.3000306 | 0 | 0 | 0 | 0.0859521 | 0 | 0 |
| TCGA-DM-A285-01A | 0.0036464 | 0.0552458 | 0 | 0.0485423 | 0 | 0.164641 | 0 | 0.0275401 | 0.1040789 | 0 | 0.0022653 | 0.0419978 | 0.0304735 | 0.3449067 | 0.0069588 | 0.0625999 | 0.0068153 | 0 | 0 | 0.0637394 | 0.0365489 | 0 |
| TCGA-G4-6294-01A | 0.0348023 | 0 | 0.0328121 | 0.0586164 | 0 | 0.3295717 | 0.044925 | 0.0716183 | 0 | 0 | 0.0445056 | 0 | 0.0037863 | 0.0659051 | 0.0229428 | 0.1896387 | 0.0661702 | 0 | 0 | 0.0347054 | 0 | 0 |
| TCGA-A6-4107-01A | 0.0229604 | 0.0145796 | 0.0551409 | 0.063709 | 0 | 0.1485792 | 0 | 0.06575 | 0.1164286 | 0 | 0.0506064 | 0 | 0.0248743 | 0.0332273 | 0.0628134 | 0.1922404 | 0.0433105 | 0 | 0 | 0.10578 | 0 | 0 |
| TCGA-CA-6716-01A | 0.015099 | 0 | 0.0996958 | 0.0039802 | 0 | 0.1733207 | 0.0108044 | 0.0082488 | 0.0117664 | 0 | 0.0571156 | 0 | 0 | 0.1736128 | 0.0138348 | 0.361196 | 0 | 0 | 0 | 0.0713256 | 0 | 0 |
| TCGA-CK-4952-01A | 0 | 0 | 0.0206134 | 0.0057083 | 0 | 0.2009367 | 0 | 0 | 0 | 0 | 0.0182856 | 0.0078698 | 0.0109978 | 0.2087986 | 0.0096043 | 0.2284213 | 0 | 0.0311238 | 0 | 0.1282195 | 0.0236442 | 0.1057766 |
| TCGA-G4-6302-01A | 0.0220209 | 0 | 0.0150096 | 0.1371065 | 0 | 0.1100231 | 0 | 0.0096964 | 0.070236 | 0 | 0 | 0 | 0 | 0.0816799 | 0.060991 | 0.4652451 | 0 | 0 | 0.0276028 | 0 | 0 | 0.0003886 |
| TCGA-AA-3844-01A | 0.0328859 | 0 | 0.0489083 | 0.0485621 | 0 | 0.2937635 | 0.0650377 | 0.0836111 | 0.0577137 | 0 | 0.0325637 | 0 | 0.0137051 | 0.0037523 | 0.0201543 | 0.1424238 | 0.0058079 | 0.0231641 | 0 | 0.1279468 | 0 | 0 |
| TCGA-D5-6541-01A | 0.0540346 | 0 | 0.0225504 | 0.1134288 | 0 | 0.0602751 | 0.032441 | 0.0153037 | 0.0226848 | 0 | 0.0317287 | 0 | 0.008783 | 0.0992093 | 0.1497372 | 0.3215389 | 0.0107715 | 0 | 0 | 0.044231 | 0 | 0.0132821 |
| TCGA-AA-A01G-01A | 0 | 0.0921451 | 0.0421921 | 0.2513472 | 0 | 0.0139365 | 0 | 0.1118421 | 0.0370444 | 0 | 0 | 0.0729684 | 0.0241929 | 0.0943175 | 0.0113222 | 0.0678531 | 0.0023019 | 0.0241893 | 0 | 0.1319202 | 0.0224271 | 0 |
| TCGA-AA-3870-01A | 0.0316938 | 0 | 0.0214794 | 0.2118112 | 0 | 0.0278459 | 0.012977 | 0.0507727 | 0.0364849 | 0 | 0.0171514 | 0 | 0 | 0.0519836 | 0.0502304 | 0.4000388 | 0.0259506 | 0 | 0.0084634 | 0.0424291 | 0 | 0.0106877 |
| TCGA-D5-6532-01A | 0.0263051 | 0.0259804 | 0.1009126 | 0.1384794 | 0 | 0.1162014 | 0.0653411 | 0 | 0.0215544 | 0 | 0.0195119 | 0.0525403 | 0.0658551 | 0.1666451 | 0.0774293 | 0.0770289 | 0.0134147 | 0 | 0.0328001 | 0 | 0 | 0 |
| TCGA-G4-6322-01A | 0.0378216 | 0 | 0.0651229 | 0.044487 | 0 | 0.2605604 | 0 | 0.0717898 | 0.0564879 | 0 | 0.0144909 | 0.0013692 | 0 | 0.1883224 | 0.0412189 | 0.1638635 | 0 | 0 | 0 | 0.0544655 | 0 | 0 |
| TCGA-D5-5541-01A | 0.0523679 | 0 | 0.0209169 | 0.0648203 | 0 | 0 | 0.0116186 | 0.0591999 | 0.0587447 | 0.0035427 | 0.013857 | 0 | 0 | 0.4291031 | 0.0573692 | 0.1414721 | 0.007877 | 0 | 0 | 0.0791106 | 0 | 0 |
| TCGA-DM-A1DB-01A | 0 | 0.0338136 | 0.0387253 | 0.2399653 | 0 | 0.1631958 | 0 | 0.1445705 | 0.0358322 | 0 | 0 | 0.0969671 | 0 | 0.0729279 | 0.0534675 | 0.1048995 | 0 | 0.0028137 | 0.0128215 | 0 | 0 | 0 |
| TCGA-AA-A00A-01A | 0.0592402 | 0 | 0.0518797 | 0.120326 | 0 | 0.0014188 | 0 | 0.1211444 | 0.0566332 | 0 | 0 | 0.0802497 | 0.0054118 | 0.0782553 | 0.1003593 | 0.2020747 | 0 | 0 | 0 | 0.1213096 | 0 | 0.0016973 |
| TCGA-A6-2684-01A | 0 | 0.0241687 | 0.0121341 | 0.064687 | 0 | 0.1509458 | 0 | 0.0382 | 0.0494185 | 0 | 0.0059348 | 0.0049887 | 0.0342112 | 0.1116861 | 0.0880201 | 0.3359988 | 0.0524261 | 0 | 0.0182518 | 0.0042692 | 0.0046592 | 0 |
| TCGA-AA-3855-01A | 0.0032131 | 0.0197977 | 0.0904877 | 0.1673132 | 0 | 0.1097912 | 0.0226519 | 0.0224694 | 0.0934356 | 0 | 0.0652737 | 0 | 0.0556734 | 0.0314565 | 0.0224437 | 0.2005428 | 0.0164003 | 0 | 0 | 0.0790498 | 0 | 0 |
| TCGA-F4-6463-01A | 0.1150584 | 0 | 0.0235393 | 0.2358962 | 0.0364079 | 0 | 0.0109432 | 0.0417409 | 0.0092499 | 0 | 0 | 0.0757179 | 0.0481569 | 0.0377616 | 0.0547013 | 0.2480755 | 0.0197748 | 0.0134482 | 0.0183444 | 0.0111837 | 0 | 0 |
| TCGA-NH-A6GC-01A | 0.0130191 | 0.1012721 | 0.168284 | 0.0829628 | 0 | 0.204324 | 0 | 0 | 0.0693085 | 0 | 0.0051611 | 0.0227064 | 0.0449983 | 0.1490107 | 0 | 0.0971355 | 0 | 0 | 0.0418176 | 0 | 0 | 0 |
| TCGA-AA-3526-01A | 0.0262049 | 0 | 0.018733 | 0.1400262 | 0 | 0.0889615 | 0.0001586 | 0.1627848 | 0.0100015 | 0 | 0 | 0.0143585 | 0 | 0.0940078 | 0.1663022 | 0.1673242 | 0.0108955 | 0 | 0 | 0.0560971 | 0.0302854 | 0.0138588 |
| TCGA-DM-A28M-01A | 0.0219477 | 0 | 0 | 0.1636067 | 0 | 0.2601032 | 0.0006618 | 0.0801302 | 0.0624789 | 0 | 0 | 0.0408467 | 0.028305 | 0 | 0.0026466 | 0.1220414 | 0.0209737 | 0.0155092 | 0 | 0.1807488 | 0 | 0 |
| TCGA-D5-6926-01A | 0.0247436 | 0 | 0.0457702 | 0.0619095 | 0 | 0.0544853 | 0.0211188 | 0.0319314 | 0.006805 | 0 | 0.0597295 | 0 | 0.0123844 | 0.2508036 | 0.1700488 | 0.169129 | 0 | 0 | 0 | 0.0598875 | 0 | 0.0312534 |
| TCGA-G4-6317-01A | 0.005756 | 0.0017836 | 0 | 0.6135937 | 0 | 0 | 0.1191148 | 0.0007533 | 0 | 0 | 0.0439079 | 0 | 0.0524928 | 0.0335512 | 0.0046962 | 0 | 0.0114622 | 0 | 0 | 0.1041743 | 0.0087141 | 0 |
| TCGA-AA-A01K-01A | 0 | 0.0389068 | 0.0383442 | 0.0748677 | 0 | 0 | 0 | 0.1412247 | 0.1078674 | 0 | 0.0102409 | 0 | 0 | 0.165384 | 0.0214655 | 0.2548992 | 0.0015859 | 0 | 0 | 0.1452137 | 0 | 0 |
| TCGA-AA-3984-01A | 0.0042296 | 0.0147242 | 0.027862 | 0.1403054 | 0 | 0.0918371 | 0.0094718 | 0.1551428 | 0.058415 | 0 | 0.0481041 | 0 | 0 | 0.0941784 | 0.0974754 | 0.1402816 | 0.0017163 | 0.0141856 | 0 | 0.1018635 | 0 | 0.000207 |
| TCGA-D5-6536-01A | 0 | 0 | 0 | 0 | 0 | 0.0836919 | 0 | 0 | 0.0021908 | 0 | 0.0043649 | 0.0043825 | 0 | 0.3453719 | 0.0003 | 0.237351 | 0 | 0.0067163 | 0 | 0.2333883 | 0 | 0.0822424 |
| TCGA-AD-A5EK-01A | 0.0163583 | 0 | 0.018838 | 0.0798951 | 0 | 0.1921945 | 0 | 0.1775702 | 0 | 0 | 0.0583581 | 0 | 0.0334521 | 0.1413132 | 0.0112554 | 0.1961242 | 0 | 0.0210477 | 0 | 0.0535932 | 0 | 0 |
| TCGA-AA-3663-01A | 0.0329157 | 0 | 0.0193038 | 0.047392 | 0 | 0.1659023 | 0 | 0.0624277 | 0.0096312 | 0 | 0 | 0.0365427 | 0.0122184 | 0.1030069 | 0.07126 | 0.2950608 | 0.0084403 | 0 | 0 | 0.1281922 | 0.0014478 | 0.0062583 |
| TCGA-AD-6965-01A | 0.0610465 | 0.0678886 | 0 | 0.2018256 | 0 | 0.1087206 | 0.05258 | 0 | 0.0144887 | 0 | 0.0489868 | 0.0193801 | 0.0309287 | 0.2129015 | 0.0154309 | 0.1349045 | 0 | 0 | 0.0176177 | 0.0132998 | 0 | 0 |
| TCGA-CM-4744-01A | 0.0217192 | 0.006229 | 0 | 0.407641 | 0 | 0 | 0.0449416 | 0.1114026 | 0.062966 | 0 | 0 | 0.0629753 | 0.0314597 | 0 | 0.0383071 | 0.0898116 | 0.0222718 | 0 | 0 | 0.0921546 | 0.0081206 | 0 |
| TCGA-AA-3712-01A | 0.0199202 | 0.019101 | 0 | 0.1286436 | 0 | 0.1265192 | 0.1129316 | 0.0243105 | 0 | 0 | 0.0237007 | 0.0333092 | 0.0475255 | 0.0969528 | 0.0109149 | 0.2124956 | 0.0006474 | 0.0088145 | 0 | 0.1118548 | 0.0223587 | 0 |
| TCGA-AA-3543-01A | 0.000307 | 0.0025106 | 0.012123 | 0.3041028 | 0 | 0 | 0.0373506 | 0.1154038 | 0.0658556 | 0 | 0 | 0.0524008 | 0 | 0.0163961 | 0.1009782 | 0.2384891 | 0.0010904 | 0 | 0 | 0.0314449 | 0 | 0.0215472 |
| TCGA-AZ-5407-01A | 0.0131557 | 0 | 0.1127444 | 0.1257162 | 0 | 0.3551089 | 0 | 0.0983345 | 0.0901749 | 0 | 0 | 0 | 0.0050798 | 0.0164011 | 0.0142474 | 0.1166631 | 0.0179224 | 0.0103226 | 0.0111828 | 0.0129462 | 0 | 0 |
| TCGA-G4-6314-01A | 0 | 0.0225554 | 0.0307185 | 0.0450485 | 0 | 0.2149072 | 0 | 0.0664212 | 0.0358428 | 0 | 0.0187187 | 0.0033189 | 0.0159899 | 0.0898606 | 0.065007 | 0.3414186 | 0.0178562 | 0 | 0.0323365 | 0 | 0 | 0 |
| TCGA-CM-5348-01A | 0.0029804 | 0.0083862 | 0.0034943 | 0.0135027 | 0 | 0.0586385 | 0 | 0.0103157 | 0.022539 | 0 | 0.0051313 | 0.0096585 | 0.0021643 | 0.5742917 | 0.0183414 | 0.2172322 | 0.0031495 | 0.0076828 | 0.0424916 | 0 | 0 | 0 |
| TCGA-CK-6751-01A | 0.0089117 | 0.0195882 | 0 | 0.1905302 | 0 | 0.0912682 | 0 | 0.0944634 | 0.0507253 | 0 | 0.0027765 | 0 | 0.0385659 | 0.1393885 | 0.0199294 | 0.1137961 | 0.0033517 | 0.024075 | 0 | 0.1856386 | 0.0169913 | 0 |
| TCGA-D5-6929-01A | 0 | 0.0155638 | 0.2436144 | 0.1235443 | 0 | 0.0334647 | 0.0069823 | 0.0545677 | 0.0197669 | 0 | 0.0227603 | 0 | 0 | 0.2312278 | 0.0423703 | 0.164854 | 0 | 0 | 0 | 0.0412837 | 0 | 0 |
| TCGA-AA-3675-01A | 0.0702259 | 0 | 0.0447133 | 0.0872907 | 0 | 0.1425855 | 0 | 0.0649189 | 0.0260832 | 0 | 0.0099231 | 0.0065453 | 0.0112744 | 0.1167099 | 0.06983 | 0.1923866 | 0 | 0.0131456 | 0 | 0.1186722 | 0.0068092 | 0.0188863 |
| TCGA-A6-2681-01A | 0.0427757 | 0 | 0 | 0.1086887 | 0 | 0.0228269 | 0 | 0.0509575 | 0.0458653 | 0 | 0 | 0 | 0.0016153 | 0.349568 | 0.0290514 | 0.2112322 | 0 | 0 | 0 | 0.1374191 | 0 | 0 |
| TCGA-F4-6808-01A | 0.0497887 | 0 | 0.0935013 | 0.0845539 | 0 | 0.1754818 | 0.0785311 | 0.0563322 | 0 | 0 | 0.0553059 | 0 | 0.0301545 | 0.1171433 | 0.0157496 | 0.0391889 | 0 | 0.0087524 | 0 | 0.1946584 | 0.000858 | 0 |
| TCGA-DM-A282-01A | 0.0083589 | 0.0628282 | 0 | 0.1845718 | 0 | 0.1200126 | 0 | 0.0711753 | 0.0394337 | 0 | 0.0070629 | 0.0462648 | 0.0378408 | 0.1181013 | 0.0362533 | 0.2583412 | 0.0097552 | 0 | 0 | 0 | 0 | 0 |
| TCGA-AA-3489-01A | 0.114219 | 0.0696153 | 0.0257159 | 0.0820448 | 0 | 0.154548 | 0 | 0.0590301 | 0.0231521 | 0 | 0.0537567 | 0 | 0.0594924 | 0.046151 | 0.0400247 | 0.2152718 | 0 | 0 | 0.0060271 | 0.0477065 | 0 | 0.0032446 |
| TCGA-AM-5821-01A | 0.0660809 | 0 | 0.0457006 | 0.2230807 | 0 | 0 | 0.0244129 | 0.0515386 | 0 | 0 | 0 | 0.075121 | 0.0103846 | 0.0218409 | 0.2185392 | 0.1335849 | 0 | 0 | 0 | 0.1024252 | 0 | 0.0272904 |
| TCGA-G4-6298-01A | 0.0354905 | 0.0572548 | 0 | 0.0661373 | 0.0177184 | 0.1040932 | 0.0616088 | 0 | 0.0179237 | 0 | 0.0618302 | 0 | 0.0341512 | 0.1966492 | 0 | 0.1401884 | 0.0006514 | 0.0009482 | 0 | 0.2053546 | 0 | 0 |
| TCGA-AA-A01S-01A | 0 | 0.17667 | 0.1013048 | 0.1298354 | 0.0393929 | 0.0217482 | 0 | 0.0348128 | 0 | 0 | 0 | 0.0291301 | 0.0321562 | 0.0894851 | 0 | 0.1881461 | 0.0073987 | 0 | 0 | 0.1499198 | 0 | 0 |
| TCGA-DM-A28H-01A | 0.1005325 | 0 | 0.1595088 | 0.0504904 | 0 | 0.0655627 | 0 | 0.0713178 | 0 | 0 | 0 | 0.0209617 | 0 | 0.1039562 | 0.0493284 | 0.3081978 | 0 | 0 | 0 | 0.0701437 | 0 | 0 |
| TCGA-AA-3966-01A | 0.0275529 | 0 | 0.0094952 | 0.0795578 | 0 | 0.0404649 | 0.0336209 | 0.0502052 | 0.0159497 | 0 | 0.0132668 | 0 | 0.0087445 | 0.289624 | 0.0135049 | 0.1714035 | 0 | 0.0109723 | 0 | 0.200027 | 0.0106086 | 0.0250019 |
| TCGA-AA-3516-01A | 0 | 0.0272876 | 0.0106486 | 0.2068898 | 0 | 0 | 0.0447691 | 0.1056476 | 0 | 0 | 0 | 0.044872 | 0.0333118 | 0 | 0.0716116 | 0.2063962 | 0 | 0.0766645 | 0 | 0.1619566 | 0 | 0.0099444 |
| TCGA-4T-AA8H-01A | 0.0069116 | 0 | 0.1883569 | 0.1445706 | 0 | 0.3000478 | 0 | 0 | 0 | 0 | 0.0553326 | 0 | 0.022142 | 0.0002393 | 0 | 0.1522308 | 0.0512056 | 0.0167789 | 0 | 0.0621841 | 0 | 0 |
| TCGA-AA-3544-01A | 0.0195112 | 0.0097624 | 0.0131557 | 0.0858493 | 0 | 0 | 0.0352721 | 0.0858083 | 0.0367779 | 0.0045034 | 0 | 0.030637 | 0 | 0.2063327 | 0.0766698 | 0.31332 | 0.0167742 | 0 | 0 | 0.0656261 | 0 | 0 |
| TCGA-AA-3956-01A | 0.1573014 | 0 | 0.1714246 | 0.0883123 | 0 | 0.1139642 | 0 | 0.0464834 | 0.1040966 | 0 | 0 | 0.0131073 | 0 | 0.0279895 | 0.0181231 | 0.0991771 | 0.0525897 | 0.0077837 | 0.0810116 | 0.0186355 | 0 | 0 |
| TCGA-AA-3532-01A | 0.084849 | 0 | 0.0137104 | 0.059183 | 0 | 0.1347349 | 0 | 0.0979536 | 0.0577556 | 0 | 0.001018 | 0.0149959 | 0.0003701 | 0.1095636 | 0.1110438 | 0.2539796 | 0.006748 | 0.0111447 | 0.0129709 | 0.010824 | 0 | 0.0191549 |
| TCGA-AA-3955-01A | 0.0328184 | 0 | 0.0172969 | 0.034155 | 0 | 0.107432 | 0 | 0.082873 | 0.0060406 | 0 | 0 | 0.050199 | 0.003194 | 0.1340826 | 0.0371641 | 0.264938 | 0.0401831 | 0 | 0.0497576 | 0.1353771 | 0 | 0.0044887 |
| TCGA-F4-6704-01A | 0.0237674 | 0 | 0.0524574 | 0.0636251 | 0 | 0.2054649 | 0 | 0.0250428 | 0.0272477 | 0 | 0.0032994 | 0.0108416 | 2.18E-05 | 0.1230715 | 0.039864 | 0.3718342 | 0 | 0 | 0.0495249 | 0 | 0.0024945 | 0.0014427 |
| TCGA-AD-6895-01A | 0.0005103 | 0 | 0 | 0.250745 | 0 | 0 | 0.0439861 | 0.2315217 | 0 | 0 | 0.0207089 | 0.0675619 | 0 | 0.0815126 | 0.0905969 | 0.168436 | 0 | 0 | 0.0431941 | 0 | 0 | 0.0012266 |
| TCGA-AA-3556-01A | 0.0745938 | 0 | 0.0242232 | 0.1223863 | 0 | 0.1342514 | 0 | 0.0242596 | 0.0633071 | 0 | 0.0163163 | 0.0242853 | 0.0243504 | 0.0265433 | 0.0635663 | 0.2181785 | 0 | 0.046172 | 0 | 0.1279247 | 0 | 0.0096419 |
| TCGA-RU-A8FL-01A | 0.0174176 | 0.0295329 | 0 | 0.1336472 | 0.0052807 | 0.2720155 | 0.0219768 | 0 | 0.0075855 | 0 | 0.0839429 | 0 | 0.0759409 | 0.1638597 | 0.0158269 | 0.0486925 | 0.0105602 | 0 | 0 | 0.1043471 | 0.0093736 | 0 |
| TCGA-AA-3851-01A | 0.0259196 | 0.0539355 | 0 | 0.1327202 | 0 | 0.1018798 | 0 | 0.062059 | 0.092039 | 0 | 0 | 0.0072769 | 0.0284935 | 0.0059551 | 0.0701197 | 0.2364858 | 0.067958 | 0 | 0 | 0.1151579 | 0 | 0 |
| TCGA-CM-5860-01A | 0.0183516 | 0.0072256 | 0 | 0.1069756 | 0 | 0.0902418 | 0.0034699 | 0.0571467 | 0.0528635 | 0 | 0.0082727 | 0 | 0.0200682 | 0.1665961 | 0.0474136 | 0.2588628 | 0.014373 | 0 | 0 | 0.148139 | 0 | 0 |
| TCGA-AA-3534-01A | 0.040252 | 0 | 0.02918 | 0.0547469 | 0 | 0.1847716 | 0 | 0.0782337 | 0.0137171 | 0 | 0.0093115 | 0 | 0 | 0.0881011 | 0.0368376 | 0.371828 | 0.0107456 | 0 | 0.0026988 | 0.0753634 | 0 | 0.0042129 |
| TCGA-AA-A02F-01A | 0.0819244 | 0 | 0.0336157 | 0.1717836 | 0 | 0.0217401 | 0.0117508 | 0.0246632 | 0.024029 | 0 | 0.0289626 | 0 | 0.0096725 | 0.270168 | 0.0569371 | 0.1820746 | 0 | 0 | 0 | 0.0826784 | 0 | 0 |
| TCGA-AA-3979-01A | 0.0909106 | 0 | 0.0519431 | 0.02242 | 0.0652136 | 0 | 0.0586593 | 0.0223136 | 0.0158828 | 0 | 0.0240986 | 0 | 0 | 0.0657859 | 0.0294361 | 0.3447738 | 0 | 0 | 0 | 0.1334016 | 0 | 0.075161 |
| TCGA-DM-A1DA-01A | 0.011163 | 0 | 0.1155945 | 0.2070214 | 0 | 0.0222382 | 0 | 0.0872893 | 0 | 0 | 0 | 0.046918 | 0.0066109 | 0.1369605 | 0.0633335 | 0.2848481 | 0.0014548 | 0 | 0 | 0.0165678 | 0 | 0 |
| TCGA-CM-6162-01A | 0.0393179 | 0 | 0.0061936 | 0.0928305 | 0 | 0.1890751 | 0.0117995 | 0.0093993 | 0.0101073 | 0 | 0.0368897 | 0 | 0.0166891 | 0.0879395 | 0.0697887 | 0.313124 | 0 | 0 | 0.0461914 | 0.0236109 | 0 | 0.0470434 |
| TCGA-AA-3522-01A | 0.10435 | 0 | 0.1045332 | 0.1036974 | 0 | 0.2596233 | 0 | 0.115185 | 0.0476595 | 0 | 0 | 0.0131979 | 0 | 0.0332242 | 0.0344019 | 0.1023773 | 0.0449647 | 0 | 0.0093545 | 0.019807 | 0.0076241 | 0 |
| TCGA-AA-A00U-01A | 0 | 0 | 0.0737101 | 0.1667619 | 0 | 0.1307769 | 0 | 0.2423304 | 0.0587189 | 0 | 0 | 0.033975 | 0.014088 | 0.0407496 | 0.0478084 | 0.1496105 | 0 | 0 | 0 | 0.0414703 | 0 | 0 |
| TCGA-F4-6807-01A | 0.0647598 | 0 | 0.0019618 | 0.1867886 | 0 | 0.1263499 | 0 | 0.0517758 | 0.0600041 | 0 | 0.0206245 | 0 | 0.0060964 | 0.0422422 | 0.1118301 | 0.29208 | 0.0145485 | 0 | 0.019233 | 0.0017053 | 0 | 0 |
| TCGA-AA-3930-01A | 0.045748 | 0 | 0.0426737 | 0.0804634 | 0 | 0.0153496 | 0.0110968 | 0.0763543 | 0.0227817 | 0 | 0.043015 | 0.0019786 | 0.0390977 | 0.1115085 | 0.0438543 | 0.2783741 | 0.0200045 | 0.0105557 | 0.0001317 | 0.0768171 | 0 | 0.0801953 |
| TCGA-AZ-6599-01A | 0.0358775 | 0.0206796 | 0.131851 | 0.1857549 | 0 | 0.2722412 | 0 | 0.1144521 | 0.0365076 | 0 | 0 | 0.0807352 | 0.0364167 | 0 | 0 | 0.0384635 | 0 | 0.0146342 | 0 | 0.0283425 | 0.004044 | 0 |
| TCGA-AA-3947-01A | 0.0022124 | 0 | 0.0006975 | 0.0261422 | 0 | 0.1239757 | 0 | 0.0201525 | 0 | 0.005868 | 0 | 0.0344073 | 0 | 0.0380065 | 0.0716392 | 0.3512418 | 0.0136859 | 0 | 0 | 0.2619872 | 0.0138637 | 0.0361201 |
| TCGA-AA-3841-01A | 0.0649298 | 0.0426798 | 0.0369176 | 0.1691737 | 0 | 0.0427328 | 0.0766554 | 0.0186938 | 0.1066725 | 0 | 0.0255973 | 0 | 0.0551979 | 0.1244152 | 0 | 0.1041249 | 0.0074159 | 0 | 0 | 0.1247935 | 0 | 0 |
| TCGA-AZ-4684-01A | 0.0202206 | 0.0112142 | 0.0300792 | 0.1270685 | 0 | 0.1464634 | 0.0175859 | 0.0436389 | 0.0460935 | 0 | 0.0572649 | 0 | 0 | 0.1073622 | 0.0402394 | 0.1985846 | 0.0103174 | 0.0113107 | 0 | 0.1135134 | 0.0007146 | 0.0183285 |
| TCGA-AA-3814-01A | 0.0307825 | 0 | 0.0296861 | 0.1174523 | 0 | 0.1371034 | 0.0134753 | 0.0776973 | 0.0470556 | 0 | 0 | 0 | 0.0271325 | 0.0438119 | 0.0655273 | 0.2374515 | 0.0586826 | 0 | 0 | 0.0958682 | 0 | 0.0182736 |
| TCGA-CM-6679-01A | 0.017219 | 0.0430078 | 0.0427782 | 0.0744414 | 0.0087163 | 0.0853398 | 0.006051 | 0.0433545 | 0.0345983 | 0 | 0.0705813 | 0 | 0.0330778 | 0.1976888 | 0.0476171 | 0.214753 | 0 | 0 | 0 | 0.0807758 | 0 | 0 |
| TCGA-A6-6649-01A | 0.0320434 | 0.0553156 | 0.0362456 | 0.1928573 | 0 | 0.2187364 | 0 | 0.035212 | 0.0028115 | 0 | 0.0111025 | 0 | 0.0709891 | 0.0538119 | 0.0209515 | 0.1143482 | 0.0172485 | 0.0570946 | 0 | 0.081232 | 0 | 0 |
| TCGA-G4-6297-01A | 0.0409526 | 0.0404367 | 0.007849 | 0.158676 | 0 | 0.135646 | 0 | 0.0619506 | 0.0123911 | 0 | 0 | 0.0145968 | 0.0539433 | 0.0549908 | 0.0774634 | 0.2532671 | 0.0038673 | 0.0055204 | 0 | 0.0772004 | 0 | 0.0012485 |
| TCGA-A6-5656-01B | 0 | 0.0627738 | 0 | 0.01171 | 0 | 0.2967269 | 0.0806001 | 0.0159182 | 0 | 0 | 0.103268 | 0 | 0.01262 | 0.1354801 | 0.051564 | 0.148967 | 0 | 0.0249017 | 0 | 0.0424475 | 0.0130226 | 0 |
| TCGA-DM-A0X9-01A | 0.0070495 | 0 | 0.0549974 | 0.2865288 | 0 | 0.1660962 | 0.0132875 | 0.1402464 | 0.0018803 | 0 | 0 | 0.040535 | 0.0197412 | 0.0049139 | 0.0724467 | 0.1534428 | 0.0245342 | 0 | 0 | 0.0143002 | 0 | 0 |
| TCGA-AA-3553-01A | 0.0562789 | 0 | 0.0821579 | 0.1325168 | 0 | 0.0410205 | 0 | 0.1331314 | 0.0573451 | 0 | 0.0117514 | 0.0293654 | 2.91E-05 | 0.0913682 | 0.1179576 | 0.2088564 | 0.0149675 | 0 | 0.0061927 | 0.0013941 | 0 | 0.015667 |
| TCGA-DM-A0XD-01A | 0.0259098 | 0.0420236 | 0.0818612 | 0.1701246 | 0 | 0 | 0 | 0.0576502 | 0.0215267 | 0 | 0.0166839 | 0.0136757 | 0 | 0.1953582 | 0.0507178 | 0.230854 | 0.0037109 | 0.0045315 | 0 | 0.0830959 | 0 | 0.0022759 |
| TCGA-AA-3511-01A | 0.0567322 | 0 | 0.0466603 | 0.0849474 | 0 | 0.1425156 | 0 | 0.028903 | 0.0538384 | 0 | 0.0207285 | 0.0069878 | 0.0169187 | 0.1458882 | 0.03553 | 0.3033548 | 0 | 0.0076617 | 0.0111088 | 0.0282388 | 0 | 0.0099857 |
| TCGA-A6-A56B-01A | 0 | 0.077711 | 0 | 0.0363639 | 0 | 0.0400654 | 0 | 0.0604176 | 0 | 0 | 0 | 0.0355091 | 0 | 0.289564 | 0.0163625 | 0.3761322 | 0.0034659 | 0 | 0.0644085 | 0 | 0 | 0 |
| TCGA-A6-5667-01A | 0.0467158 | 0 | 0.005729 | 0.1080716 | 0 | 0.0855716 | 0.0335688 | 0.0527777 | 0.0445684 | 0 | 0.0465662 | 0 | 0.037655 | 0.1461645 | 0.05776 | 0.2189407 | 0 | 0.0176705 | 0 | 0.0947495 | 0 | 0.0034906 |
| TCGA-CM-5868-01A | 0.0541872 | 0.0291328 | 0.0212428 | 0.0765639 | 0 | 0.0677402 | 0 | 0.0132181 | 0.0677783 | 0 | 0 | 0 | 0 | 0.2798576 | 0.042872 | 0.3267401 | 0 | 0 | 0 | 0.0206669 | 0 | 0 |
| TCGA-AA-3866-01A | 0.0573269 | 0 | 0.0094041 | 0.0588403 | 0 | 0.0775986 | 0 | 0.0420774 | 0.0958662 | 0 | 0 | 0.0175211 | 0.0189187 | 0.1546448 | 0.0563684 | 0.2991459 | 0.028105 | 0.001925 | 0 | 0.0673727 | 0 | 0.014885 |
| TCGA-AA-A017-01A | 0.0385096 | 0 | 0.1314186 | 0.123386 | 0 | 0.1135294 | 0 | 0.0807255 | 0.047398 | 0 | 0 | 0.0859885 | 0.015992 | 0.1029458 | 0.0168236 | 0.1431869 | 0 | 0.0198989 | 0 | 0.0477312 | 0 | 0.0324661 |
| TCGA-DM-A1D8-01A | 0.0229405 | 0 | 0 | 0.3150798 | 0 | 0 | 0 | 0.0278181 | 0.1312126 | 0 | 0.0461975 | 0.0019842 | 0 | 0.2386742 | 0.0340385 | 0.0777286 | 0.0091339 | 0 | 0 | 0.0951921 | 0 | 0 |
| TCGA-CM-6170-01A | 0.0065416 | 0.0452053 | 0.0071436 | 0.0821375 | 0 | 0.1318392 | 0 | 0.0406563 | 0.039857 | 0 | 0.0282895 | 0.0006763 | 0.0561564 | 0.1870205 | 0.0772407 | 0.1910383 | 0.0176131 | 0 | 0 | 0.0885848 | 0 | 0 |
| TCGA-DM-A28E-01A | 0.0280654 | 0 | 0.1102924 | 0.1054792 | 0 | 0.2529948 | 0.014553 | 0.1492293 | 0 | 0 | 0 | 0.0521121 | 0.0138366 | 0.0177585 | 0.0658264 | 0.1522641 | 0 | 0.036373 | 0 | 0.0012152 | 0 | 0 |
| TCGA-AA-3994-01A | 0.0220071 | 0 | 0.0148468 | 0.0979137 | 0 | 0.2207631 | 0.0006188 | 0.0271505 | 0.0306553 | 0 | 0.0482676 | 0 | 0.0244078 | 0.0694042 | 0.0142486 | 0.3168717 | 0.0017927 | 0 | 0 | 0.0823288 | 0.0198515 | 0.0088718 |
| TCGA-AA-3877-01A | 0.0708243 | 0 | 0.0224264 | 0.126846 | 0 | 0.0550726 | 0 | 0.1035212 | 0.0197464 | 0 | 0.0010454 | 0.0195541 | 0.0001716 | 0.0702452 | 0.0863693 | 0.198925 | 0 | 0 | 0 | 0.0962901 | 0 | 0.1289625 |
| TCGA-WS-AB45-01A | 0.0588256 | 0 | 0.0267612 | 0.010262 | 0 | 0.111961 | 0.0352414 | 0 | 0 | 0 | 0.0660386 | 0 | 0.0861462 | 0.0557401 | 0.0248983 | 0.3489795 | 0 | 0 | 0 | 0.0175071 | 0.0072243 | 0.1504146 |
| TCGA-AA-A00L-01A | 0 | 0.0028497 | 0.1916386 | 0.1093486 | 0.0538045 | 0.0350689 | 0.0506813 | 0.01277 | 0.054478 | 0 | 0 | 0.0427476 | 0.0011422 | 0.1484855 | 0.0163671 | 0.1855517 | 0 | 0.0220704 | 0 | 0.0729959 | 0 | 0 |
| TCGA-CM-5864-01A | 0.0524266 | 0 | 0.0373897 | 0.1233866 | 0 | 0.1806914 | 0.0582654 | 0.0697895 | 0.035151 | 0 | 0.0350578 | 0 | 0.0099672 | 0.0285736 | 0.0415684 | 0.1859195 | 0.0094382 | 0.0038207 | 0 | 0.1285543 | 0 | 0 |
| TCGA-CA-5254-01A | 0.0144299 | 0.0517956 | 0.0722453 | 0.1971374 | 0 | 0.1285436 | 0 | 0.0208864 | 0.0799924 | 0 | 0 | 0.043869 | 0.0452796 | 0.1658284 | 0.0207024 | 0.0945429 | 0.000573 | 0 | 0 | 0.0641743 | 0 | 0 |
| TCGA-D5-6898-01A | 0.0471009 | 0 | 0.0067472 | 0.0537762 | 0 | 0.1392342 | 0 | 0.0325707 | 0.0549498 | 0 | 0.0172002 | 0 | 0.0160468 | 0.3855197 | 0.0317941 | 0.1912148 | 0 | 0 | 0 | 0.0149893 | 0 | 0.0088561 |
| TCGA-AZ-4682-01B | 0.1090136 | 0 | 0.0704232 | 0.1063919 | 0 | 0.2309511 | 0 | 0.0179258 | 0.0027391 | 0 | 0.0398307 | 0.0321995 | 0.0575685 | 0 | 0.0070971 | 0.2342049 | 0.0176777 | 0.0058279 | 0 | 0.0446961 | 0.0234529 | 0 |
| TCGA-AA-3696-01A | 0 | 0.046707 | 0.0551553 | 0.0564486 | 0 | 0.3061542 | 0.0181335 | 0 | 0 | 0 | 0.0341462 | 0 | 0.1006596 | 0 | 0.019446 | 0.2612931 | 0 | 0.003546 | 0 | 0.0983104 | 0 | 0 |
| TCGA-DM-A280-01A | 0.0345308 | 0 | 0 | 0.0731874 | 0 | 0.0159339 | 0 | 0.0173716 | 0.0168493 | 0 | 0 | 0.0605426 | 0.0369471 | 0.3127063 | 0 | 0.2011114 | 0 | 0.0107635 | 0 | 0.1968307 | 0 | 0.0232256 |
| TCGA-AA-A00O-01A | 0.0105817 | 0 | 0.0199639 | 0.0696697 | 0 | 0.0807325 | 0 | 0.1041675 | 0.0594889 | 0 | 0.0577478 | 0 | 0 | 0.2755156 | 0.0529283 | 0.2167415 | 0 | 0 | 0 | 0.0524625 | 0 | 0 |
| TCGA-D5-6531-01A | 0.0158205 | 0 | 0.0146009 | 0.2741094 | 0 | 0 | 0.0234073 | 0.051962 | 0.1035883 | 0 | 0 | 0.0642118 | 0.0442289 | 0.0494054 | 0.0917828 | 0.1856318 | 0.0044956 | 0 | 0.0463431 | 0 | 0.0304123 | 0 |
| TCGA-CK-4948-01B | 0.0110476 | 0.0351063 | 0 | 0.037282 | 0 | 0.039406 | 0.0281364 | 0.0755133 | 0.0602216 | 0 | 0.0406053 | 0 | 0.0105194 | 0.2312284 | 0 | 0.2561862 | 0 | 0.058235 | 0 | 0.1101577 | 0 | 0.0063548 |
| TCGA-DM-A1HA-01A | 0.0008778 | 0 | 0.0039076 | 0.3655868 | 0 | 0 | 0.0037314 | 0.1935296 | 0 | 0 | 0 | 0.1217735 | 0.0247375 | 0 | 0.1720555 | 0.1130155 | 0 | 0 | 0.0007848 | 0 | 0 | 0 |
| TCGA-5M-AAT5-01A | 0.263198 | 0 | 0.3114174 | 0.0449897 | 0 | 0.0700217 | 0.0506676 | 0.0838517 | 0 | 0 | 0.0124724 | 0 | 0.0060346 | 0.0219807 | 0 | 0.0898329 | 0.004142 | 0.0071282 | 0 | 0.0342632 | 0 | 0 |
| TCGA-CM-6674-01A | 0.043073 | 0 | 0.0090669 | 0.1117264 | 0 | 0.1329611 | 0 | 0.0495679 | 0.0488337 | 0 | 0 | 0.0965193 | 0.0052423 | 0.1269589 | 0.1171703 | 0.2006448 | 0.0058452 | 0 | 0.0523904 | 0 | 0 | 0 |
| TCGA-F4-6809-01A | 0.0649671 | 0.072566 | 0 | 0.1954049 | 0 | 0.1056143 | 0 | 0.0773119 | 0.0655379 | 0 | 0 | 0.0041872 | 0.0608883 | 0.0791651 | 0.0740346 | 0.1131804 | 6.22E-06 | 0 | 0 | 0.0723789 | 0.0147572 | 0 |
| TCGA-CM-4748-01A | 0.1703724 | 0 | 0.0308131 | 0.078074 | 0 | 0.0465023 | 0.010378 | 0.0543034 | 0.0012472 | 0 | 0.0748009 | 0 | 0.0776112 | 0.1147752 | 0.0110443 | 0.135978 | 0 | 0 | 0 | 0.1940999 | 0 | 0 |
| TCGA-AA-3858-01A | 0.0274369 | 0 | 0.0766656 | 0.0699411 | 0 | 0.0983568 | 0 | 0.0761037 | 0.1043543 | 0 | 0.0229109 | 0 | 0 | 0.1100076 | 0.0844796 | 0.2630504 | 0.0035749 | 0 | 0 | 0.0630617 | 0 | 5.64E-05 |
| TCGA-A6-6780-01B | 0.0502885 | 0 | 0 | 0.0376125 | 0 | 0.284036 | 0.0131781 | 0.0046308 | 0 | 0 | 0.0147695 | 0.0119349 | 0 | 0.0382631 | 0.0851546 | 0.187667 | 0 | 0.011281 | 0 | 0.2169305 | 0 | 0.0442536 |
| TCGA-AA-3662-01A | 0.0155293 | 0.0588884 | 0.0404731 | 0.0689894 | 0.0491159 | 0 | 0.0699032 | 0.0213627 | 0 | 0 | 0.0564833 | 0.0224804 | 0.0460155 | 0.0246465 | 0.0433331 | 0.3142235 | 0.0022623 | 0.0019805 | 0 | 0.164313 | 0 | 0 |
| TCGA-AM-5820-01A | 0.0102344 | 0.0174244 | 0 | 0.1507169 | 0 | 0.0830479 | 0 | 0.0077211 | 0.0005073 | 0 | 0 | 0.0071176 | 0.0479809 | 0 | 0.0077437 | 0.4749385 | 0.0331572 | 0 | 0 | 0.1398374 | 0.0195726 | 0 |
| TCGA-AA-A02K-01A | 0 | 0.0814064 | 0.1146163 | 0.1340472 | 0 | 0 | 0 | 0.0343518 | 0.0410269 | 0 | 0.0362434 | 0.0058707 | 0.0032017 | 0.2583964 | 0.0074533 | 0.2042191 | 0 | 0 | 0 | 0.0512089 | 0 | 0.0279578 |
| TCGA-G4-6310-01A | 0 | 0.0723881 | 0.0138418 | 0.0496093 | 0 | 0.123071 | 0.0129154 | 0.0391742 | 0 | 0 | 0.1323096 | 0 | 0 | 0.3127589 | 0.0166686 | 0.1971644 | 0 | 0 | 0 | 0.0300987 | 0 | 0 |
| TCGA-AD-6889-01A | 0 | 0 | 0.035472 | 0.0329754 | 0 | 0.2189307 | 0.0003485 | 0.0913972 | 0.0145237 | 0 | 0.030974 | 0 | 0 | 0.1374544 | 0.0326119 | 0.1802125 | 0 | 0.0012693 | 0 | 0.200906 | 0 | 0.0229242 |
| TCGA-AA-3872-01A | 0.0826184 | 0 | 0.005774 | 0.0650432 | 0 | 0.0964591 | 0 | 0.0907342 | 0.0489645 | 0 | 0 | 0.000989 | 0.0025761 | 0.1715848 | 0.0530152 | 0.2293885 | 0.0073183 | 0 | 0.0348687 | 0.1052353 | 0 | 0.0054308 |
| TCGA-AA-A01R-01A | 0.0150498 | 0.0251434 | 0 | 0.2324581 | 0 | 0 | 0 | 0.1342573 | 0.1543093 | 0 | 0 | 0.1627111 | 0 | 0.0941339 | 0.0314534 | 0.1226931 | 0.0147248 | 0 | 0.0130657 | 0 | 0 | 0 |
| TCGA-AA-3975-01A | 0 | 0.0528596 | 0.0353781 | 0.0818613 | 0 | 0.0342332 | 0.0550795 | 0.0138529 | 0.0562031 | 0 | 0.0589291 | 0 | 0.0369033 | 0.181176 | 0 | 0.3024093 | 0.0074701 | 0.0169018 | 0 | 0.0667429 | 0 | 0 |
| TCGA-AD-6964-01A | 0.0174208 | 0 | 0.0079445 | 0.1981895 | 0 | 0.0699489 | 0 | 0.1424998 | 0.0479197 | 0 | 0 | 0.0185796 | 0.0160244 | 0.0575597 | 0.1069482 | 0.2443371 | 0.032941 | 0 | 0 | 0.0396868 | 0 | 0 |
| TCGA-AA-3562-01A | 0.0834164 | 0 | 0.0450341 | 0.0677082 | 0 | 0.1010794 | 0 | 0.0880025 | 0.0501048 | 0 | 0 | 0.0104077 | 0 | 0.0992618 | 0.0524752 | 0.3241757 | 0.0276287 | 0 | 0.0229176 | 0.0221181 | 0.0011 | 0.0045699 |
| TCGA-AZ-4616-01A | 0 | 0.0324692 | 0.0405746 | 0.3168144 | 0 | 0 | 0 | 0.1598746 | 0.0364191 | 0 | 0.0138483 | 0.0298958 | 0.0157781 | 0.0676367 | 0.158873 | 0.1048063 | 0 | 0 | 0 | 0.0230099 | 0 | 0 |
| TCGA-D5-6931-01A | 0 | 0.0439584 | 0.0222279 | 0.1214704 | 0 | 0.1792123 | 0 | 0.1186945 | 0.0080462 | 0 | 0.0241247 | 0 | 0 | 0.0178257 | 0.0568076 | 0.2704674 | 0.000671 | 0 | 0.0533876 | 0.0831064 | 0 | 0 |
| TCGA-D5-6924-01A | 0.018545 | 0 | 0.0668452 | 0.1003198 | 0 | 0.1387686 | 0 | 0.0637258 | 0.0067479 | 0 | 0.0014683 | 0.0157125 | 0.0024881 | 0.1322854 | 0.0871664 | 0.2764173 | 0 | 0 | 0.0691134 | 0 | 0 | 0.0203962 |
| TCGA-AA-3860-01A | 0.0429371 | 0 | 0.0432455 | 0.0673906 | 0 | 0.2608757 | 0 | 0.104201 | 0.0210679 | 0 | 0.0409971 | 0 | 0 | 0.0751909 | 0.0744871 | 0.179785 | 0 | 0 | 0 | 0.0848763 | 0 | 0.0049457 |
| TCGA-A6-5665-01B | 0 | 0.0254873 | 0.0581525 | 0.0310901 | 0 | 0.1829823 | 0 | 0.0610364 | 0 | 0 | 0.0741534 | 0.0126891 | 0 | 0.1635799 | 0.0540986 | 0.177141 | 0 | 0.0057092 | 0.0363398 | 0 | 0 | 0.1175404 |
| TCGA-QG-A5YW-01A | 0.1175227 | 0 | 0.2215579 | 0.0347258 | 0 | 0.1897377 | 0.0099394 | 0.0808372 | 0 | 0 | 0.0266234 | 0 | 0 | 0.0851356 | 0.0244501 | 0.1428185 | 0 | 6.03E-05 | 0 | 0.0665915 | 0 | 0 |
| TCGA-D5-6540-01A | 0.003884 | 0 | 0.0132823 | 0.1435372 | 0 | 0 | 0 | 0.0934043 | 0.0157397 | 0 | 0.0208529 | 0.0203072 | 0 | 0.3206395 | 0.060333 | 0.105545 | 0 | 0 | 0 | 0.1595589 | 0 | 0.042916 |
| TCGA-CA-6718-01A | 0.0694224 | 0 | 0.0758907 | 0.1248795 | 0 | 0.0862247 | 0.0054363 | 0.1088755 | 0.0165202 | 0 | 0.0483337 | 0.0324216 | 0 | 0.0530737 | 0.1630757 | 0.1962745 | 0.0019552 | 0 | 0.0057745 | 0 | 0 | 0.011842 |
| TCGA-G4-6323-01A | 0.4350935 | 0.0624512 | 0 | 0.1000034 | 0 | 0.1694725 | 0 | 0.0836356 | 0.0026034 | 0 | 0.0049086 | 0 | 0.008094 | 0 | 0.0110657 | 0.1098888 | 0 | 0.0073071 | 0.0054762 | 0 | 0 | 0 |
| TCGA-AA-3986-01A | 0.0352273 | 0.0094035 | 0.1367035 | 0.1763427 | 0 | 0.0833183 | 0 | 0.0500554 | 0.1325462 | 0 | 0 | 0.0119462 | 0.001863 | 0.0404666 | 0.1092449 | 0.1579595 | 0.0019039 | 0 | 0 | 0.0423215 | 0 | 0.0106974 |
| TCGA-AA-3520-01A | 0.0128581 | 0 | 0.0187399 | 0.0987217 | 0 | 0.2190138 | 0 | 0.0270915 | 0.019197 | 0 | 0 | 0.0119028 | 0.0028266 | 0 | 0.0932867 | 0.3280871 | 0.0091143 | 0 | 0 | 0.1331436 | 0.0260169 | 0 |
| TCGA-A6-5662-01A | 0.0307511 | 0.0121606 | 0.0023907 | 0.0575733 | 0 | 0.1844361 | 0.0585085 | 0 | 0 | 0 | 0.0555431 | 0.0162297 | 0.0675721 | 0.0305742 | 0 | 0.3374821 | 0.0582334 | 0 | 0.0574361 | 0 | 0.0311089 | 0 |
| TCGA-AA-3831-01A | 0 | 0.0058099 | 0.0894808 | 0.0909961 | 0 | 0.2804048 | 0 | 0.0655245 | 0.0709728 | 0 | 0.0086021 | 0.0019829 | 0.0193653 | 0 | 0.0379871 | 0.2255919 | 0.0303039 | 0 | 0 | 0.0648116 | 0.0024047 | 0.0057616 |
| TCGA-AA-A02H-01A | 0 | 0.0912952 | 0.0801961 | 0.1962357 | 0 | 0.1187437 | 0 | 0.0739364 | 0 | 0 | 0.0547844 | 0 | 0.0273932 | 0.0435601 | 0.0249917 | 0.247521 | 0.0092448 | 0 | 0 | 0.0320976 | 0 | 0 |
| TCGA-CM-6676-01A | 0.0107578 | 0.0274178 | 0.008343 | 0.0746293 | 0.0131329 | 0.0510153 | 0.0731083 | 0.011174 | 0.0153185 | 0 | 0.1136179 | 0 | 0.0377014 | 0.3276419 | 0.0135508 | 0.1116258 | 0.0206539 | 0 | 0 | 0.0903113 | 0 | 0 |
| TCGA-AD-6899-01A | 0.0471454 | 0 | 0.0232414 | 0.1175898 | 0 | 0.1946907 | 0 | 0.0310436 | 0.0606918 | 0 | 0 | 0.0038036 | 0 | 0.1702459 | 0.1067342 | 0.2123055 | 0.0023194 | 0 | 0 | 0.0110552 | 0.0191334 | 0 |
| TCGA-AA-3685-01A | 0.0283757 | 0.0010463 | 0.0200941 | 0.1333148 | 0 | 0.1500505 | 0.0398026 | 0.1107648 | 0.0288404 | 0 | 0 | 0.0167582 | 0.0119978 | 0.0214883 | 0.1389669 | 0.2417965 | 0.0127576 | 0 | 0.0058866 | 0.007485 | 0.0257918 | 0.004782 |
| TCGA-CK-5915-01A | 0.0213047 | 0 | 0 | 0.0887938 | 0 | 0.4267398 | 0.1711274 | 0 | 0 | 0 | 0.0487499 | 0 | 0.0675654 | 0.0467872 | 0 | 0 | 0.0072821 | 0.0427281 | 0 | 0.0624567 | 0.016465 | 0 |
| TCGA-AD-6548-01A | 0.0896867 | 0 | 0.0378048 | 0.1097543 | 0 | 0.1243951 | 0 | 0.0677236 | 0 | 0 | 0 | 0.0369599 | 0.0182697 | 0.1636706 | 0.095947 | 0.1993766 | 0 | 0 | 0 | 0.0549366 | 0 | 0.001475 |
| TCGA-AA-3548-01A | 0.0403454 | 0.0164349 | 0 | 0.195301 | 0 | 0.0834099 | 0 | 0.0363668 | 0.1835757 | 0 | 0.015803 | 0.0179706 | 0.0362077 | 0.0192453 | 0.0521143 | 0.2158352 | 0.0238552 | 0 | 0 | 0.063535 | 0 | 0 |
| TCGA-A6-6141-01A | 0.0148637 | 0.0358882 | 0.0166852 | 0.1777872 | 0 | 0.1608703 | 0 | 0.1365325 | 0.1096403 | 0 | 0 | 0.0553112 | 0.017486 | 0.0129531 | 0.0895273 | 0.1268392 | 0.0130289 | 0 | 0.0325867 | 0 | 0 | 0 |
| TCGA-F4-6461-01A | 0.4025406 | 0 | 0 | 0.0830377 | 0 | 0.1273555 | 0 | 0.0463127 | 0.0500568 | 0 | 0.0272535 | 0 | 0.0164164 | 0.0733688 | 0.039377 | 0.0970726 | 0 | 0 | 0 | 0.0372084 | 0 | 0 |
| TCGA-F4-6854-01A | 0.0568032 | 0 | 0.0180963 | 0.1473655 | 0 | 0.1235802 | 0 | 0.0924366 | 0.0281005 | 0 | 0 | 0.0366898 | 0.0190711 | 0.1435422 | 0.1042821 | 0.2217416 | 0 | 0 | 0.0082909 | 0 | 0 | 0 |
| TCGA-F4-6856-01A | 0.0774481 | 0 | 0.0239523 | 0.1630569 | 0 | 0.2273181 | 0 | 0.0431547 | 0.1299879 | 0 | 0.0211689 | 0.0368121 | 0.0364524 | 0.0815954 | 0.0341725 | 0.0728363 | 0.0007687 | 0.0045411 | 0 | 0.0467347 | 0 | 0 |
| TCGA-AY-A54L-01A | 0.0268743 | 0 | 0.1797987 | 0.1247236 | 0 | 0.2678405 | 0.0536773 | 0.0201209 | 0 | 0 | 0.0345623 | 0.0070415 | 0.0286996 | 0.0485729 | 0 | 0 | 0.0241231 | 0.0155607 | 0 | 0.162207 | 0.0061976 | 0 |
| TCGA-AA-3538-01A | 0.0155582 | 0.0239051 | 0.0432543 | 0.0232962 | 0 | 0.0646602 | 0.0149872 | 0.0422745 | 0.0328348 | 0 | 0.0609247 | 0.0041139 | 0 | 0.1384079 | 0.0028214 | 0.3580397 | 0.0275384 | 0.0148233 | 0 | 0.0801011 | 0 | 0.0524591 |
| TCGA-AA-3842-01A | 0.0486086 | 0 | 0.004673 | 0.1876561 | 0 | 0 | 0.0009408 | 0.1253028 | 0.0261533 | 0 | 0 | 0.0282582 | 0 | 0.2158556 | 0.0714173 | 0.2456275 | 0 | 0 | 0 | 0.0455068 | 0 | 0 |
| TCGA-AZ-4308-01A | 0.0083755 | 0 | 0.0865999 | 0.0683475 | 0 | 0.0796718 | 0 | 0.1193588 | 0.0942307 | 0 | 0 | 0.0248444 | 0 | 0.2034394 | 0.0545426 | 0.1542922 | 0 | 0 | 0 | 0.0839734 | 0 | 0.0223238 |
| TCGA-CK-6746-01A | 0.024235 | 0 | 0.0016191 | 0.1897603 | 0 | 0 | 0.1582686 | 0.047743 | 0.0077098 | 0 | 0.015071 | 0.0290465 | 0.0062278 | 0 | 0.1724491 | 0.3478698 | 0 | 0 | 0 | 0 | 0 | 0 |
| TCGA-AA-3509-01A | 0.031942 | 0 | 0.0513327 | 0.0672808 | 0 | 0.1663759 | 0.0162562 | 0.0226241 | 0.0505673 | 0 | 0.0520065 | 0 | 0.0585241 | 0.0591458 | 0.0976937 | 0.2687692 | 0.0101249 | 0 | 0 | 0.0419977 | 0.0053591 | 0 |
| TCGA-AA-3680-01A | 0.0579505 | 0.0333847 | 0.0316964 | 0.1668321 | 0 | 0.1901106 | 0 | 0.0636081 | 0.0755339 | 0 | 0 | 0.0192106 | 0.0395402 | 0 | 0.0095265 | 0.2211321 | 0 | 0 | 0 | 0.0914744 | 0 | 0 |
| TCGA-A6-5656-01A | 0.0113925 | 0.0212087 | 0.0037695 | 0.0288293 | 0 | 0.0269103 | 0.0375731 | 0.0291847 | 0.0228437 | 0 | 0.0931319 | 0 | 0 | 0.5349628 | 0.0308753 | 0.0374396 | 0.0311248 | 0 | 0 | 0.0907538 | 0 | 0 |
| TCGA-CM-6680-01A | 0.0231528 | 0 | 0.0282303 | 0.071682 | 0 | 0.2274583 | 0.0133185 | 0.0385323 | 0.0696925 | 0 | 0.0283397 | 0 | 0 | 0.0888665 | 0.0563485 | 0.3141637 | 0.0118554 | 0 | 0 | 0.0283595 | 0 | 0 |
| TCGA-A6-6650-01B | 0.078269 | 0 | 0.183132 | 0 | 0 | 0.3436568 | 0 | 0.0128923 | 0 | 0 | 0.0530713 | 0 | 0.0067943 | 0.0199601 | 0.0581163 | 0.1515034 | 0 | 0.0136728 | 0 | 0.0780499 | 0.0008816 | 0 |
| TCGA-A6-6140-01A | 0.0316045 | 0 | 0.0494531 | 0.1870384 | 0 | 0.1709706 | 0.0395085 | 0.0553284 | 0.0473128 | 0 | 0.0842634 | 0 | 0.0379172 | 0.0804186 | 0.0190082 | 0.1038429 | 0 | 0.0116242 | 0 | 0.0792378 | 0 | 0.0024715 |
| TCGA-AA-A024-01A | 0 | 0.0369214 | 0.0329177 | 0.1259641 | 0 | 0.0619224 | 0 | 0.1637465 | 0.0016169 | 0 | 0 | 0 | 0 | 0.2617441 | 0 | 0 | 0.0102711 | 0.0077556 | 0 | 0.2608026 | 0.0363376 | 0 |
| TCGA-A6-2682-01A | 0.0074741 | 0.0118681 | 0.0179903 | 0.0244534 | 0.003358 | 0.0102804 | 0.0481051 | 0 | 0.0457334 | 0 | 0.1199719 | 0 | 0 | 0.3160144 | 0 | 0.0473709 | 0.0045056 | 0 | 0 | 0.3201922 | 0 | 0.0226821 |
| TCGA-CM-6675-01A | 0 | 0.0299974 | 0.0726758 | 0.143005 | 0 | 0.0974913 | 0 | 0.0976832 | 0.0665706 | 0 | 0.0139097 | 0.0216156 | 0.0109318 | 0.0437106 | 0.0690574 | 0.2880989 | 0.0141826 | 0 | 0 | 0.03107 | 0 | 0 |
| TCGA-DM-A1D7-01A | 0.0936887 | 0 | 0.1081933 | 0.093422 | 0 | 0.1372955 | 0 | 0.1621826 | 0.0402422 | 0 | 0 | 0.0286406 | 0.0138716 | 0.0207782 | 0.0346831 | 0.2554218 | 0 | 0.0115804 | 0 | 0 | 0 | 0 |
| TCGA-F4-6460-01A | 0.0556984 | 0.0312447 | 0.0166827 | 0.101341 | 0 | 0.1882957 | 0.0200379 | 0 | 0.0331763 | 0 | 0.0885083 | 0 | 0.0574904 | 0.1170966 | 0.065909 | 0.1508125 | 0.0559059 | 0 | 0 | 0.0178006 | 0 | 0 |
| TCGA-AA-3655-01A | 0.0080491 | 0.0551248 | 0.0278139 | 0.1518302 | 0 | 0.2037132 | 0.0010323 | 0 | 0.0567659 | 0 | 0 | 0.0211755 | 0.0454643 | 0.120772 | 0.0408147 | 0.1763941 | 0.0040959 | 0.0166211 | 0 | 0.0648331 | 0.0055 | 0 |
| TCGA-CM-6169-01A | 0.0318834 | 0 | 0.0060735 | 0.0764445 | 0 | 0.1320693 | 0.017177 | 0.0291718 | 0.0416881 | 0 | 0.0113874 | 0 | 0.0151218 | 0.1319383 | 0.083071 | 0.3607568 | 0.0017969 | 0 | 0.0534607 | 0 | 9.63E-05 | 0.0078633 |
| TCGA-AA-3952-01A | 0.0876996 | 0 | 0.020894 | 0.0503715 | 0 | 0.1052349 | 0 | 0.0700695 | 0.0252124 | 0 | 0 | 0.0031872 | 0 | 0.1074278 | 0.0520151 | 0.3989304 | 0 | 0 | 0 | 0.0271925 | 0 | 0.0517651 |
| TCGA-A6-5666-01A | 0.0702946 | 0 | 0.0059432 | 0.055106 | 0 | 0.0396142 | 0 | 0.017134 | 0.0165136 | 0 | 0 | 0 | 0.0178822 | 0.2627376 | 0.0536157 | 0.3096337 | 0 | 0 | 0 | 0.1032183 | 0 | 0.0483069 |
| TCGA-T9-A92H-01A | 0 | 0.0785177 | 0.1281008 | 0.099716 | 0 | 0.147349 | 0 | 0.1309274 | 0 | 0 | 0 | 0.0537724 | 0.0033688 | 0.1267496 | 0.0309271 | 0.1937954 | 0 | 0 | 0 | 0.0067759 | 0 | 0 |
| TCGA-A6-5665-01A | 0.056884 | 0 | 0.0254692 | 0.2645097 | 0 | 0.1061888 | 0.0230084 | 0.120783 | 0.0010222 | 0 | 0.0516559 | 0 | 0.022076 | 0.0551117 | 0.0397878 | 0.114849 | 0 | 0.0003078 | 0 | 0.1108889 | 0.0074575 | 0 |
| TCGA-AA-3846-01A | 0.0119962 | 0.0359584 | 0.0588024 | 0.1024757 | 0 | 0.1456654 | 0.0886339 | 0.0784295 | 0 | 0 | 0.060052 | 0 | 0.0421953 | 0 | 0.0091243 | 0.2384227 | 0.0203545 | 0.0253878 | 0 | 0.0696649 | 0.0128369 | 0 |
| TCGA-CK-4950-01A | 0.023587 | 0.007959 | 0.1541874 | 0.1025589 | 0 | 0.1780312 | 0 | 0.1161319 | 0.0106568 | 0 | 0 | 0.0177696 | 0 | 0.0708757 | 0.024797 | 0.2273421 | 0 | 0 | 0 | 0.0284265 | 0.0269683 | 0.0107088 |
| TCGA-F4-6570-01A | 0.0081646 | 0.0060882 | 0.0086069 | 0.2159945 | 0 | 0.0740934 | 0.0009189 | 0.047035 | 0.0429899 | 0 | 0.003504 | 0.0707672 | 0.0389172 | 0.0410284 | 0.091971 | 0.3229049 | 0.0088011 | 0 | 0.0182147 | 0 | 0 | 0 |
| TCGA-AZ-6600-01A | 0.1292074 | 0 | 0.0382859 | 0.0862192 | 0 | 0.1724866 | 0 | 0.0173849 | 0.072414 | 0 | 0 | 0 | 0 | 0.157469 | 0.0505691 | 0.2076337 | 0.0096492 | 0 | 0.0463444 | 0 | 0 | 0.0123366 |
| TCGA-AA-A00J-01A | 0 | 0.0022147 | 0.0367401 | 0.099868 | 0 | 0.0311884 | 0.0112753 | 0.0615857 | 0.0364654 | 0 | 0.0353934 | 0 | 0 | 0.1947375 | 0.0584704 | 0.280651 | 0 | 0 | 0 | 0.1436297 | 0 | 0.0077805 |
| TCGA-AA-3667-01A | 0.0084596 | 0.042477 | 0.050911 | 0.0621955 | 0.0559352 | 0 | 0.0977583 | 0 | 0.0091756 | 0 | 0.1156879 | 0 | 0.0579813 | 0.1007177 | 0 | 0.1232787 | 0.0260683 | 0 | 0 | 0.2390577 | 0 | 0.0102961 |
| TCGA-A6-5661-01A | 0.0717095 | 0 | 0.0184777 | 0.105121 | 0 | 0.2272138 | 0.0376048 | 0.1257463 | 0 | 0 | 0.0334112 | 0 | 0.0189203 | 0.0520446 | 0.0439359 | 0.1346394 | 0.0029569 | 0.0180401 | 0 | 0.1066998 | 0.0034787 | 0 |
| TCGA-AA-3554-01A | 0.0079906 | 0.018818 | 0 | 0.0709514 | 0 | 0.0524577 | 0 | 0.037416 | 0.0449569 | 0 | 0.0808279 | 0 | 0 | 0.2644302 | 0.0156482 | 0.1550926 | 0.0016853 | 0 | 0 | 0.2237097 | 0.0002535 | 0.0257618 |
| TCGA-AZ-6606-01A | 0.0412445 | 0 | 0.0664016 | 0.0985246 | 0 | 0.0816827 | 0 | 0.1158178 | 0.0655559 | 0 | 0 | 0.031666 | 0 | 0.1766478 | 0.0145823 | 0.2685917 | 0 | 0.0372775 | 0 | 0.0020077 | 0 | 0 |
| TCGA-AA-3852-01A | 0.0008308 | 0.0325323 | 0.0170891 | 0.1073157 | 0 | 0.1388782 | 0 | 0.0695681 | 0.0855258 | 0 | 0.0192772 | 0 | 0.0203187 | 0.1211751 | 0.0146203 | 0.262365 | 0.0055582 | 0.0058875 | 0 | 0.0571401 | 0.0208764 | 0.0210417 |
| TCGA-D5-6922-01A | 0.0132249 | 0 | 0.0381278 | 0.0877069 | 0 | 0.0777281 | 0 | 0.0327405 | 0.0714496 | 0 | 0.014032 | 0.0035285 | 0 | 0.1155302 | 0.1168018 | 0.3511338 | 0 | 0 | 0.0760641 | 0 | 0 | 0.0019316 |
| TCGA-A6-6137-01A | 0 | 0.0456239 | 0.0299071 | 0.0499187 | 0 | 0.3031506 | 0 | 0.0127546 | 0.1736688 | 0 | 0 | 0 | 0.0360902 | 0.0587096 | 0.0200276 | 0.1582389 | 0 | 0.0793292 | 0 | 0.0325809 | 0 | 0 |
| TCGA-A6-2672-01A | 0.0081453 | 0 | 0.0083167 | 0.1697463 | 0 | 0.0367475 | 0.0247833 | 0.0967061 | 0.052375 | 0 | 0.0536594 | 0 | 0 | 0.1027769 | 0.0890712 | 0.1572746 | 0.0033069 | 0.0019423 | 0 | 0.1730966 | 0 | 0.022052 |
| TCGA-AA-3495-01A | 0.0034672 | 0.002406 | 0.1418694 | 0.1250876 | 0 | 0.2102563 | 0 | 0.1029693 | 0.038717 | 0 | 0 | 0.0195113 | 0.0091372 | 0.0591811 | 0.0548346 | 0.1993923 | 0 | 0.0005603 | 0.0168827 | 0.0115769 | 0 | 0.0041509 |
| TCGA-A6-6650-01A | 0.0460992 | 0 | 0.1209173 | 0.0546211 | 0 | 0.147745 | 0.049784 | 0.0702892 | 0.0294739 | 0 | 0.0667908 | 0 | 0.0406468 | 0.0397866 | 0.0582192 | 0.1979453 | 0 | 0.0019638 | 0 | 0.0757177 | 0 | 0 |
| TCGA-D5-7000-01A | 0.0765711 | 0 | 0.0309751 | 0.1043205 | 0 | 0.1232312 | 0 | 0.0919578 | 0.0012216 | 0 | 0.0170783 | 0 | 0 | 0.108336 | 0.0468945 | 0.2172278 | 0.0051017 | 0 | 0.0244308 | 0.062293 | 0.047566 | 0.0427945 |
| TCGA-A6-2678-01A | 0.0530826 | 0 | 0.0080828 | 0.1059305 | 0 | 0.1332485 | 0.0734396 | 0.0656695 | 0.0088604 | 0 | 0 | 0.0355994 | 0.0159817 | 0.0729696 | 0.0977121 | 0.2611639 | 0.0118552 | 0 | 0 | 0.0564041 | 0 | 0 |
| TCGA-AA-3976-01A | 0.0435402 | 0.0485312 | 0 | 0.132258 | 0 | 0.1337542 | 0.0052909 | 0.0637748 | 0.0585601 | 0 | 0.040782 | 0 | 0.0503687 | 0 | 0.0279701 | 0.2362734 | 0.0759863 | 0 | 0 | 0.0829101 | 0 | 0 |
| TCGA-AA-3811-01A | 0.0027837 | 0.0385547 | 0.0795015 | 0.1826458 | 0 | 0.1460979 | 0 | 0.0866706 | 0.0234211 | 0 | 0.0073104 | 0 | 0.0328624 | 0 | 0.0449055 | 0.1646394 | 0.0039599 | 0.0117932 | 0 | 0.1686999 | 0.0061542 | 0 |
| TCGA-AA-A01V-01A | 0.0514023 | 0 | 0.0456167 | 0.1303858 | 0 | 0.0899273 | 0.0225983 | 0.232131 | 0.1782966 | 0 | 0.0098559 | 0 | 0 | 0 | 0.0244419 | 0.1330957 | 0 | 0.0074405 | 0 | 0.0748081 | 0 | 0 |
| TCGA-AD-A5EJ-01A | 0 | 0.0114812 | 0.027184 | 0.0657498 | 0 | 0.1401075 | 0 | 0.0481025 | 0.0224793 | 0 | 0.1076858 | 0 | 0 | 0.2251758 | 0.0325553 | 0.1957869 | 0 | 0 | 0 | 0.1155372 | 0 | 0.0081547 |
| TCGA-A6-2674-01B | 0 | 0.0237979 | 0 | 0 | 0 | 0.1960278 | 0 | 0 | 0 | 0 | 0.0565074 | 0 | 0 | 0.2249871 | 0.0167154 | 0.1030527 | 0 | 0 | 0 | 0.2151029 | 0.0645242 | 0.0992846 |
| TCGA-AA-3819-01A | 0.0531779 | 0.0156871 | 0.0512371 | 0.0757714 | 0 | 0.162513 | 0 | 0.0405841 | 0.0414603 | 0 | 0 | 0 | 0.0154323 | 0 | 0 | 0.2603556 | 0.0367308 | 0.0400504 | 0 | 0.1737174 | 0 | 0.0332827 |
| TCGA-CM-6172-01A | 0.0139309 | 0 | 0.0389954 | 0.0631691 | 0 | 0.2273893 | 0.0348675 | 0.0020191 | 5.19E-05 | 0 | 0.0193498 | 0 | 0.0046079 | 0.1056419 | 0.0022271 | 0.365859 | 0.017935 | 0.0331981 | 0.0554026 | 0 | 0 | 0.0153552 |
| TCGA-AA-A02Y-01A | 0 | 0.002888 | 0.0084489 | 0.2355308 | 0 | 0.1260703 | 0.0247067 | 0.1587705 | 0.0784676 | 0 | 0 | 0.0516108 | 0.0156933 | 0 | 0.1344154 | 0.0526995 | 0.0080132 | 0.006606 | 0 | 0.0960791 | 0 | 0 |
| TCGA-AZ-6603-01A | 0.0610167 | 0 | 0.0499294 | 0.0604788 | 0 | 0.1510096 | 0 | 0.0559211 | 0.0412588 | 0 | 0.0015684 | 0.0184744 | 0.0348452 | 0.088432 | 0.164313 | 0.2299213 | 0.0043913 | 0 | 0.0340261 | 0 | 0 | 0.0044139 |
| TCGA-AA-A022-01A | 0 | 0.0127399 | 0.039282 | 0.4333107 | 0 | 0 | 0.0545223 | 0.0908509 | 0.0965177 | 0 | 0 | 0.0633399 | 0.0304546 | 0 | 0.0266753 | 0.0756729 | 0.0082914 | 0.0003278 | 0 | 0.0680145 | 0 | 0 |
| TCGA-NH-A8F8-01A | 0.1013306 | 0 | 0.1571184 | 0.0443298 | 0 | 0.0368536 | 0 | 0.0788361 | 0.0135913 | 0 | 0.0242014 | 0 | 0 | 0.1205184 | 0.0785626 | 0.2891907 | 0 | 0 | 0.0014196 | 0.0363206 | 0.009367 | 0.0083598 |
| TCGA-G4-6626-01A | 0.0749374 | 0.077491 | 0 | 0.2716939 | 0 | 0.0685234 | 0.1172743 | 0.0225765 | 0 | 0 | 0 | 0 | 0.0151959 | 0.1102534 | 0.0277305 | 0 | 0 | 0.033447 | 0 | 0.1540431 | 0.0268335 | 0 |
| TCGA-AA-3875-01A | 0.104722 | 0 | 0.0459816 | 0.1148071 | 0 | 0.0356727 | 0 | 0.1269719 | 0.0304112 | 0 | 0 | 0.022886 | 0 | 0.0645791 | 0.0601789 | 0.2109334 | 0 | 0.0083122 | 0.017927 | 0.0421127 | 0.0197614 | 0.0947428 |
| TCGA-G4-6306-01A | 0 | 0.0312464 | 0.0845735 | 0.2866183 | 0 | 0.1373503 | 0 | 0.1703401 | 0.0716171 | 0 | 0 | 0.0678304 | 0.0180274 | 0.032827 | 0.0628188 | 0.0202447 | 0.0048824 | 0.0049754 | 0.0066482 | 0 | 0 | 0 |
| TCGA-G4-6320-01A | 0.216014 | 0 | 0 | 0.1459987 | 0 | 0.1643715 | 0 | 0.0987819 | 0.014175 | 0 | 0.0806848 | 0 | 0.0515622 | 0.0606834 | 0.0508976 | 0.0222768 | 0 | 0 | 0 | 0.0751007 | 0.0194534 | 0 |
| TCGA-CK-6748-01A | 0.0013879 | 0.0019537 | 0 | 0.0276898 | 0 | 0.0162601 | 0.0208688 | 0.0062086 | 0.0263749 | 0 | 0.0607147 | 0 | 0 | 0.5516427 | 0 | 0.1952867 | 0 | 0 | 0 | 0.0916122 | 0 | 0 |
| TCGA-AA-3864-01A | 0.0739772 | 0 | 0.0179129 | 0.115354 | 0 | 0.0863536 | 0 | 0.1183064 | 0 | 0 | 0.0021704 | 0.02322 | 0 | 0.0998482 | 0.0590741 | 0.2841024 | 0 | 0 | 0 | 0.0859023 | 0 | 0.0337783 |
| TCGA-A6-6142-01A | 0.0845075 | 0 | 0.029572 | 0.0189719 | 0 | 0.0626399 | 0 | 0.0534864 | 0.0219924 | 0 | 0.0071404 | 0 | 0 | 0.3724614 | 0.0215092 | 0.1846614 | 0 | 0 | 0 | 0.1226309 | 0.0008989 | 0.0195278 |
| TCGA-AA-A01Q-01A | 0 | 0.0265408 | 0.0508466 | 0.1754991 | 0 | 0 | 0 | 0.2102517 | 0.0736595 | 0 | 0.0198711 | 0.0905721 | 0.0062455 | 0.0159773 | 0.063632 | 0.2456725 | 0.0145371 | 0 | 0 | 0.0066947 | 0 | 0 |
| TCGA-CA-5796-01A | 0.0978729 | 0 | 0.0995414 | 0.0742742 | 0 | 0.3912538 | 0 | 0.0067937 | 0.0699566 | 0 | 0 | 0.0255232 | 0.0116146 | 0.0160949 | 0.0093057 | 0.1699431 | 0.0147262 | 0 | 0 | 0.0130997 | 0 | 0 |
| TCGA-A6-2676-01A | 0 | 0 | 0.0081002 | 0.09994 | 0 | 0.0281773 | 0.1167391 | 0.0598555 | 0.0064316 | 0 | 0 | 0.0856517 | 0.0045507 | 0.0885143 | 0.0854405 | 0.3482737 | 0.0024013 | 0 | 0 | 0.0545749 | 0 | 0.0113493 |
| TCGA-A6-A567-01A | 0.0910916 | 0.0362833 | 0.0261226 | 0.0581652 | 0 | 0.1501318 | 0 | 0.1217391 | 0 | 0 | 0.0704848 | 0 | 0.0003833 | 0.0764196 | 0.0301965 | 0.3075827 | 0 | 0.0008417 | 0 | 0.0305579 | 0 | 0 |
| TCGA-AA-3713-01A | 0.1616366 | 0 | 0.108528 | 0.1006532 | 0 | 0.2094326 | 0 | 0.0416971 | 0.0135396 | 0 | 0.0557596 | 0.0095549 | 0 | 0.018117 | 0.0617624 | 0.2078378 | 0.0076963 | 0 | 0 | 0.0034849 | 0.0003 | 0 |
| TCGA-A6-6648-01A | 0.048523 | 0 | 0.0524111 | 0.1572967 | 0 | 0.3199775 | 0.0655992 | 0 | 0 | 0 | 0 | 0 | 0.0302874 | 0.0046769 | 0.0544784 | 0.1463007 | 0.0213289 | 0.010798 | 0 | 0.0815445 | 0.0067777 | 0 |
| TCGA-A6-A565-01A | 0.1924714 | 0.016275 | 0 | 0.1381142 | 0.0349429 | 0.2940726 | 0.0036864 | 0 | 0 | 0 | 0.0442904 | 0 | 0.0216352 | 0.0272047 | 0.0244191 | 0.1479788 | 0 | 0.0056535 | 0.025406 | 0.0238497 | 0 | 0 |
| TCGA-F4-6855-01A | 0.003821 | 0.0499369 | 0 | 0.065223 | 0 | 0.0996386 | 0 | 0.0299219 | 0.0500146 | 0 | 0.0031651 | 0.0332105 | 0.0851419 | 0.1231922 | 0.0133768 | 0.3204447 | 0.0017056 | 0 | 0 | 0.0856652 | 0.0355421 | 0 |
| TCGA-DM-A1D4-01A | 0.0200423 | 0 | 0.0615278 | 0.1718397 | 0 | 0.1905955 | 0 | 0.1933867 | 0.0429393 | 0 | 0 | 0.0678688 | 0.0350898 | 0.0474651 | 0.0320023 | 0.0437313 | 0.0059891 | 0 | 0 | 0.0849424 | 0.0025799 | 0 |
| TCGA-A6-2675-01A | 0.061832 | 0 | 0.0324901 | 0.0482974 | 0 | 0.0694135 | 0 | 0.0378804 | 0.0620345 | 0 | 0.0193112 | 0.0263199 | 0.0032638 | 0.1248872 | 0.0752592 | 0.3838933 | 0 | 0 | 0.0058025 | 0.0292804 | 0 | 0.0200346 |
| TCGA-CM-5861-01A | 0.0391716 | 0 | 0 | 0.2733522 | 0 | 0.0813611 | 0 | 0.0610122 | 0.0216222 | 0 | 0.0230367 | 0.06503 | 0.0307905 | 0.128417 | 0.1555656 | 0.0703924 | 0 | 0 | 0 | 0.0428265 | 0 | 0.0074219 |
| TCGA-DM-A1HB-01A | 0 | 0.0041019 | 0.01657 | 0.0456537 | 0 | 0.1435969 | 0 | 0.0417372 | 0.0230213 | 0 | 0.0089527 | 0.0175188 | 0 | 0.190116 | 0.017799 | 0.0878014 | 0.0091919 | 0 | 0 | 0.3939391 | 0 | 0 |
| TCGA-AY-A8YK-01A | 0 | 0.112981 | 0.0826686 | 0.0709745 | 0 | 0.209143 | 0.0580746 | 0 | 0.0331082 | 0 | 0.1539282 | 0 | 0.0183243 | 0.1461152 | 0 | 0.0307277 | 0 | 0.0346859 | 0 | 0.0492686 | 0 | 0 |
| TCGA-DM-A1D9-01A | 0 | 0.0253586 | 0.0531876 | 0.0806113 | 0 | 0.1579535 | 0.0094549 | 0.0229409 | 0 | 0 | 0.0215692 | 0.0244175 | 0 | 0.11209 | 0.0784973 | 0.2940908 | 0.0238418 | 0 | 0 | 0.0959866 | 0 | 0 |
| TCGA-AA-3666-01A | 0.0288901 | 0 | 0.0552531 | 0.027132 | 0 | 0.1524736 | 0.0412927 | 0.0864503 | 0 | 0 | 0.032294 | 0 | 0.0086635 | 0.0487869 | 0.0810692 | 0.2452746 | 0 | 0 | 0 | 0.1713024 | 0 | 0.0211175 |
| TCGA-AZ-4313-01A | 0 | 0 | 0.0201915 | 0.1912226 | 0 | 0.1208425 | 0 | 0.1546717 | 0.033584 | 0 | 0.0272721 | 0.0038104 | 0 | 0.2327851 | 0.0719055 | 0.0505765 | 0 | 0.0031331 | 0 | 0.090005 | 0 | 0 |
| TCGA-A6-6652-01A | 0.0074176 | 0.0074427 | 0 | 0.1196688 | 0 | 0.2140088 | 0 | 0.0110619 | 0.0067611 | 0 | 0.0862886 | 0 | 0.0338055 | 0.3287835 | 0 | 0 | 0.0027639 | 0.0119782 | 0 | 0.1700196 | 0 | 0 |
| TCGA-AY-4070-01A | 0.0417309 | 0.0009873 | 0.107235 | 0.1404498 | 0 | 0.2064211 | 0 | 0.2203391 | 0.0853773 | 0 | 0 | 0.01229 | 0.0146383 | 0.0271956 | 0.0349828 | 0.0666251 | 0.0021635 | 0.0153338 | 0 | 0.0242303 | 0 | 0 |
| TCGA-AA-A02W-01A | 0.0798071 | 0 | 0.1026653 | 0.1625557 | 0 | 0.1133959 | 0 | 0.1060937 | 0.0088669 | 0 | 0 | 0.0952984 | 0.0115652 | 0.0375362 | 0.081849 | 0.1355132 | 0.0248411 | 0 | 0.0400123 | 0 | 0 | 0 |
| TCGA-AZ-6605-01A | 0.0520838 | 0 | 0.0350341 | 0.0527311 | 0 | 0.0984446 | 0 | 0.0679636 | 0.0936043 | 0 | 0 | 0.0400727 | 0 | 0.2311128 | 0.063437 | 0.2215255 | 0 | 0 | 0.0439904 | 0 | 0 | 0 |
| TCGA-DM-A28C-01A | 0.0506973 | 0 | 0.0985927 | 0.0473247 | 0 | 0.2589792 | 0 | 0.026904 | 0 | 0 | 0.0359175 | 0 | 0.0036553 | 0.2399633 | 0.0366092 | 0.1653232 | 0 | 0 | 0 | 0.0360336 | 0 | 0 |
| TCGA-D5-6927-01A | 0.0422554 | 0 | 0.014572 | 0.1366514 | 0 | 0.1439595 | 0.0195801 | 0.1064264 | 0.0134278 | 0 | 0.0441941 | 0 | 0.009384 | 0.1345538 | 0.0922455 | 0.1393284 | 0 | 0 | 0 | 0.05271 | 0.0117079 | 0.0390037 |
| TCGA-AA-3980-01A | 0.0146593 | 0 | 0.0219433 | 0.2365549 | 0 | 0.0304282 | 0.0021951 | 0.1017994 | 0.1214681 | 0 | 0 | 0.0344574 | 0 | 0.0528675 | 0.050274 | 0.213078 | 0.0239906 | 0 | 0 | 0.0485462 | 0.0098734 | 0.0378647 |
| TCGA-AA-3681-01A | 0.0435769 | 0 | 0.0389857 | 0.1319461 | 0 | 0.0701702 | 0 | 0.1366683 | 0.0376621 | 0 | 0.0037572 | 0.023419 | 0.0010622 | 0.0969111 | 0.08309 | 0.2378416 | 0 | 0 | 0 | 0.0830897 | 0.0017065 | 0.0101133 |
| TCGA-AA-3492-01A | 0.0197171 | 0 | 0.0188855 | 0.1712066 | 0 | 0 | 0.0049408 | 0.0592156 | 0.0475449 | 0 | 0 | 0.055632 | 0.002767 | 0.1266977 | 0.0811818 | 0.3153116 | 0.0016562 | 0 | 0 | 0.089383 | 0 | 0.0058602 |
| TCGA-AA-3861-01A | 0.1654174 | 0 | 0.2099208 | 0.1251451 | 0 | 0.0927713 | 0 | 0.0574505 | 0.0712481 | 0 | 0 | 0.0175482 | 0.0285446 | 0 | 0.0374673 | 0.1061334 | 0.0118037 | 0 | 0 | 0.0635443 | 0.0130055 | 0 |
| TCGA-A6-2671-01A | 0.0253339 | 0.0146864 | 0 | 0.0343951 | 0 | 0.0547125 | 0 | 0.0544267 | 0.029077 | 0 | 0.0201666 | 0.0420206 | 0.050932 | 0.1713782 | 0.038385 | 0.342958 | 0 | 0 | 0 | 0.0959599 | 0 | 0.0255681 |
| TCGA-A6-6781-01A | 0.0189767 | 0 | 0.0021177 | 0.1342018 | 0 | 0.0782825 | 0 | 0.0601414 | 0.0094037 | 0 | 0.0171591 | 0.0172949 | 0.0485063 | 0.2617687 | 0.0536688 | 0.2555481 | 0 | 0 | 0 | 0.0239164 | 0 | 0.0190139 |
| TCGA-D5-6535-01A | 0 | 0.015185 | 0.0535591 | 0.0562078 | 0 | 0.2298387 | 0.0095064 | 0.0949062 | 0.0256071 | 0 | 0.023934 | 0 | 0 | 0.0790792 | 0.1352509 | 0.2483312 | 0 | 0 | 0 | 0.0239292 | 0 | 0.0046652 |
| TCGA-AA-A029-01A | 0 | 0.0375761 | 0.0325248 | 0.112108 | 0 | 0 | 0 | 0.1304641 | 0.0251915 | 0.0320397 | 0 | 0.0441124 | 0 | 0.1948927 | 0.0215691 | 0.1659789 | 0.0118171 | 0 | 0 | 0.1917257 | 0 | 0 |
| TCGA-AD-6963-01A | 0.0225697 | 0 | 0.0245559 | 0.2596908 | 0 | 0.0582148 | 0.070818 | 0.1125097 | 0 | 0 | 0.0190652 | 0 | 0.0076315 | 0.1113254 | 0.0920057 | 0.0902175 | 0.0016067 | 0 | 0 | 0.0852754 | 0 | 0.0445135 |
| TCGA-CM-4747-01A | 0.018806 | 0.0232627 | 0 | 0.0987975 | 0 | 0.1323356 | 0 | 0.0657101 | 0.0774303 | 0 | 0 | 0 | 0 | 0.2311197 | 0.0391568 | 0.3032706 | 0 | 0 | 0 | 0.0101107 | 0 | 0 |
| TCGA-NH-A50T-01A | 0 | 0.00258 | 0.0719342 | 0.044523 | 0.0945615 | 0.0269163 | 0 | 0.056313 | 0 | 0 | 0.0420654 | 0 | 0.0009825 | 0.1950073 | 0.0297722 | 0.2158996 | 0 | 0.0267483 | 0 | 0.1655684 | 0 | 0.0271282 |
| TCGA-CK-5914-01A | 0.0466896 | 0 | 0.0009303 | 0.2394832 | 0 | 0.0387718 | 0.0335085 | 0.0454578 | 0 | 0 | 0 | 0.067371 | 0.0251154 | 0.0273244 | 0.1207011 | 0.1792021 | 0 | 0 | 0 | 0.1754449 | 0 | 0 |
| TCGA-AA-A03J-01A | 0.0083381 | 0.0151766 | 0.0265777 | 0.2779039 | 0 | 0 | 0 | 0.0891879 | 0.0849207 | 0 | 0 | 0.0937936 | 0.0335598 | 0.034319 | 0.0786309 | 0.1966501 | 0.0186241 | 0 | 0 | 0.0423176 | 0 | 0 |
| TCGA-AA-3519-01A | 0.0006918 | 0.0083128 | 0.1306154 | 0.0758508 | 0.061089 | 0.0181803 | 0 | 0.0472215 | 0.0478884 | 0 | 0.0239825 | 0 | 0.0669979 | 0 | 0.0333002 | 0.3055695 | 0 | 0.042282 | 0 | 0.1380179 | 0 | 0 |
| TCGA-AA-3518-01A | 0.0754965 | 0 | 0.050715 | 0.1064444 | 0 | 0.1320004 | 0.0209237 | 0.0877779 | 0.037153 | 0 | 0.0313941 | 0.0176066 | 0 | 0.087108 | 0.073853 | 0.2030579 | 0 | 0 | 0 | 0.0669769 | 0 | 0.0094926 |
| TCGA-AA-A01T-01A | 0.0268776 | 0 | 0.1292745 | 0.1222206 | 0 | 0 | 0 | 0.073164 | 0.2274953 | 0 | 0.0156784 | 0.0001785 | 0.0125526 | 0.0651265 | 0.0951968 | 0.1097131 | 0 | 0.0090319 | 0 | 0.1134902 | 0 | 0 |
| TCGA-CM-6678-01A | 0.0352298 | 0 | 0.011781 | 0.041605 | 0 | 0.1274044 | 0 | 0.0127147 | 0.0839582 | 0 | 0.0856842 | 0 | 0 | 0.2902662 | 0.0057824 | 0.0726805 | 0 | 0 | 0 | 0.2328936 | 0 | 0 |
| TCGA-A6-6651-01A | 0.1138124 | 0 | 0.0940424 | 0.0493286 | 0 | 0.0982566 | 0 | 0.0347081 | 0.0389561 | 0 | 0.0027391 | 0 | 0.0087643 | 0.2470034 | 0.0281807 | 0.1987094 | 0.0117919 | 0 | 0.0737071 | 0 | 0 | 0 |
| TCGA-AA-3850-01A | 0 | 0.0181785 | 0.2379761 | 0.0597696 | 0 | 0.1784668 | 0 | 0.0789803 | 0.0723201 | 0 | 0.0025881 | 0 | 0.0121675 | 0.0324653 | 0.022465 | 0.225899 | 0 | 0 | 0 | 0.0403956 | 0.0035588 | 0.0147693 |
| TCGA-AA-3972-01A | 0.0073584 | 0 | 0.0069535 | 0.097851 | 0 | 0.1816702 | 0 | 0.0288846 | 0.0095122 | 0 | 0 | 0.0029956 | 0.063446 | 0.0404809 | 0 | 0.4008894 | 0.0066803 | 0.0378543 | 0 | 0.0799999 | 0 | 0.0354238 |
| TCGA-AA-3812-01A | 0.0060072 | 0.0131016 | 0.0044942 | 0.0066782 | 0.0007539 | 0 | 0 | 0.0170611 | 0.0435643 | 0.0074592 | 0.0418983 | 0 | 0 | 0.7159259 | 0.0101296 | 0.0200084 | 0 | 0 | 0 | 0.1129183 | 0 | 0 |
| TCGA-AY-A69D-01A | 0.092634 | 0.0539368 | 0.0419651 | 0.0417472 | 0 | 0.1532461 | 0 | 0.1122503 | 0.0076753 | 0 | 0.0780981 | 0 | 0.0233874 | 0.0259892 | 0.0038228 | 0.1914984 | 0 | 0.035906 | 0 | 0.1378432 | 0 | 0 |
| TCGA-AA-A01Z-01A | 0.006595 | 0.0210654 | 0.0440999 | 0.0330472 | 0 | 0.2304434 | 0 | 0.1295489 | 0 | 0 | 0.0222509 | 0.0222293 | 0 | 0.1295073 | 0 | 0.1950843 | 0 | 0.0018015 | 0 | 0.1643268 | 0 | 0 |
| TCGA-A6-3810-01B | 0.0405154 | 0 | 0 | 0 | 0.0045671 | 0.303112 | 0.0024483 | 0.0131634 | 0 | 0 | 0.0443371 | 0 | 0 | 0.0798151 | 0.1454484 | 0.2082179 | 0 | 0 | 0 | 0.0535459 | 0.0732538 | 0.0315756 |
| TCGA-D5-6530-01A | 0.0127992 | 0 | 0.0266828 | 0.120999 | 0 | 0.1646016 | 0.0347019 | 0.0960651 | 0.0072217 | 0 | 0.0469817 | 0.0035717 | 0 | 0.1553368 | 0.1205449 | 0.1785816 | 0 | 0 | 0 | 0.0224171 | 0 | 0.0094949 |
| TCGA-AA-3525-01A | 0.1872274 | 0 | 0.2035724 | 0.1051937 | 0 | 0.0497084 | 0 | 0.033167 | 0.0108191 | 0 | 0 | 0.0388153 | 0 | 0.0916665 | 0.0410462 | 0.1801018 | 0 | 0 | 0 | 0.0376916 | 0 | 0.0209906 |
| TCGA-SS-A7HO-01A | 0 | 0.0484274 | 0 | 0.1736319 | 0.0139285 | 0 | 0.0195095 | 0.0901566 | 0 | 0 | 0.1246963 | 0 | 0.0769252 | 0 | 0.000886 | 0.292344 | 0 | 0.0050106 | 0 | 0.1544841 | 0 | 0 |
| TCGA-D5-6928-01A | 0.0066103 | 0.0677226 | 0 | 0.2535696 | 0 | 0.0347889 | 0 | 0.1239316 | 0.0737537 | 0 | 0.012746 | 0.0324009 | 0.0106711 | 0.0815927 | 0.1070124 | 0.173883 | 0 | 0 | 0.0094792 | 0 | 0 | 0.0118379 |
| TCGA-G4-6315-01A | 0.2077667 | 0 | 0.1201851 | 0.1383014 | 0 | 0.1395741 | 0.0065359 | 0.1125196 | 0 | 0 | 0.0221674 | 0.0661796 | 0.0382201 | 0.024166 | 0.0073292 | 0.0719711 | 0.0008027 | 0.0098112 | 0.0344698 | 0 | 0 | 0 |
| TCGA-D5-6534-01A | 0.0195671 | 0.0021174 | 0.0003119 | 0.0514194 | 0 | 0.1495559 | 0 | 0.0645089 | 0.0777013 | 0 | 0 | 0.022124 | 0.0146997 | 0.1290693 | 0.0825889 | 0.2460803 | 0.0936658 | 0 | 0.0441696 | 0 | 0 | 0.0024205 |
| TCGA-A6-3808-01A | 0.090449 | 0 | 0.0429178 | 0.0859887 | 0 | 0.0932027 | 0 | 0.0641278 | 0.0349758 | 0 | 0 | 0.0372837 | 0.0486711 | 0.0872225 | 0.0390887 | 0.2033995 | 0.0215465 | 0 | 0.0289647 | 0.0680857 | 0.0337894 | 0.0202864 |
| TCGA-AA-3664-01A | 0.0140583 | 0 | 0.0498412 | 0.104073 | 0 | 0.1680519 | 0 | 0.1430461 | 0.0030458 | 0 | 0 | 0.0333849 | 0 | 0.1352228 | 0.0682048 | 0.2081035 | 0 | 0 | 0 | 0.070789 | 0 | 0.0021787 |
| TCGA-G4-6303-01A | 0.0285369 | 0 | 0.0399049 | 0.0563251 | 0 | 0.2230522 | 0 | 0.0111012 | 0.0269541 | 0 | 0.0254875 | 0 | 0.0117695 | 0.1420398 | 0.026866 | 0.1958536 | 0.0401526 | 0.0322084 | 0 | 0.1137685 | 0.0157956 | 0.0101839 |
| TCGA-AA-3692-01A | 0.0185662 | 0 | 0.0210114 | 0.0354243 | 0 | 0.1440876 | 0.0059921 | 0.0497474 | 0.0397337 | 0 | 0.0048416 | 0.0015989 | 0 | 0.1522233 | 0.0187889 | 0.2146138 | 0.0111043 | 0 | 0 | 0.215946 | 0.0498209 | 0.0164996 |
| TCGA-AA-3968-01A | 0.0056665 | 0.047584 | 0.0222841 | 0.1400398 | 0 | 0.0518892 | 0 | 0.0837852 | 0.0793646 | 0 | 0 | 0.0366167 | 0.0282555 | 0.0543196 | 0.070231 | 0.285729 | 0.0304528 | 0 | 0 | 0.0637818 | 0 | 0 |
| TCGA-AA-3867-01A | 0.0529981 | 0.0162898 | 0.0046692 | 0.065803 | 0 | 0.0819145 | 0 | 0.0882513 | 0.0709413 | 0 | 0 | 0 | 0.026771 | 0.0435593 | 0.0401866 | 0.4709365 | 0.0022434 | 0 | 0 | 0.0354361 | 0 | 0 |
| TCGA-F4-6805-01A | 0.020697 | 0.0232204 | 0 | 0.0658841 | 0 | 0.1817458 | 0 | 0.0421342 | 0.0868912 | 0 | 0 | 0.0417463 | 0.0409088 | 0 | 0.0292046 | 0.2892682 | 0.073513 | 0.0123887 | 0 | 0.0915504 | 0.0008473 | 0 |
| TCGA-AA-3560-01A | 0.0251876 | 0 | 0.0464437 | 0.0364793 | 0 | 0.2484982 | 0 | 0.0294981 | 0.0588294 | 0 | 0.0481884 | 0 | 0.005404 | 0.0427804 | 0.0501696 | 0.2182804 | 0.0782762 | 0.0151622 | 0 | 0.0663695 | 0.0081603 | 0.0222726 |
| TCGA-D5-5537-01A | 0.0040104 | 0.0499956 | 0.0866933 | 0.105747 | 0 | 0.2112213 | 0 | 0.0124928 | 0.0270161 | 0 | 0.0332927 | 0.002504 | 0.0821313 | 0.0792115 | 0.0234032 | 0.2028231 | 0.0050863 | 0 | 0 | 0.0586097 | 0.0157616 | 0 |
| TCGA-CM-5862-01A | 0.0281648 | 0 | 0.0349443 | 0.064193 | 0.0426186 | 0 | 0 | 0.118618 | 0 | 0 | 0.0172632 | 0.062704 | 0.0153189 | 0.1894935 | 0.0677881 | 0.3047091 | 0 | 0 | 0 | 0.0541845 | 0 | 0 |
| TCGA-A6-5659-01B | 0 | 0.0270023 | 0 | 0.0135164 | 0.1431872 | 0.2609185 | 0.1162605 | 0 | 0 | 0 | 0.0853243 | 0 | 0.0281574 | 0 | 0.0318274 | 0.130956 | 0.0083112 | 0.0504352 | 0 | 0.1041035 | 0 | 0 |
| TCGA-D5-6932-01A | 0.0271197 | 0 | 0.0015865 | 0.0861015 | 0 | 0.136387 | 0 | 0.1007165 | 0.0432165 | 0 | 0 | 0.0343225 | 0 | 0.1655349 | 0.038944 | 0.3181229 | 0 | 0.0033633 | 0 | 0.0392751 | 0 | 0.0053095 |
| TCGA-AA-3982-01A | 0.044792 | 0 | 0.0119052 | 0.1421518 | 0 | 0.1194967 | 0 | 0.0876613 | 0.0907763 | 0 | 0 | 0.0209655 | 0 | 0.0654259 | 0.0334015 | 0.3158359 | 0.0456544 | 0.0016888 | 0.0149198 | 0 | 0 | 0.005325 |
| TCGA-DM-A28A-01A | 0.036788 | 0 | 0.0228957 | 0.1379311 | 0 | 0.0084806 | 0 | 0.1258773 | 0 | 0 | 0.0068401 | 0.0382924 | 3.25E-05 | 0.1058491 | 0.1107757 | 0.378816 | 0 | 0 | 0 | 0.0274216 | 0 | 0 |
| TCGA-D5-6930-01A | 0.1382994 | 0 | 0 | 0.1345091 | 0 | 0.1250731 | 0 | 0.1211702 | 0.0371469 | 0 | 0 | 0.0319916 | 0 | 0.1297207 | 0.1080249 | 0.1090098 | 0 | 0 | 0 | 0.0650544 | 0 | 0 |
| TCGA-D5-6529-01A | 0.0069347 | 0.0338439 | 0.1126853 | 0.0632736 | 0 | 0.175911 | 0 | 0.1064181 | 0.024674 | 0 | 0 | 0.0073623 | 0.0020389 | 0.1017237 | 0.0653402 | 0.2524384 | 0 | 0 | 0 | 0.0370065 | 0 | 0.0103494 |
| TCGA-A6-2683-01A | 0.0315206 | 0 | 0.2182531 | 0.0688424 | 0 | 0.0964389 | 0 | 0.1484854 | 0.0380527 | 0 | 0.0397682 | 0 | 0.0314121 | 0.0160366 | 0.0076907 | 0.1981313 | 0.0070075 | 0 | 0 | 0.0983605 | 0 | 0 |
| TCGA-CK-5913-01A | 0.0669978 | 0 | 0.0036427 | 0.0901242 | 0 | 0.0836114 | 0.0385151 | 0.132896 | 0.0162123 | 0 | 0.0116799 | 0.0045559 | 0 | 0.1097106 | 0.116303 | 0.2194831 | 0.0332837 | 0.0071176 | 0 | 0.0190124 | 0 | 0.0468541 |
| TCGA-AA-3496-01A | 0.0692917 | 0 | 0.019723 | 0.0625433 | 0 | 0.1645483 | 0.0133467 | 0.0550401 | 0.0052171 | 0 | 0.0208145 | 0 | 0 | 0.1397774 | 0.0685545 | 0.3688079 | 0 | 0 | 0 | 0.0027132 | 0 | 0.0096221 |
| TCGA-G4-6311-01A | 0.1965978 | 0 | 0 | 0.0195474 | 0 | 0.0671595 | 0.0240409 | 0.0250568 | 0.0226075 | 0 | 0.0480489 | 0 | 0 | 0.3690016 | 0.0124087 | 0.1400497 | 0 | 0 | 0 | 0.0734879 | 0 | 0.0019934 |
| TCGA-D5-5540-01A | 0.0230116 | 0 | 0 | 0.1475171 | 0 | 0.1194101 | 0.0709697 | 0.0463519 | 0 | 0 | 0.0022579 | 0 | 0 | 0 | 0.1228378 | 0.2803676 | 0 | 0.005928 | 0 | 0.1789719 | 0 | 0.0023765 |
| TCGA-AD-6901-01A | 0 | 0.006852 | 0.0156559 | 0.1078672 | 0 | 0.0869171 | 0 | 0.0637241 | 0.0625087 | 0 | 0 | 0.052856 | 0.0079583 | 0.1690154 | 0.1216823 | 0.254078 | 0.0011044 | 0 | 0.0497807 | 0 | 0 | 0 |
| TCGA-AA-A01F-01A | 0.0004192 | 0 | 0 | 0.0694666 | 0 | 0.1656751 | 0.103281 | 0.0500202 | 0.1380033 | 0 | 0 | 0.0584034 | 0.0307433 | 0.1312383 | 0.0221956 | 0.0981481 | 0 | 0 | 0 | 0.1324058 | 0 | 0 |
| TCGA-AA-3552-01A | 0.1413728 | 0 | 0.2215688 | 0.0850168 | 0 | 0.0839328 | 0 | 0.0714215 | 0.061122 | 0 | 0 | 0.0344402 | 0.0162761 | 0.0172954 | 0.0460515 | 0.1741369 | 0.0320483 | 0 | 0.015317 | 0 | 0 | 0 |
| TCGA-A6-5660-01A | 0.0384229 | 0 | 0 | 0.0561025 | 0 | 0.1166135 | 0.0565007 | 0.0025539 | 0.0181479 | 0 | 0.0480835 | 0 | 0 | 0.2733461 | 0.0069913 | 0.2933691 | 0.0199778 | 0 | 0 | 0.0682071 | 0.0016838 | 0 |
| TCGA-AD-5900-01A | 0.091864 | 0 | 0.0136031 | 0.2340053 | 0 | 0.0414534 | 0 | 0.0798515 | 0.0614691 | 0 | 0 | 0.0529255 | 0 | 0.079686 | 0.0870018 | 0.2206345 | 0 | 0 | 0.0375057 | 0 | 0 | 0 |
| TCGA-AA-A01I-01A | 0.0141299 | 0.0014082 | 0.042033 | 0.0679242 | 0 | 0.2672956 | 0 | 0.0138964 | 0 | 0 | 0 | 0.0137664 | 0.0401509 | 0 | 0.0785401 | 0.1778628 | 0.0998478 | 0 | 0 | 0.1670737 | 0.016071 | 0 |
| TCGA-AA-A03F-01A | 0 | 0.058473 | 0.2113894 | 0.0732178 | 0 | 0.0766109 | 0 | 0.042225 | 0.045536 | 0 | 0.1001949 | 0 | 0.0028161 | 0.0922402 | 0 | 0 | 0.0014999 | 0.0440154 | 0 | 0.2466494 | 0 | 0.005132 |
| TCGA-AZ-6601-01A | 0.0721233 | 0 | 0 | 0.0830773 | 0 | 0.1631131 | 0 | 0.0893026 | 0.0321197 | 0 | 0 | 0.040174 | 0.0273198 | 0.1081982 | 0.1058328 | 0.2520678 | 0 | 0 | 0 | 0.0266714 | 0 | 0 |
| TCGA-3L-AA1B-01A | 0.0545744 | 0.0822629 | 0.0507097 | 0.0676932 | 0 | 0.1705801 | 0.0026414 | 0.0662754 | 0.0734524 | 0 | 0.0968487 | 0.0030837 | 0.0718637 | 0.0827265 | 0.0132524 | 0.0959919 | 0.0140626 | 0 | 0 | 0.0539809 | 0 | 0 |
| TCGA-AA-3693-01A | 0.0142861 | 0 | 0.0386863 | 0.1033411 | 0 | 0.2138774 | 0.028242 | 0.0130726 | 0 | 0 | 0.0455565 | 0 | 0.0139745 | 0.0692578 | 0.02974 | 0.3035256 | 0.05957 | 0.0048908 | 0.0117624 | 0.0435304 | 0.0066866 | 0 |
| TCGA-AA-3502-01A | 0.011725 | 0.0330762 | 0.0204515 | 0.2266242 | 0 | 0.1997086 | 0 | 0.0559414 | 0.0387908 | 0 | 0.0230275 | 0 | 0.0500625 | 0 | 0.0097395 | 0.2409115 | 0.0206305 | 0 | 0 | 0.0622481 | 0.0070626 | 0 |
| TCGA-AA-A02E-01A | 0 | 0.0053627 | 0.092157 | 0.1694087 | 0 | 0.0058341 | 0 | 0.0963538 | 0.1344771 | 0 | 0 | 0.0400629 | 0.0167096 | 0.0900657 | 0.0031103 | 0.2558156 | 0.0127183 | 0.0113706 | 0 | 0.0662782 | 0.0002754 | 0 |
| TCGA-A6-5661-01B | 0.0021214 | 0.0194546 | 0.0334316 | 0.0946547 | 0 | 0.1675388 | 0.0003721 | 0.1265189 | 0 | 0 | 0 | 0 | 0 | 0.0455451 | 0.0446401 | 0.28889 | 0 | 0.0124747 | 0 | 0.1234687 | 0.0175342 | 0.0233551 |
| TCGA-G4-6295-01A | 0.0564279 | 0.0087568 | 0 | 0.276499 | 0 | 0 | 0.0062458 | 0.1344725 | 0.0147312 | 0 | 0.0399592 | 0.0228471 | 0.046968 | 0 | 0.133716 | 0.1957911 | 0.0203142 | 0 | 0 | 0.0408623 | 0.0024091 | 0 |
| TCGA-AA-A01C-01A | 0.0629948 | 0 | 0.0672128 | 0.1242529 | 0 | 0.055007 | 0 | 0.180812 | 0.0086761 | 0 | 0 | 0.0833666 | 0.0081054 | 0.0667167 | 0.0748568 | 0.2641605 | 0 | 0 | 0 | 0.0038384 | 0 | 0 |
| TCGA-G4-6588-01A | 0 | 0 | 0.0237885 | 0.0416769 | 0 | 0.0881215 | 0 | 0.0393381 | 0.0290591 | 0 | 0.0022693 | 0.024857 | 0 | 0.3054319 | 0.0581778 | 0.1763237 | 0 | 0 | 0 | 0.1818063 | 0 | 0.0291498 |
| TCGA-A6-2680-01A | 0.0055227 | 0.0448455 | 0.0921272 | 0.1073496 | 0 | 0.0768671 | 0 | 0.0722651 | 0.0052542 | 0 | 0 | 0.0033987 | 0.0915534 | 0.1381989 | 0.0308782 | 0.1754747 | 0.0183368 | 0.0046695 | 0 | 0.1332586 | 0 | 0 |
| TCGA-AA-3977-01A | 0.0247282 | 0 | 0.0337387 | 0.1180552 | 0 | 0 | 0.0214799 | 0.1342456 | 0.0264458 | 0 | 0 | 0.0254559 | 0 | 0.0876004 | 0.0726208 | 0.26386 | 0.0340384 | 0 | 0 | 0.1500064 | 0 | 0.0077248 |
| TCGA-AA-3561-01A | 0.0793857 | 0 | 0.0217305 | 0.019943 | 0 | 0.1553799 | 0.049777 | 0.096992 | 0 | 0 | 0.0296988 | 0 | 0.005081 | 0.0456253 | 0.0884927 | 0.2978218 | 0 | 0.0157622 | 0 | 0.0614546 | 0 | 0.0328557 |
| TCGA-AA-3973-01A | 0.0373859 | 0.054613 | 0.0205405 | 0.1349983 | 0 | 0.0531153 | 0.0051636 | 0.0580792 | 0.0933858 | 0 | 0.0452737 | 0 | 0.0537825 | 0.2381859 | 0.0135733 | 0.0856756 | 0.0126593 | 0 | 0 | 0.093568 | 0 | 0 |
| TCGA-A6-6653-01A | 0.0380565 | 0 | 0.0043829 | 0.0239067 | 0 | 0.2037856 | 0 | 0.0072038 | 0.0367662 | 0 | 0.0173824 | 0.0623144 | 0 | 0.3462216 | 0.0432152 | 0.0801145 | 0.0064234 | 0 | 0 | 0.1231833 | 0 | 0.0070434 |
| TCGA-AA-3555-01A | 0.0424711 | 0 | 0.0017787 | 0.0370007 | 0 | 0.1436887 | 0 | 0.0143671 | 0 | 0 | 0.0433496 | 0.0076242 | 0.0196636 | 0.0998202 | 0.0742379 | 0.2466599 | 0 | 0.0217059 | 0 | 0.1002581 | 0 | 0.1473742 |
| TCGA-G5-6572-02A | 0.0852507 | 0 | 0.0146103 | 0.1434066 | 0 | 0.1494646 | 0 | 0.0191585 | 0.0580902 | 0 | 0 | 0.0019067 | 0.0734233 | 0.0552343 | 0.0368109 | 0.2840549 | 0.0006751 | 0 | 0.0748779 | 0 | 0 | 0.003036 |
| TCGA-G4-6317-02A | 0.0026187 | 0.0670167 | 0 | 0 | 0.0353037 | 0 | 0.0194185 | 0 | 0.0048344 | 0 | 0.0229259 | 0 | 0 | 0.6033019 | 0.003281 | 0.1941279 | 0 | 0 | 0 | 0.0471713 | 0 | 0 |
| TCGA-NH-A8F7-06A | 0 | 0.0547722 | 0.018013 | 0 | 0.1531619 | 0.0943552 | 0 | 0 | 0 | 0 | 0.0221876 | 0.0316031 | 0.0360947 | 0.1540434 | 0 | 0.400605 | 0 | 0 | 0 | 0.0351638 | 0 | 0 |
